# Landscape of parental postzygotic mutations across >11,000 rare disease trios

**DOI:** 10.1016/j.ajhg.2026.06.015

**Published:** 2026-07-13

**Authors:** O. Isaac Garcia-Salinas, Katrina A. Andrews, Rashesh Sanghvi, John A. Sayer, Maria Torra I Benach, My H. Pham, Aylwyn Scally, Hilary C. Martin, Raheleh Rahbari

**Affiliations:** 1 Wellcome Sanger Institute, Wellcome Genome Campus, Hinxton, UK; 2 Biosciences Institute, Faculty of Medical Sciences, Newcastle University, Renal Services, The Newcastle upon Tyne Hospitals NHS Foundation Trust, NIHR Newcastle Biomedical Research Centre, Newcastle upon Tyne, UK; 3 Department of Genetics, University of Cambridge, Cambridge, UK

**Keywords:** postzygotic mosaicism, early embryonic mutations, whole-genome sequencing, trio sequencing, germline mutation, rare disease genetics

## Abstract

Early postzygotic mutations (PZMs) that arise after fertilization but prior to primordial germ cell specification may be present in both somatic and germ cells, causing mosaicism in a parent and constitutive inheritance in their offspring. In clinical family-trio whole-genome sequencing (WGS), such variants are systematically missed because their sub-heterozygous variant allele fraction (VAF) prevents heterozygous calling in the parent, while residual parental allele support disqualifies the variant as a candidate germline *de novo* mutation (DNM) in the child. Here, we developed a bioinformatic approach to ascertain parental PZMs from unfiltered DNM candidates in standard-depth (∼30×) trio WGS and applied it to 12,015 trios from the Genomics England 100,000 Genomes Project. We identified 1,015 high-confidence early autosomal parental PZMs, a large single-source catalog of this mutation class. These exhibited a monomodal VAF distribution centered around 5% in parental blood, consistent with empirically characterized ascertainment boundaries imposed by standard-depth sequencing and germline variant calling. PZMs showed no parental age or sex bias and displayed a mutational spectrum distinct from that of DNMs, with enrichment for C>A and T>A substitutions and depletion of T>C. Mutational signature analysis revealed that both mutation types are shaped by clock-like signatures SBS1 and SBS5 in similar proportions, suggesting that spectral differences reflect shifts within shared mutagenic processes. Exploratory genomic distribution analysis revealed a negative PZM association with GC content, in contrast to the positive association for DNMs. Among these, we found variants in *DYNC1H1* and *WT1* with potential clinical relevance that were missed by routine diagnostic pipelines.

## Teaser

Garcia-Salinas et al. detect early parental postzygotic mutations from standard-depth trio WGS across 12,015 rare disease trios, identifying 1,015 high-confidence events. Their catalog reveals distinct mutational features of early embryonic mutagenesis and demonstrates that parental mosaicism can be recovered at scale with implications for diagnosis and recurrence risk.

## Main text

**Figure 1:**
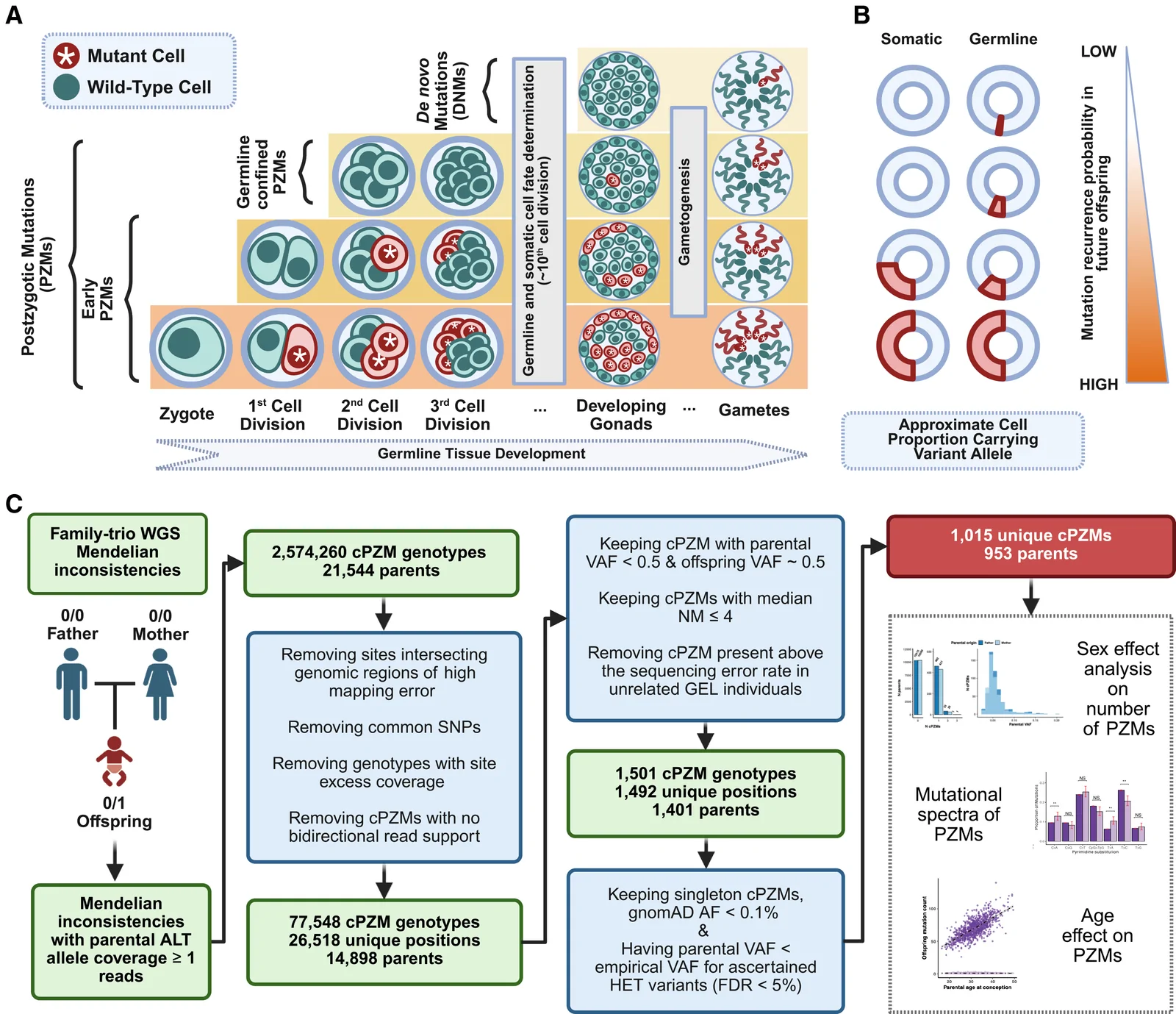
Origins of germline mutations and bioinformatic ascertainment of parental postzygotic mutations (A) Classification of germline mutations according to mutation timing. Postzygotic mutations (PZMs; rows 1–3, bottom to top) can affect both somatic and germline lineages if they occur before segregation of somatic and germline fates (i.e., early PZMs) or be restricted to the germline tissue if they arise in germline progenitors (i.e., germline-confined PZMs). For both categories, the earlier a mutation occurs, the larger the fraction of future gametes that will carry it. In contrast, *de novo* mutations (DNMs,; top row) arise later in life during gametogenesis and affect only a subset of germline progenitors or single gametes. Gametes are represented with sperm cells; however, germline mutations can arise in both the male and female germline. (B) Approximate proportions of somatic and germline cells carrying each type of mutation, with the right-hand scale indicating the likelihood of transmission to offspring. (C) Overview of this study: analysis of trio WGS data for Mendelian inconsistencies supported by ≥ 1 ALT read, yielding 1,015 early candidate PZMs (cPZMs) in parents. Created in BioRender (https://BioRender.com/zrncn74).

Most heritable mutations arise in adult germ cells throughout aging and gametogenesis and are known as germline *de novo* mutations (DNMs).^1^^,^^2^ However, mutations can also arise as early as the first zygotic cell division. These are termed postzygotic mutations (PZMs).^1^^,^^3^^,^^4^^,^^5^ Depending on developmental timing, PZMs can be classified into two categories. Early PZMs arise before somatic and germline fates are specified (approximately 2 weeks post-fertilization and within the first 10–15 cell divisions)^6^^,^^7^ and generate mosaicism in both compartments when the mutation was acquired by a cell progenitor of both lineages. Germline-confined PZMs occur later in germline progenitors and are restricted to the germline lineage. Importantly, the level of mosaicism in fully developed tissues reflects the timing of the mutation, with earlier events present at higher cell fractions and more likely to recur in the mosaic individual’s future offspring^8^^,^^9^ (Figures 1A and 1B). In a family trio, PZMs arising in either a parent or their child can be misidentified as classical DNMs in the offspring or discarded as sequencing artifacts. Here, we focus on parental PZMs, where the mutation arose during the parent’s early development and was subsequently transmitted to the offspring.

Although parental PZMs are a recognized source of sporadic genetic disease,^4^^,^^9^^,^^10^ they are systematically overlooked by standard germline variant-calling pipelines, which assume constitutive heterozygous allele fractions in an individual and therefore fail to detect variants present at mosaic levels.^11^ This limitation has particular consequences in the clinical sequencing of family trios (typically an affected child and two unaffected parents), where pathogenic parental PZMs escape detection through two distinct filtering mechanisms. First, because the mosaic parent usually carries the variant below the heterozygous detection threshold, it is not called as a parental genotype, yet the residual allelic support in that parent is sufficient to disqualify the variant as a DNM in the child, which requires near-absent parental evidence. Second, even in rare cases where the mosaic parent is clinically affected, the sub-heterozygous variant allele fraction (VAF) may still fall below the calling threshold for standard heterozygous variant detection. In both scenarios, the variant escapes clinical interpretation entirely, and recurrence risk to future offspring is underestimated or not communicated at all.^8^^,^^9^^,^^12^

One approach to recovering parental PZMs from trio data is to analyze the parental VAF of variants initially flagged as candidate DNMs by standard pipelines, where non-zero alternative-allele support in a parent suggests postzygotic origin followed by transmission to the offspring.^4^^,^^13^ However, at standard whole-genome sequencing (WGS) depth, low-VAF variants are difficult to distinguish from sequencing and mapping artifacts, particularly in regions with poor coverage.^13^^,^^14^ Statistical frameworks that integrate features such as read depth, allele balance, and quality scores can improve this discrimination,^15^ but robust detection at the genome-wide scale ultimately requires either deep sequencing-based validation or extended family designs involving multiple generations or siblings, neither of which is readily scalable to large cohorts. Consequently, only three studies have attempted systematic characterization of early parental PZMs to date. Rahbari et al.^1^ identified 24 PZMs across 6 parents across a multi-sibling design, while Sasani et al.^16^ and Jónsson et al.^17^ cataloged 442 and 523 autosomal single-nucleotide variant (SNV) PZMs from 69 and 451 parents, respectively, using extended family structures (supplemental material and methods). Collectively, these studies estimate between 1.15 and 6.4 early PZMs per individual but differ substantially in sequencing strategy, family structure, and variant filtering, limiting direct comparison.

At current scales, many genomic features of parental PZMs remain incompletely characterized. The mutational spectrum, which reflects the genomic context of mutagenesis and is informative of underlying processes,^18^ illustrates this limitation. The two largest of these catalogs have shown that PZMs ascertained across different developmental windows exhibit distinct spectral features, with substitution-type composition varying between pre-PGC and germline-confined mutations.^16^^,^^17^ However, these differences are often subtle,^19^ and resolving them requires catalogs with sufficient power to sample PZMs across the relevant VAF range, a constraint that existing datasets have only partially addressed.

To address these limitations, we developed a bioinformatic annotation approach to detect early parental PZMs in large family-trio cohorts (Figure 1C). Our method filters candidate DNMs within two-generation trios, retaining those in which the alternate allele is detectable in parental reads while minimizing artifacts. We applied this pipeline to 12,015 trios from the Genomics England (GEL) 100,000 Genomes Project (100kGP), an initiative aiming to identify the genetic causes of rare diseases through WGS in individuals from the United Kingdom.^20^ The original GEL analysis of germline variation did not include methods for ascertaining parental PZMs, making this cohort well suited for retrospective recovery of these variants. From this dataset, we identified 1,015 high-confidence early autosomal PZMs (Figure 1C), characterized their mutational spectrum and genomic distribution, and assessed their clinical relevance in the context of rare disease diagnosis, providing further evidence that parental mosaicism represents an underrecognized inheritance mode with direct implications for diagnostic yield and recurrence risk estimation.

We accessed a previously identified set of 5,720,018 autosomal SNVs exhibiting Mendelian inconsistencies across an initial set of 11,996 trios and 21,644 unique parents (after accounting for parents sequenced as part of multiple trios), recruited through the rare disease arm of the 100kGP^21^ (supplemental material and methods). Mendelian inconsistencies were defined as sites where the offspring was called as heterozygous for the alternative allele, while both parents were called as homozygous for the reference allele. These sites are also referred to as candidate DNMs since they are the starting point for DNM ascertainment in family sequencing studies. In GEL, these were identified on a per-trio basis using the variant caller Platypus, combining local realignment, haplotype assembly, and Bayesian statistical modeling.^22^

Among these, 2,627,735 had alternative allele read support in a single parent, suggesting parental postzygotic mosaicism, hereafter referred to as candidate PZMs (cPZMs). Because parents with multiple sequenced offspring could contribute the same cPZM across different trios, we retained a single record for each unique parent-variant combination, yielding an initial set of 2,574,260 cPZMs (mean = 118.93 cPZMs per parent). This set was then subjected to a custom filtering pipeline to distinguish true PZMs from sequencing artifacts and common germline polymorphisms (supplemental material and methods; Figures S1–S8), obtaining 1,015 high-confidence cPZMs, corresponding to 0.039% of the initial candidates (Figure S9).

After excluding individuals with missing metadata (*n* = 123; supplemental material and methods), we identified an average of 0.046 PZMs per parent across the remaining cohort, substantially lower than previous whole-genome estimates ranging from 1.15 to 6.4 PZMs per parent^1^^,^^16^^,^^17^ (supplemental material and methods). However, this difference is expected since previous studies actively enriched for PZM detection through deep sequencing or additional family members, whereas our cohort-scale approach relies on standard sequencing depth without multigenerational or sibling data, limiting sensitivity to variants with lower somatic VAF or those approaching constitutive heterozygosity. Our per-parent estimate is therefore likely conservative.

**Figure 2:**
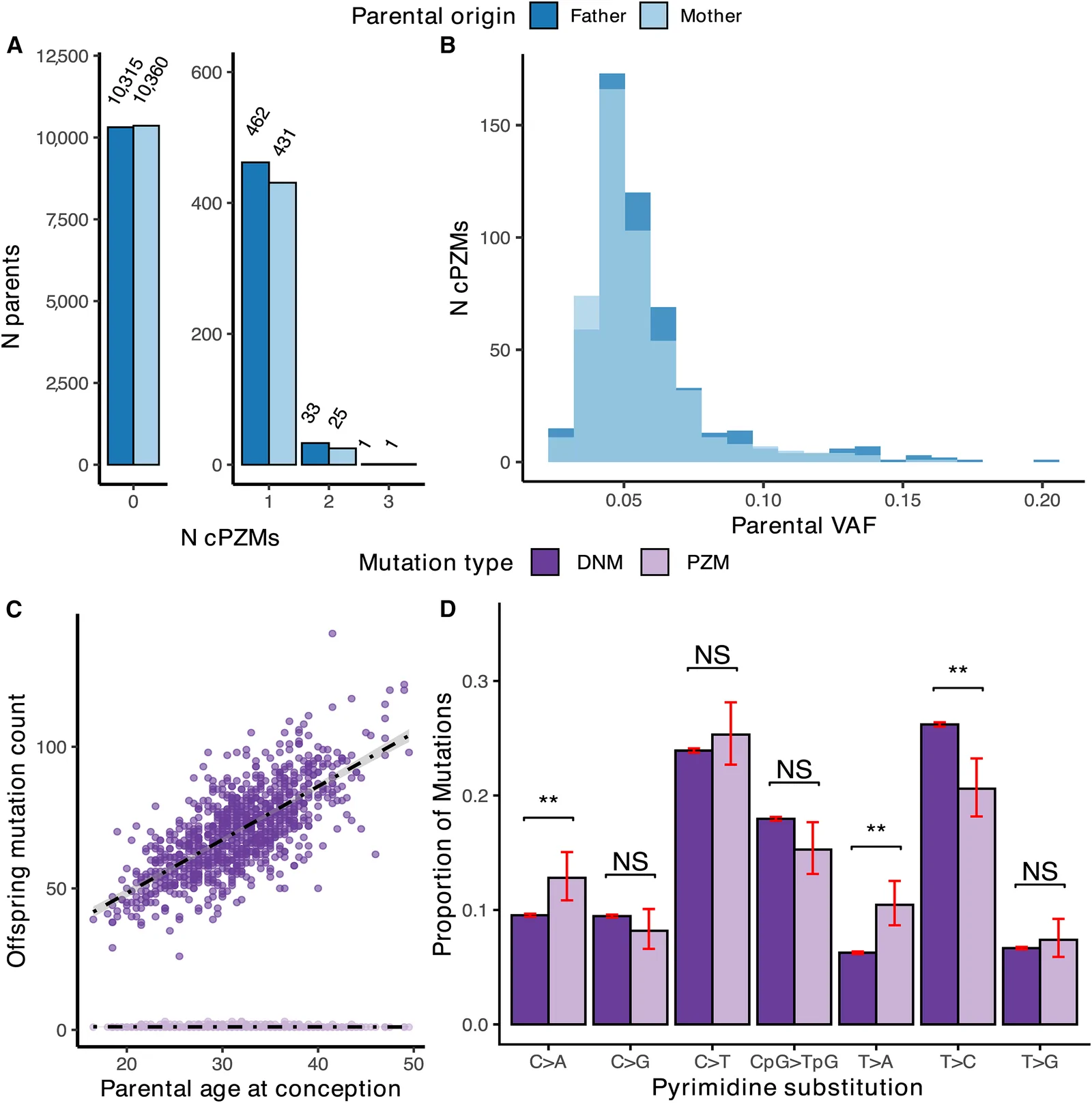
Characteristics of parental postzygotic mutations (A) Distribution of PZM burden (number per individual) across the parental cohort (*n* = 21,650). (B) Distribution of PZM variant allele fraction (VAF). No significant maternal vs. paternal differences in burden or VAF (Wilcoxon rank-sum *p* = 0.19 and 0.09, respectively). (C) Effect of mean parental age at conception on offspring PZMs (light purple) and DNMs (dark purple). There was no significant age effect for PZMs (generalized linear regression *p* = 0.98). (D) Mutation spectra comparison between PZMs and DNMs. PZMs were significantly enriched (FDR ≤ 5%) for C>A and T>A mutations (Fisher’s exact test, adjusted *p* = 5.3 × 10^−3^ and 2.1 × 10^−5^, respectively) and depleted for T>C (Fisher’s exact test, adjusted *p* = 4.5 × 10^−3^) compared to DNMs. Red bars indicate 95% confidence intervals for 1-sample proportion test per pyrimidine proportion.

Across all cPZMs, we observed a monomodal VAF distribution centered on 0.05 (±2.77 × 10^−3^, 95% confidence interval [CI]), with no significant difference between parental sexes (Wilcoxon rank-sum test *p* = 0.09; Figure 2B). Under a simplified model assuming synchronous cell divisions, stable diploidy, no selection, and uniform contribution of mutant cells across adult tissues, this is consistent with mutations arising around the third embryonic cell division (Table S1). The observed distribution reflects both this developmental window and the technical boundaries of our ascertainment, which impose lower and upper detection limits. At the lower end, limited sensitivity at standard sequencing depth restricts detection to events occurring before approximately the 5th division (VAF∼0.02; Table S1), well before PGC specification (∼10th–15th division). At the upper end, detection is limited both by our deliberate exclusion of high-VAF variants to avoid germline heterozygotes and by the tendency of standard germline callers to call intermediate-to-high VAF mosaic variants as constitutive heterozygotes, excluding them at the Mendelian inconsistency stage. In our cohort, parental variants at VAFs ≥ 0.14 had an empirically estimated ≥5% probability of being called heterozygous by Platypus, rising to 50% at VAFs ≥ 0.17 (supplemental material and methods; Figure S10). Our catalog is therefore most informative for PZMs that arise in the intermediate developmental window between these boundaries.

Having established the detection boundaries of our approach, we first confirmed that our PZM catalog exhibits expected biological properties before characterizing features not previously assessed at the genome-wide scale.

The parental age effect on DNM rate is well established and has been proposed to result from the interplay between DNA damage and repair throughout aging.^23^ Since PZMs arise during early development, we do not expect them to be associated with parental age. To test this, we aggregated PZM counts across both parents for each offspring and modeled the total as a function of maternal and paternal age at conception, adjusting for technical covariates (supplemental material and methods; Figure 2C). As expected, neither maternal nor paternal age showed a significant effect on PZM counts (*p* = 0.5 and 0.42, respectively). In contrast, DNM counts were significantly and positively associated with both maternal age (effect = 7.2 × 10^−3^, *p* = 6.37 × 10^−14^) and paternal age (effect = 1.8 × 10^−2^, *p* < 2 × 10^−16^). These results support the specificity of our PZM calls and suggest that they are not confounded by misclassified DNMs or parental-age-related somatic variants.

Consistent with the absence of a parental age effect and in line with previous observations in smaller cohorts,^1^ we found no significant difference in PZM counts between parental sexes (Wilcoxon rank-sum test *p* = 0.19; Figure 2A), with 52.3% of events identified in fathers. The scale of our catalog allowed us to test this with greater statistical power than has previously been possible, lending further support to the hypothesis that the opportunity to acquire early PZMs is equivalent across sexes in humans. This contrasts with mice, where a paternal bias has been attributed to earlier PGC specification in males,^24^ suggesting that these sex-specific developmental mechanisms do not operate equivalently in humans.

We next investigated the mutational spectra of PZMs to characterize the mutagenic processes underlying early developmental mutations. We classified mutations according to six substitution types (C>A, C>T, C>G, T>A, T>C, and T>G) based on the pyrimidine substitution caused by each single point mutation,^18^ additionally separating CpG>TpG transitions to account for spontaneous cytosine deamination^1^^,^^25^ (supplemental material and methods). Comparing substitution-type proportions between paternal and maternal PZMs revealed no significant differences (two-sample proportion test, false discovery rate [FDR]-adjusted *p* > 0.05; Figure S11), extending the sex equivalence observed in mutation counts to the underlying mutational processes.

Comparing PZM and DNM spectra, we identified three substitution classes with significant differences in relative frequency (Fisher’s exact test, FDR-adjusted *p* ≤ 0.05; Figure 2D). PZMs were significantly enriched for C>A and T>A mutations relative to DNMs (PZM/DNM odds ratio [OR] = 1.34; 1.66, adjusted *p* = 5.3 × 10^−3^ and 2.1 × 10^−5^, respectively) and significantly depleted in T>C mutations (PZM/DNM OR = 0.78; adjusted *p* = 4.5 × 10^−3^). To contextualize these findings, we compared our spectral profile against developmental-window-stratified PZM subsets from Sasani et al.^16^ and Jonsson et al.,^17^ accounting for differences in VAF distributions introduced by their extended family designs (supplemental material and methods; Figure S12; Table S2). Our C>A enrichment and T>C depletion relative to DNMs are consistent with significant patterns observed in PZM subsets enriched for events arising around or after PGC specification.^16^^,^^17^ In contrast, the T>A enrichment we observe aligns with a PZM subset capturing pre-PGC events at higher parental VAFs (median = 0.241).^16^ Although our catalog is centered on a narrow, low-VAF window (median = 0.049), its spectral profile does not correspond exclusively to a single developmental stage, instead reflecting a mixture of mutational signatures associated with distinct developmental windows across independent studies. This indicates that despite our ascertainment constraints, the catalog retains sufficient representation across postzygotic developmental timings to capture reproducible spectral features of postzygotic mutagenesis.

Having validated our catalog through substitution-level spectral comparisons, we next characterized the genomic distribution and mutational processes underlying early PZMs.

We investigated whether PZMs exhibit non-random genomic distributions by partitioning the genome into 2 Mb bins and testing for significant PZM excess relative to a uniform null model (supplemental material and methods). We identified 49 nominally enriched bins (one-tailed empirical *p* ≤ 0.05), none of which survived multiple testing correction (FDR >5%; Figure S13). With this caveat in mind, we asked whether the nominally enriched bins were associated with GC content or replication timing (supplemental material and methods). Using binomial regression while adjusting for callable size, we found that GC content was negatively associated with PZM enrichment (OR = 0.87, *p* = 0.016), whereas replication timing showed no significant effect (*p* = 0.51). Applying the same approach to DNM-enriched bins recapitulated the established positive association with GC content and negative association with replication timing,^26^^,^^27^ supporting the robustness of our method (supplemental material and methods). The negative GC content association for PZMs contrasts with the well-documented positive association for germline DNMs, which is largely driven by spontaneous deamination of methylated cytosines at CpG sites enriched in GC-rich regions.^26^ One possible explanation is that the extensive DNA demethylation that occurs during post-fertilization epigenetic reprogramming^28^ attenuates this source of mutagenesis in GC-rich regions during the cleavage divisions in which PZMs arise, though this interpretation should be considered speculative given the exploratory nature of our analysis.

To identify the mutational processes driving the observed spectral differences between PZMs and DNMs, we performed mutational signature extraction and deconvolution (supplemental material and methods). Both mutation types were predominantly shaped by SBS1 and SBS5^29^ (Figure S14), two clock-like mutational signatures whose effects are widespread across somatic^18^^,^^30^ and germline^1^ tissues. SBS1 is dominated by cytosine transitions in the CpG context, and its accumulation rate increases with cellular turnover. SBS5 has a more diffuse distribution across mutation types and is thought to reflect background endogenous cellular damage to DNA.^23^ Because the relative contributions of these two processes are known to vary across developmental contexts and cell types,^31^ we compared the proportion of mutations attributed to SBS1 and SBS5 between PZMs and DNMs. However, we found no significant difference in the proportion of mutations attributed to SBS1 or SBS5 between PZMs and DNMs (two-sample proportion test, *p* = 0.39 and 0.47, respectively), indicating that the same mutational processes underlie both mutation types in broadly similar proportions. The C>A, T>A, and T>C differences observed at the substitution level may therefore reflect subtle context-dependent shifts in the activity of these shared processes across distinct developmental environments rather than entirely distinct mutational origins.

Finally, we assessed the functional distribution of PZMs. Consistent with the sequence composition of the genome, *in silico* consequence prediction^32^ showed that 96.6% of PZMs occurred in non-exonic locations (Figure S15). We tested whether PZMs were preferentially located in specific functional contexts, including transcriptional units (introns, exons, and UTRs), putative regulatory regions (5,000 bases upstream of transcription start sites or downstream of transcription termination sites), and intergenic regions (supplemental material and methods), but found no greater-than-expected overlap with any tested category. PZM VAFs also did not differ significantly between consequence classes, suggesting that functional context does not detectably influence the retention or detection of early PZMs.

A primary goal of GEL is to identify disease-causing genetic variations in individuals suffering from rare diseases.^21^ This involves evaluating pathogenic and likely pathogenic protein-coding variants that either arise *de novo* in the affected offspring or are inherited as heterozygous from an affected parent and that fall within phenotype-relevant genes curated in PanelApp.^20^^,^^33^ As parental PZMs fall outside such conventional inheritance classifications, the 1,015 variants identified here fall into two groups. The majority (*n* = 917) were not called by standard variant-calling pipelines in either parent or child and therefore represent novel variation not previously evaluated for clinical relevance. The remaining 98 correspond to parental PZMs with extremely low VAFs, typically supported by only two reads, which were initially misclassified as DNMs.

Although these 98 variants entered clinical interpretation workflows, their misclassification as DNMs leads to underestimation of recurrence risk when the variant is pathogenic. Among them, we identified a clinically relevant missense variant in *VAMP2* (MIM: 185881), a gene associated with neurodevelopmental disorders,^34^ previously reported as the diagnostic variant in an individual with specific learning disability and global developmental delay (Tables 1 and S3). Although in this instance the finding did not alter clinical management because the mosaic carrier’s parents were beyond reproductive age, it illustrates how parental PZMs misclassified as DNMs can carry recurrence implications that are missed under standard interpretation.

**Table 1:** Summary of clinically relevant parental PZMs in the GEL cohort

**RefSeq HGVS variant IDs**	**Gene symbol (OMIM gene ID)**	**Panels (PanelApp) applied by clinical team**	**Reclassed DNM**	**Parental VAF (alternative allele/total reads)**	**Missense predictions**	**Phenotype**	**ClinVar status**	**ACMG class prediction (ACMG codes)**	**Clinician assessment**
g.8161693C>G (GenBank: NC_000017.11); c.197G>C (GenBank: NM_014232.3); p.Arg66Pro (GenBank: NP_055047.2)	*VAMP2* (MIM: 185881)	intellectual disability	yes	3.08% (2/65)	AM = 0.99; CADD = 33; CP = 0.99	specific learning disability; global developmental delay	pathogenic	pathogenic (PM2, PS1, PP2, PP3, PS2)	variant already reported as a pathogenic DNM in this individual
g.101985930C>T (GenBank: NC_000014.9); c.1705C>T (GenBank: NM_001376.5); p.Arg569Trp (GenBank: NP_001367.2)	*DYNC1H1* (MIM: 600112)	malformations of cortical development; early onset or syndromic epilepsy	no	5.26% (2/38)	AM = 0.99; CADD = 24.8; CP = 0.97	intellectual disability; bilateral tonic-clonic seizure	pathogenic for different phenotype; no entry for relevant phenotype	likely pathogenic (PM2, PS1, PP2, PS2)	not available for this individual; same variant occurred as DNM in another GEL individual in whom it was determined to be pathogenic
g.32389172C>A (GenBank: NC_000011.10); c.1455G>T (GenBank: NM_024426.6); p.Lys485Asn (GenBank: NP_077744.4)	*WT1* (MIM: 607102)	proteinuric renal disease; unexplained kidney failure in young people	no	3.33% (2/60)	AM = N/A; CADD = 23.4; CP = 0.99	hydroureter; vesicoureteral reflux; proteinuria	not in ClinVar	VUS (PM2, PP2, PS2)	variant considered clinically reportable after review by clinician

A description of the codes used for the ACMG class assignment is provided in Table S3. MCD, malformations of cortical development; AM, AlphaMissense; CP, ClinPred; N/A, not available.

For the 917 previously untested PZMs, we intersected genes carrying protein-coding variants with a VEP “MODERATE” or “HIGH” consequence against PanelApp green genes, identifying four additional variants in genes with established clinical relevance: *WT1*, *DYNC1H1*, *PHIP*, and *NLRP3* (MIM: 607102, 600112, 612870, and 606416, respectively). For each, we reviewed the proband’s diagnosis status and recorded phenotypes against gene-disease associations documented in PanelApp and the literature. The proband carrying the *NLRP3* variant had already received a diagnosis based on another pathogenic variant, and the *PHIP* variant carrier showed only a limited phenotypic match for this gene (supplemental material and methods). The remaining two variants, in *WT1* and *DYNC1H1*, were considered potentially clinically relevant for the previously undiagnosed offspring in each trio and reported back to the individuals’ clinicians. In both cases, the variant was present as a somatic mosaic in an unaffected parent and transmitted to the offspring as a constitutive heterozygous variant (Tables 1 and S3).

The missense variant in *DYNC1H1* affects the canonical transcript (ENST00000360184.10, exon 8/78), is absent from gnomAD, and was classified as likely pathogenic by ClinPred and AlphaMissense and mildly pathogenic by CADD (scores = 0.97, 0.99, and 24.8, respectively). *DYNC1H1* is associated with several neuromuscular and neurodevelopmental disorders (MIM: 614228, 614563, and 158600), including seizure disorders.^35^^,^^36^ Inspection of the GEL variant tiering database revealed that the same variant had been independently identified as a germline DNM in an unrelated GEL participant with seizures and had also been previously reported in the literature in an individual with a *de novo* occurrence of the variant and a seizure disorder.^37^

The missense variant in *WT1* affects the canonical transcript (ENST00000452863.10, exon 10/10), absent from gnomAD and predicted to be deleterious by CADD and ClinPred (scores = 23.4 and 0.99, respectively). Mutations in *WT1* are known to cause Wilms tumor (MIM: 194070) and gonadal and urinary tract malformations (Denys-Drash syndrome, MIM: 194080).^38^ In this trio, the variant was present as a postzygotic mosaic mutation in the parent and transmitted to the offspring, who showed congenital anomalies of the kidney and urinary tract (CAKUTs), including hydroureter, vesicoureteral reflux, and proteinuria. Although *WT1* was not included in the diagnostic panel originally applied to this individual, both vesicoureteral reflux and proteinuria are recognized phenotypic features of pathogenic *WT1* variants,^39^^,^^40^ and the recruiting clinician considered the variant clinically reportable for both individual clinical management and familial risk assessment. These examples illustrate the potential diagnostic value of systematic parental PZM ascertainment in recovering clinically relevant variants that would otherwise escape routine pipelines.

In conclusion, we developed a bioinformatic annotation pipeline to detect early parental mosaicism from standard-depth trio WGS. Although detection across the VAF spectrum is constrained by the boundaries imposed by standard genotype calling in the trio setting, we demonstrate that our annotation criteria enable recovery of true PZM events and provide an empirical derivation of these boundaries that can inform future efforts to identify PZMs from comparable sequencing data. Since this approach does not require specialized sequencing or extended family designs, it permitted population-scale characterization of early PZMs. While most spectral features detected are consistent with findings from smaller studies, the scale of our cohort enabled analyses, including mutational signature extraction and exploratory genome-wide distribution testing, suggesting that PZMs may be depleted in GC-rich regions, contrasting the well-documented positive association for germline DNMs. Although our method yields a lower per-family yield than approaches employing deep or long-read sequencing, it requires no additional data beyond standard clinical WGS, making it readily deployable as a lightweight annotation step in both routine trio analysis and reanalysis of existing WGS datasets. Parental PZMs, though infrequent, can cause rare conditions with direct implications for recurrence risk, and as most remain undetected by current clinical pipelines, systematic screening at scale is needed to improve diagnostic yield and outcomes for affected families.

## Data and code availability

There are restrictions on the availability of the catalog of PZMs described in this manuscript due to GEL policies on the export of individual-level information. This catalog can be accessed from the GEL research environment upon request to join the GEL research network at https://www.genomicsengland.co.uk/join-us/academics. In the GEL research environment, our catalog of PZMs and its associated code can be found in the following path: /re_gecip/shared_allGeCIPs/parental_pzm_cat.

## Acknowledgments

We thank the Wellcome Trust for funding this research. R.R. and H.C.M. were supported by the Wellcome grant 220540/Z/20/A. This research was made possible through access to data in the National Genomic Research Library, which is managed by Genomics England Limited (a wholly owned company of the Department of Health and Social Care). The National Genomic Research Library holds data provided by patients and collected by the NHS as part of their care, as well as data collected as part of their participation in research. The National Genomic Research Library is funded by the National Institute for Health Research and NHS England. We also thank Graciella Martin Rijo for generously sharing her views and suggestions on data visualization. For the purpose of open access, the authors have applied a CC-BY public copyright license to any author-accepted manuscript version arising from this submission.

## Author contributions

O.I.G.-S. conducted the analyses and drafted the manuscript; R.S., M.T.I.B., and M.H.P. advised on QC or specific analyses and contributed to the generation of figures; K.A.A. and J.A.S. provided clinical interpretation; A.S. provided scientific advice; and H.C.M. and R.R. jointly supervised the study.

## Declaration of interests

The authors declare no competing interests.

## Web resources

GenBank, https://www.ncbi.nlm.nih.gov/genbank/.
OMIM, https://www.omim.org.

## Supplemental information

Document S1. Figures S1–S15, Tables S1–S3, and supplemental material and methods

Document S2. Article plus supplemental information
