# Supplementary material for: Landscape of parental postzygotic mutations across >11,000 rare disease trios
Source: Am J Hum Genet. 2026 Jul 13;113(8):1762–70. doi: 10.1016/j.ajhg.2026.06.015 (PMC13504344; doi:10.1016/j.ajhg.2026.06.015)
Supplement: Supplementary file 1 [file mmc1.pdf]

The American Journal of Human Genetics, Volume *113*

Supplemental information

Landscape of parental postzygotic mutations across >11,000 rare disease trios

O. Isaac Garcia-Salinas, Katrina A. Andrews, Rashesh Sanghvi, John A. Sayer, Maria Torra I Benach, My H. Pham, Aylwyn Scally, Hilary C. Martin, and Raheleh Rahbari

Supplemental figures

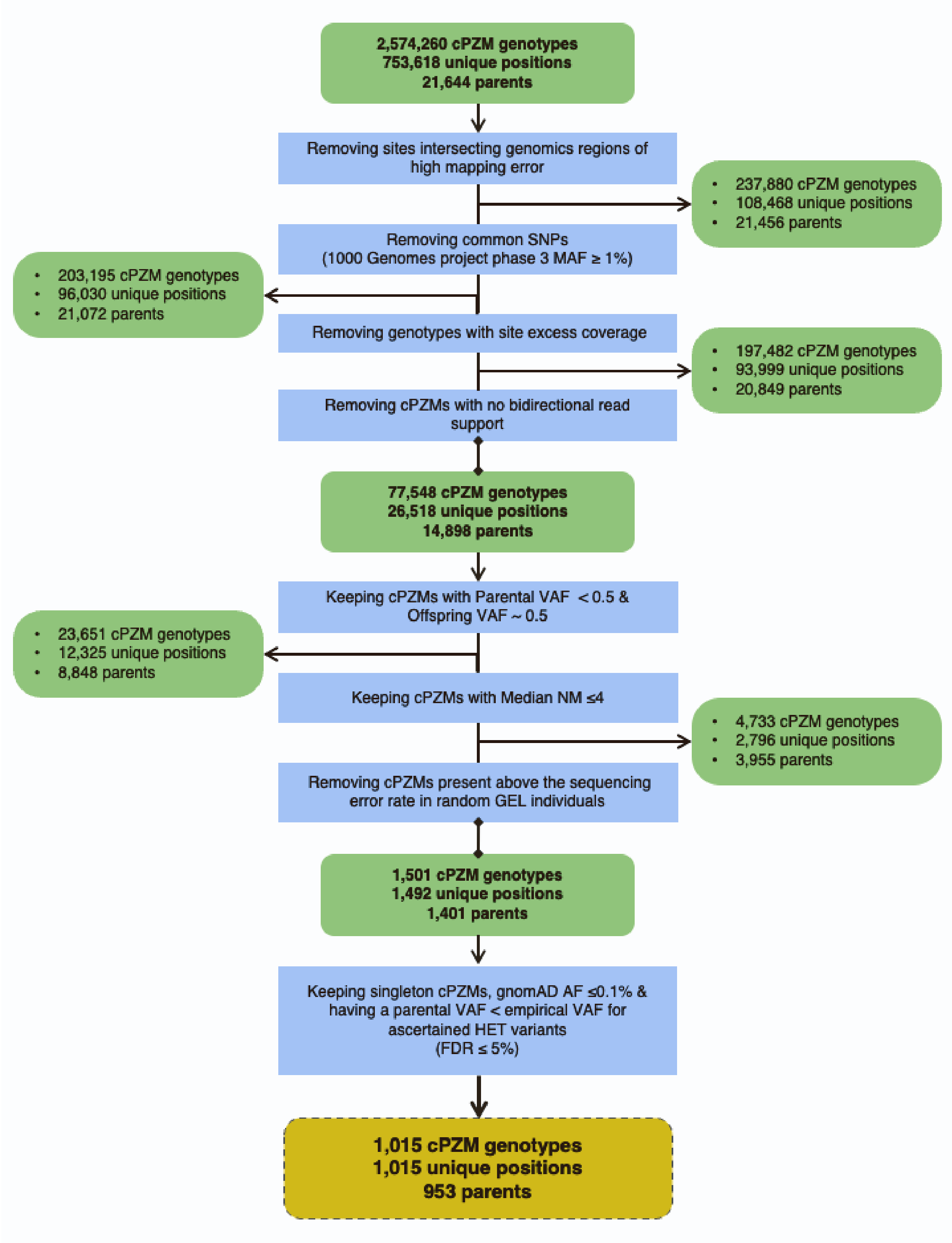

Figure S1. Summary of filtering steps for bioinformatic ascertainment of parental postzygotic mutation in the GEL family trio cohort.

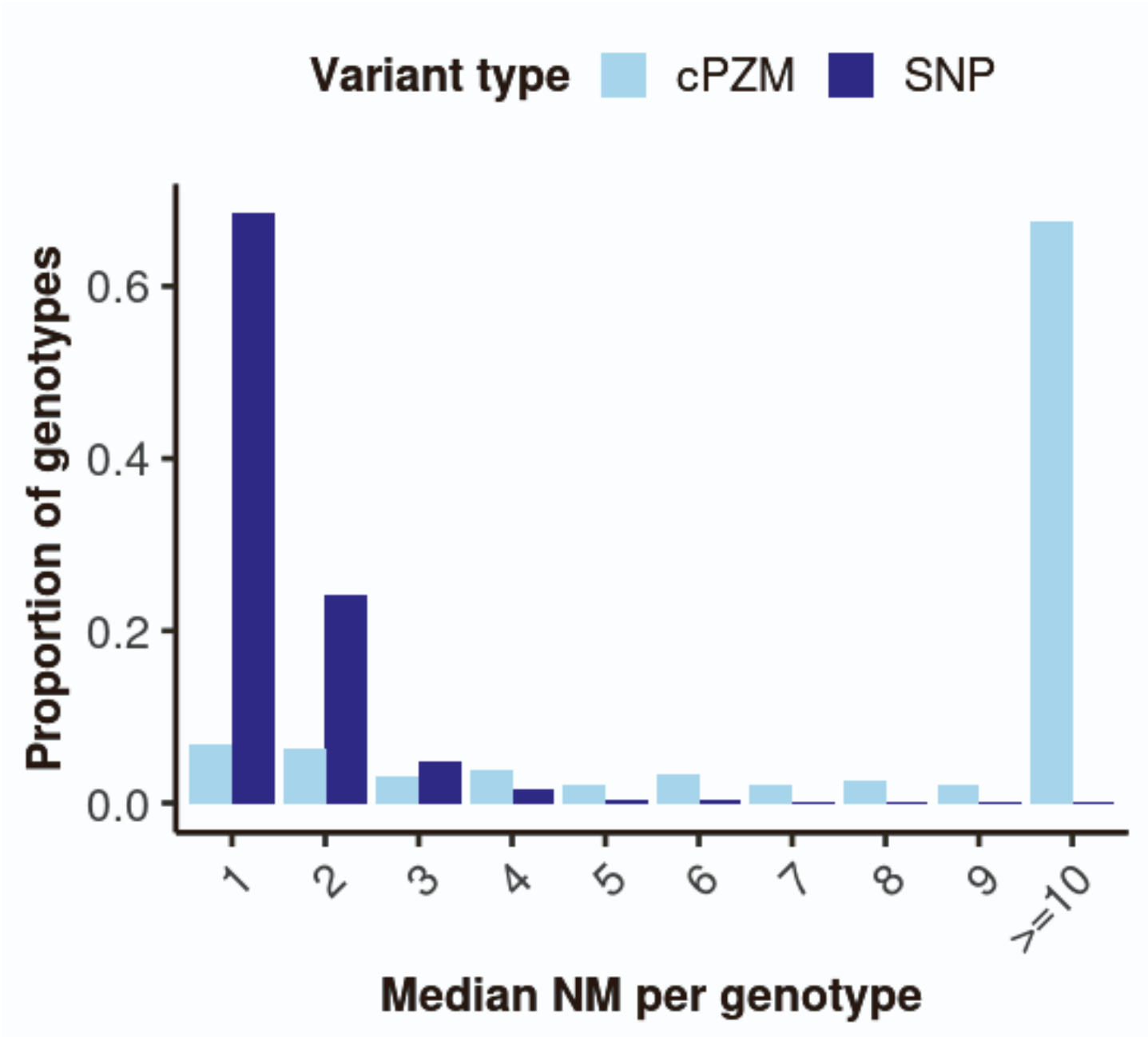

Figure S2. Distribution of median number of mismatches (NM) for reads mapping to cPZMs and GIAB SNPs. Showing proportions out of a total of 23,651 cPZMs passing filtering steps 1-5, and up to 4600 high quality SNPs.

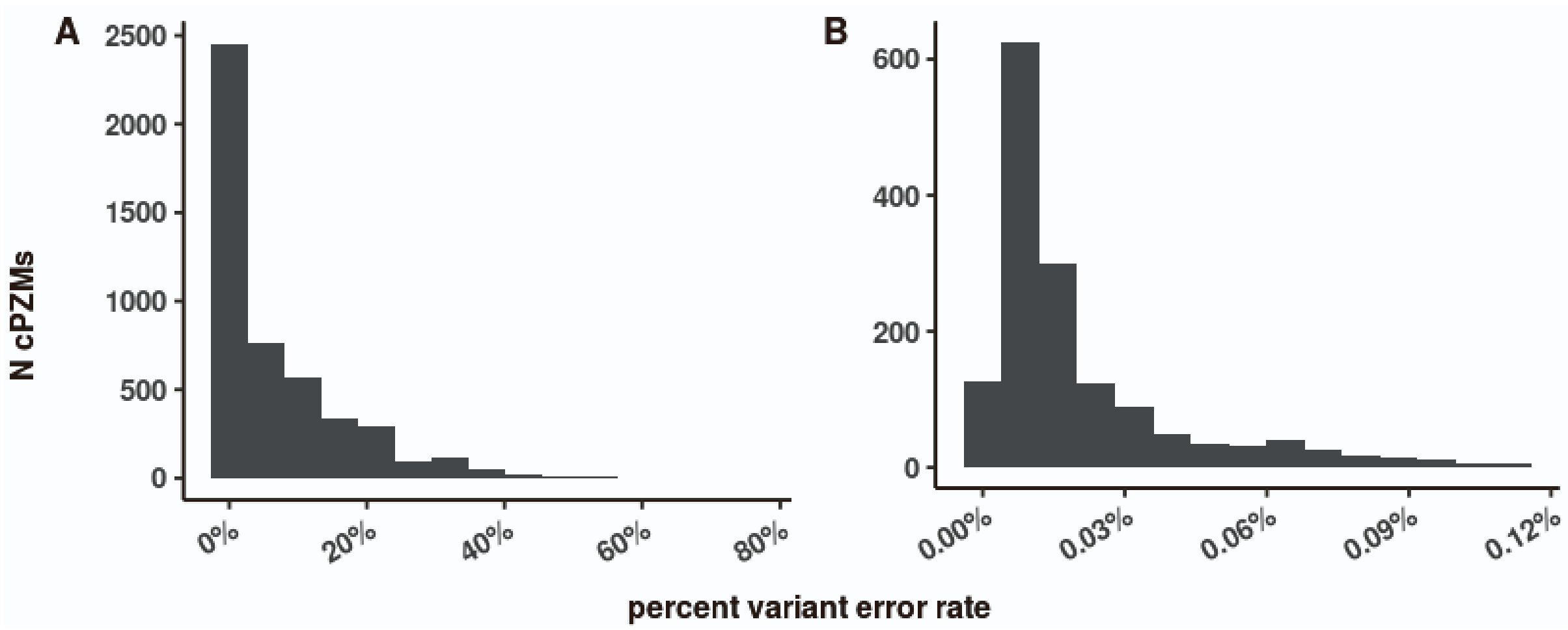

Figure S3. Variant-specific error rate distributions for cPZMs passing filtering steps 1-6. Showing variant specific error rates for 4,734 cPZMs **A.** passing filtering steps 1-6 **B.** passing filtering step 8 (n = 1,501). Error rate filtering threshold corresponds to the median Illumina HiSeq 2500 error rate (0.112%).

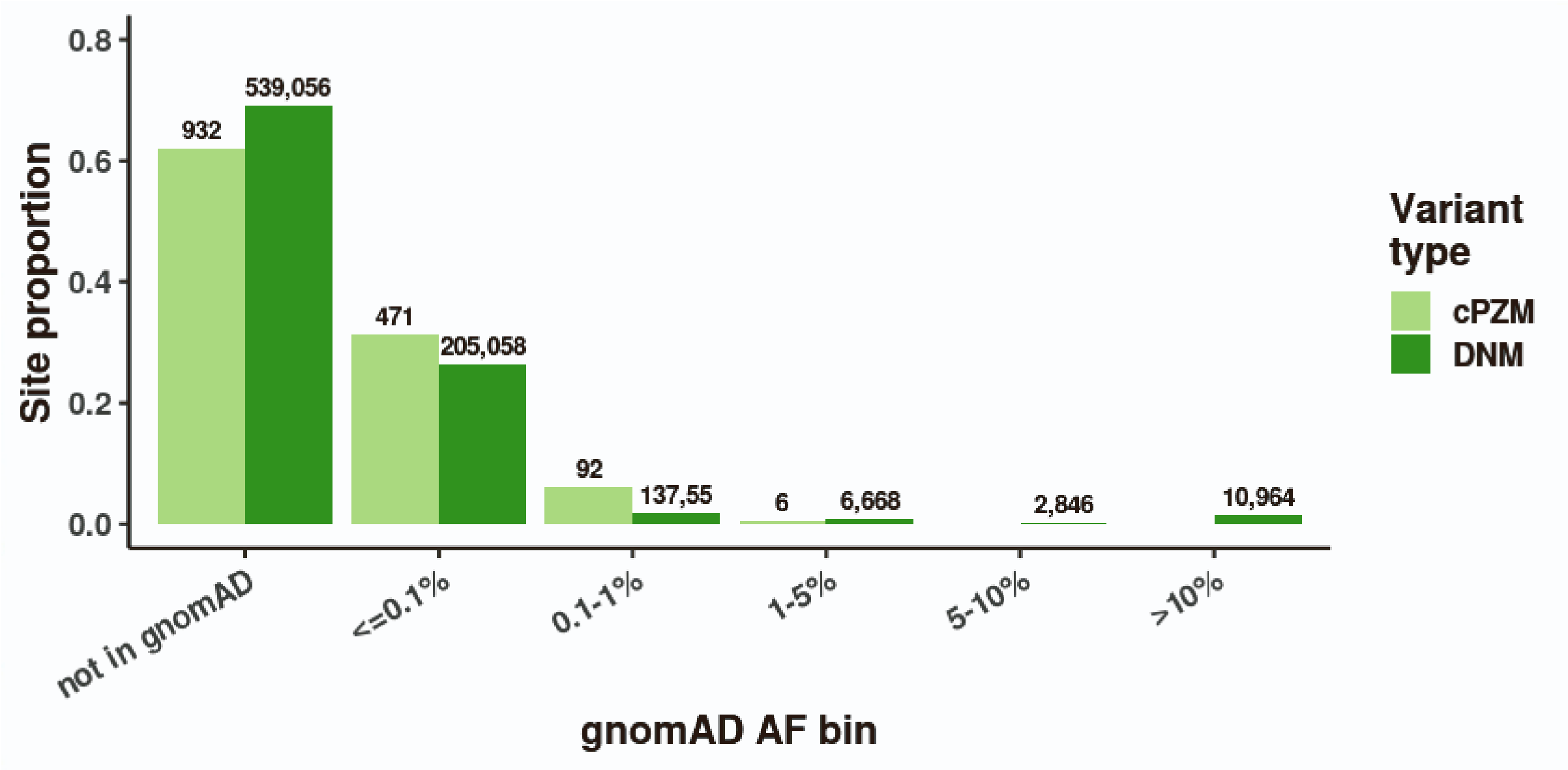

Figure S4. Distribution of population allele frequencies (gnomAD genomes v3). Showing binned gnomAD allele frequency distributions for 1,501 cPZMs remaining after step 1-7 filtering and filtered GEL DNMs per trio (stringent filter) reported by Kaplanis et al. [3]. Top numbers indicate counts per bin and variant type.

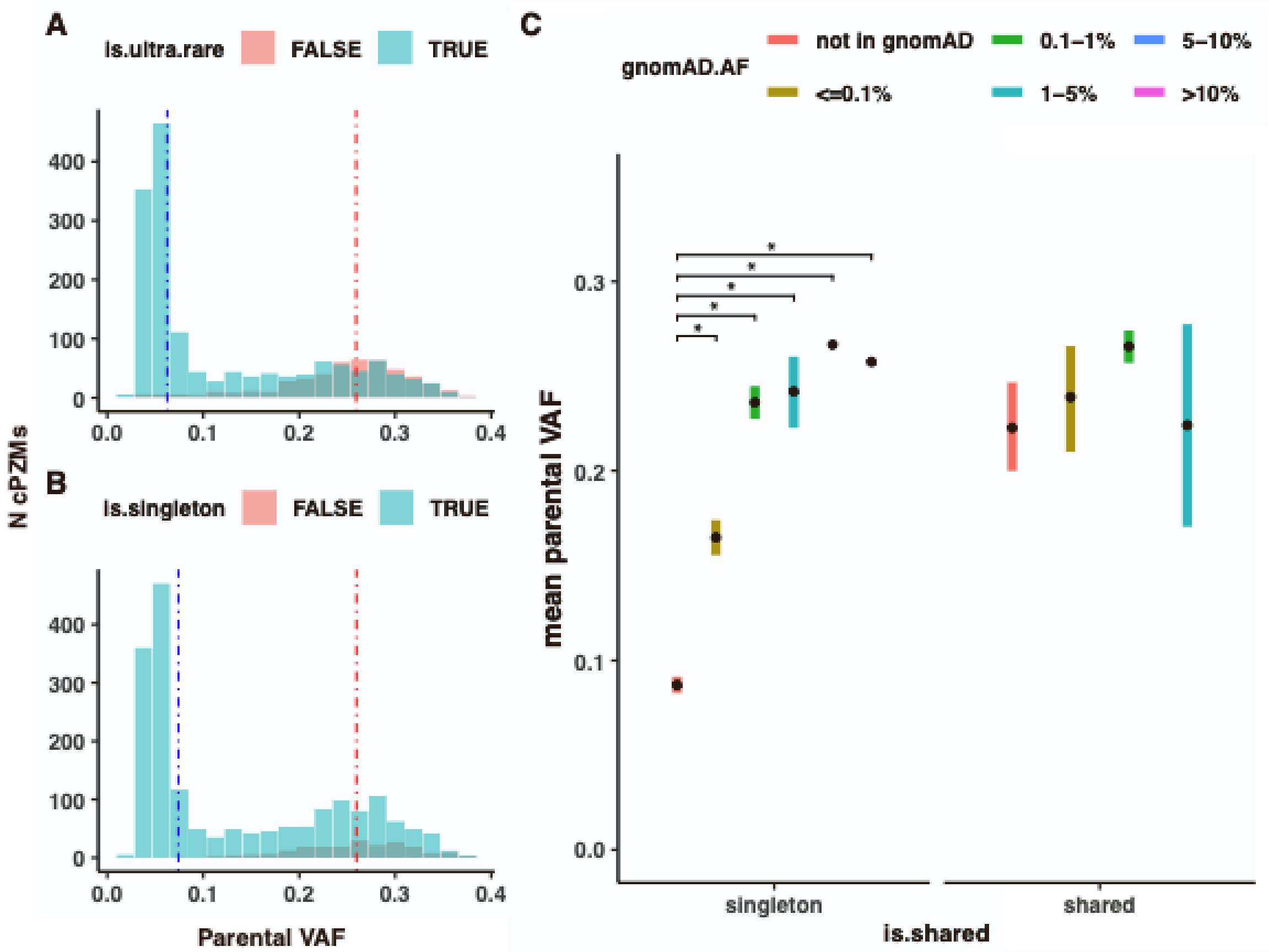

Figure S5. Parental VAF comparisons across population allele frequency bins for cPZMs passing filtering steps 1-7. A. Parental VAF of ultra-rare and non-ultra-rare cPZMs classes (n = 1,501). The ultra-rare group represents a composite between cPZMs absent from gnomAD or having a gnomAD AF ≤0.1%. **B.** Parental VAF of cPZMs found as singletons (i.e., found in a single parent) or shared across the 1,401 unrelated parents having at least one cPZM passing filtering steps 1-7. **C.** Mean parental VAFs for cPZMs stratified into six population allele frequency bins (gnomAD) and according to their presence across unrelated parents (i.e. singleton = seen in only one parent, shared = seen in one or more parent). Bars correspond to 95% confidence intervals for the mean estimate for each group. Horizontal lines and asterisks indicate significant VAF differences between groups (ANOVA and Tukey HSD post-hoc test adjusted p-values ≤ 0.05).

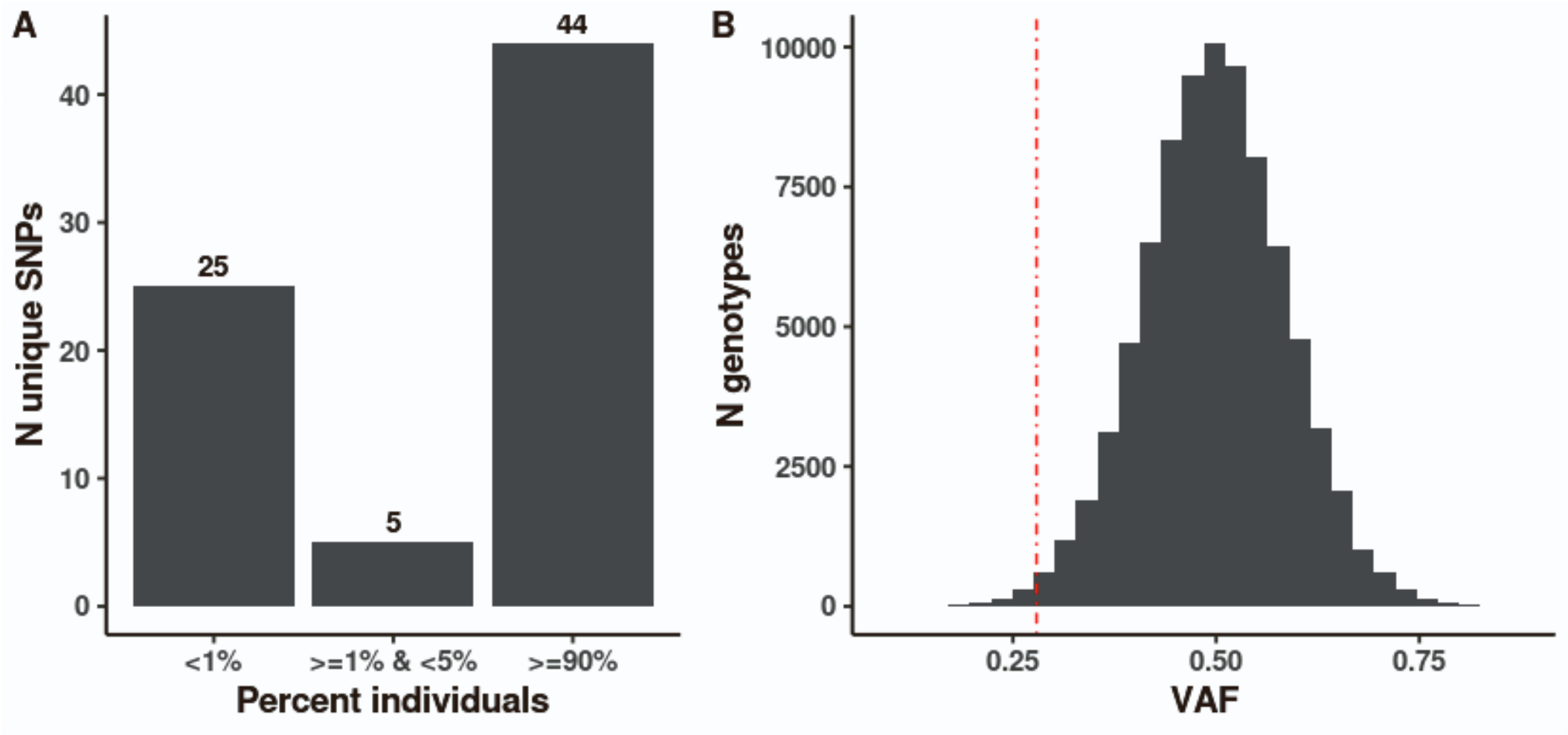

Figure S6. Description of SNPs selected to derive an empirical lower variant allele fraction (VAF) bound threshold for heterozygous (HET) variants. A. Number of HET SNPs shared across different proportions of the subcohort of interest (n = 1,293)**.** Top numbers indicate the number of individuals in each bar. **B.** VAF distribution for HET genotypes at the selected SNPs. The red line overlaps the mean lower-bound VAF threshold (mean VAF threshold = 0.27) across 1,293 individuals.

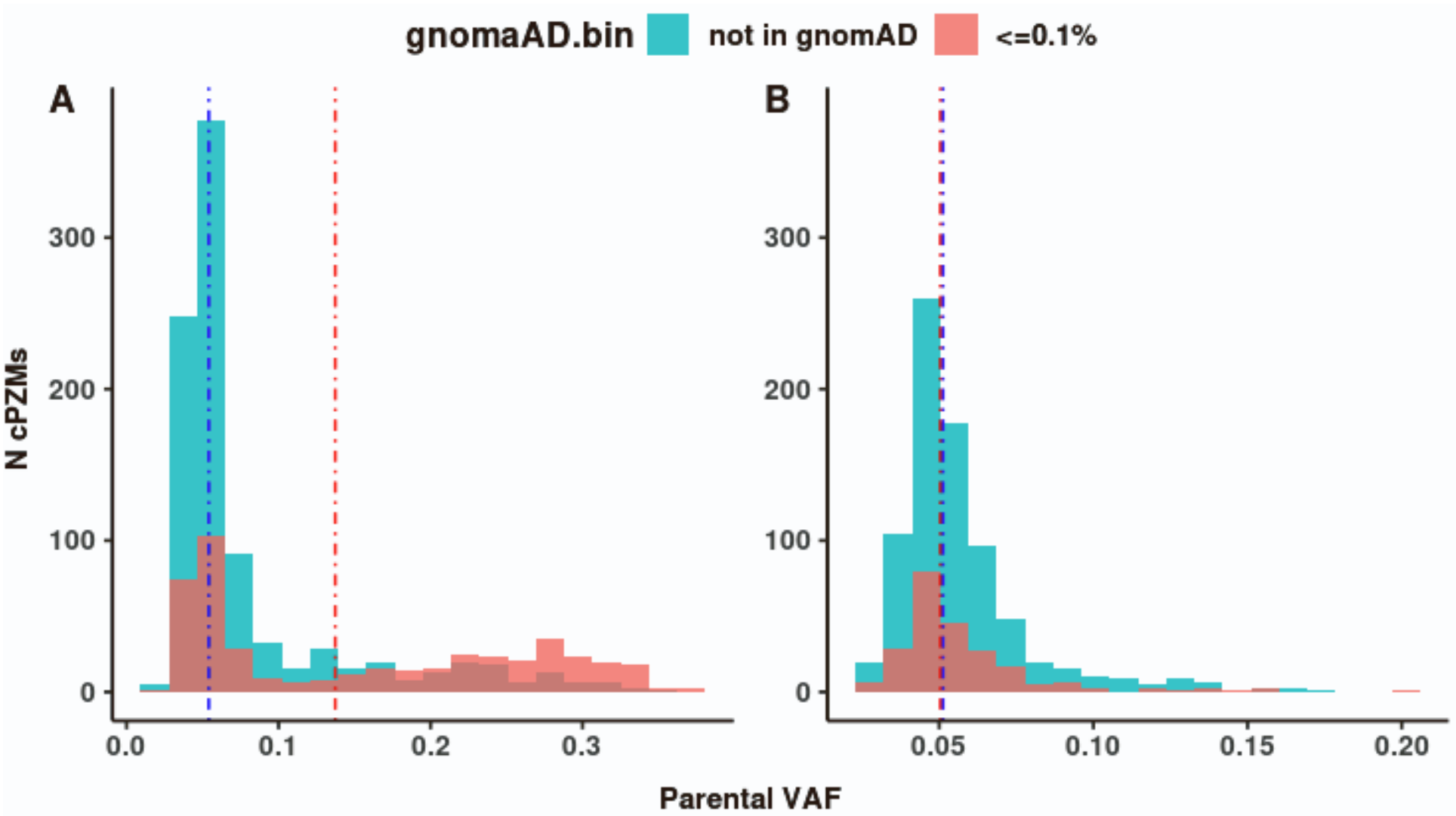

Figure S7. Parental VAF distribution comparisons for singleton cPZMs stratified by gnomAD allele frequency. A. All singleton cPZMs passing filtering steps 1-8 (n = 1,389). **B.** Singleton cPZMs after removing putative HET sites (filtering step 9; n = 1,015). Dotted lines intersect with median parental VAFs for cPZMs not found in gnomAD (blue), and found in gnomAD (red) in each panel. Median parental VAF is significantly different between groups before removing putative HETs (Wilcoxon rank-sum test p-value = 2.2×10^{-16}), and non-significant after filtering these sites (Wilcoxon rank-sum test p-value = 0.98).

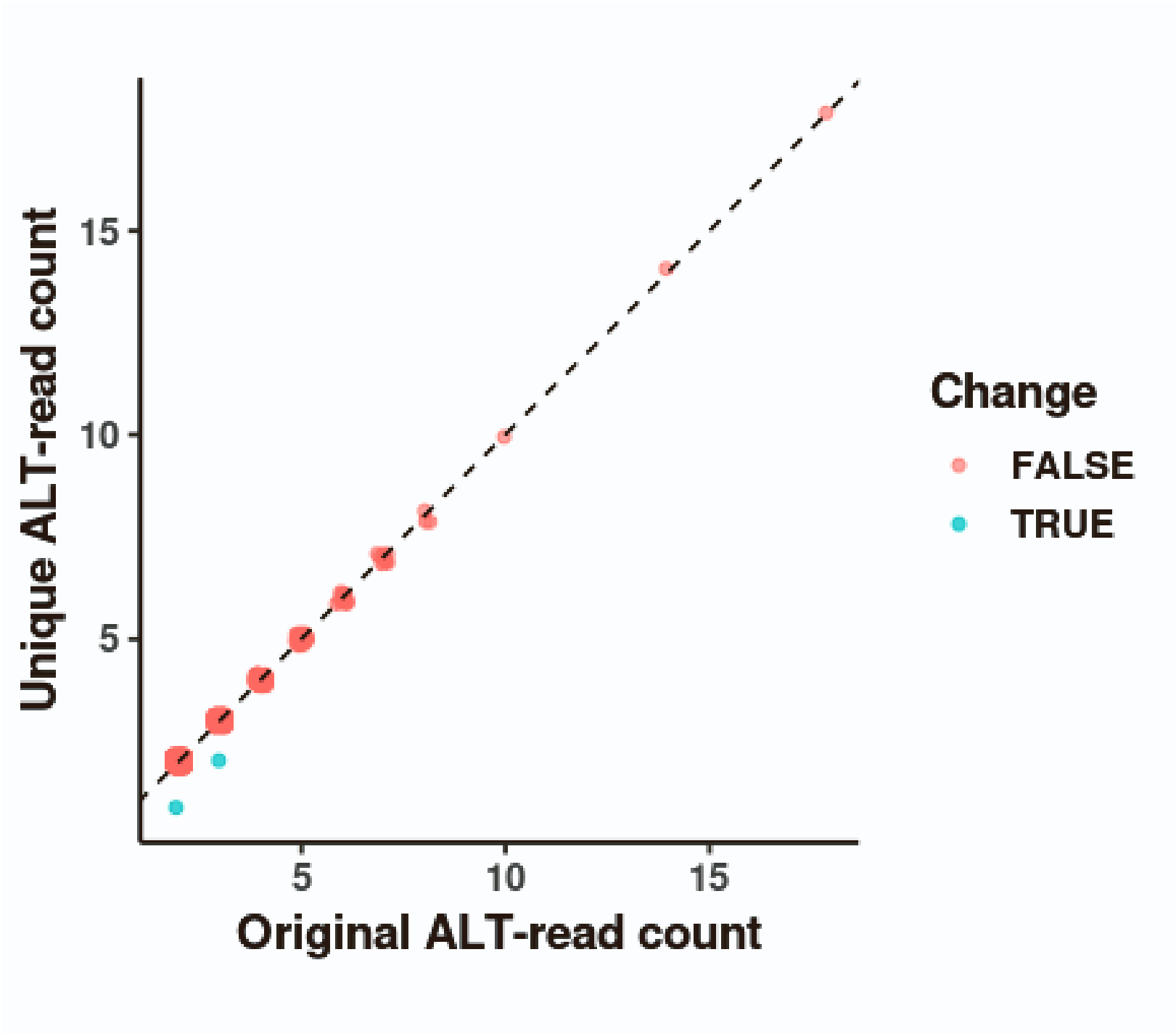

Figure S8. Sanity check for unique alternative allele read support for final PZM candidates. Showing the number of reads supporting the alternative (ALT) allele for all final PZM candidates (n = 1,015). For each PZM, the x-axis shows the alternative allele read support before subsetting to unique read names, while the y-axis shows the remaining read counts after keeping unique read names. Colors indicate whether there was reduction in the total ALT supporting reads after keeping unique read names.

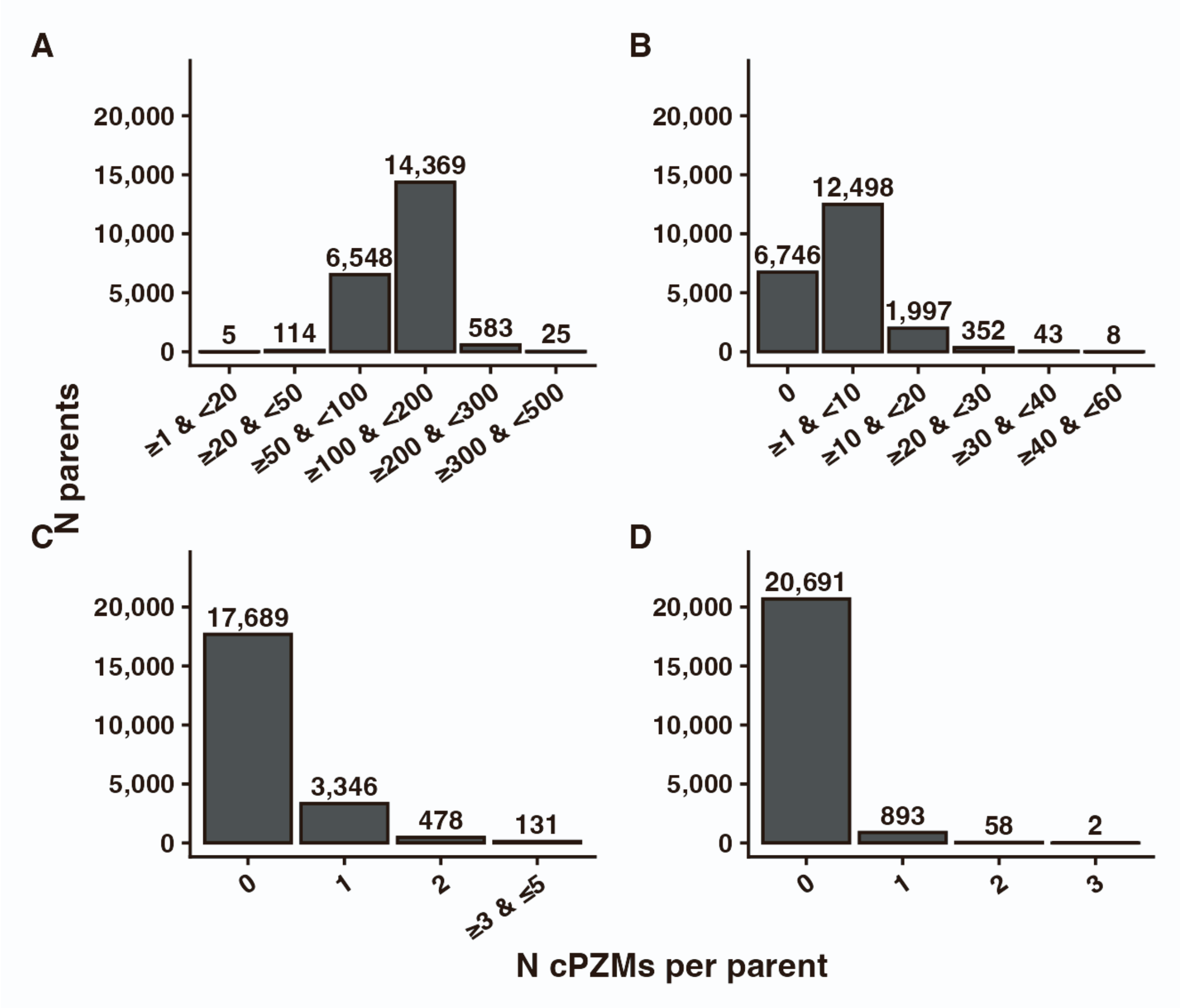

Figure S9. Number of cPZMs per parent across main filtering steps. Number of candidate cPZMs per individual across the whole initial cohort of 21,644 parents for: **A.** Initial selection of 2,574,260 cPZMs. **B.** 77,548 cPZMs remaining after removing variants intersecting genomic regions of high mapping error, reported in the 1kGP phase 3 (MAF ≥1%)[5], with a total site sequencing above individual sequencing-excess thresholds (as defined in filtering step 2 - **"**Excess coverage filter"), or supported by less than one read on each strand **C.** 1,501 cPZMs remaining after removing likely HET variants based on parental and offspring VAFs, having a median NM >4, or being above the median Illumina 2500 error rate in random GEL participants. **D.** 1,015 cPZMs remaining after removing variants with gnomAD AF >0.1%, shared across unrelated parents, or not being significantly different from the empirical lower-bound VAF threshold for ascertained HET variants (FDR >5%). Top numbers indicate counts per bin and variant type.

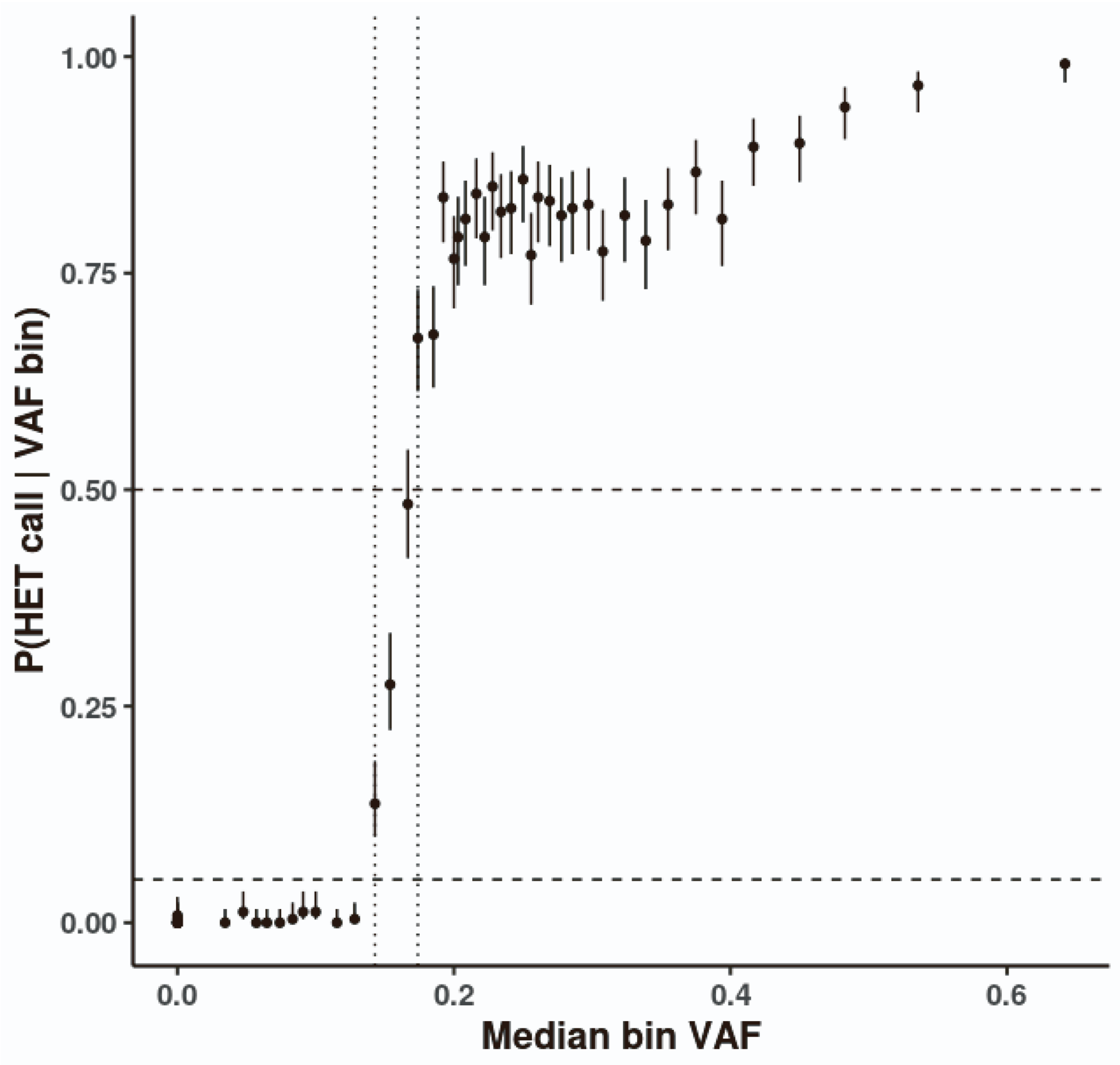

Figure S10. Platypus empirical VAF thresholds for HET genotype calling. Showing the empirical probability of HET calling by platypus across 50 VAF bins for 6,000 randomly selected Mendelian inconsistencies (12,000 genotypes across parents and offspring). Error bars are 95% Wilson score confidence intervals on the binomial proportion within each bin. Vertical dotted lines intersect VAF ≥ 0.14 and ≥ 0.17, where the probability of HET calling are 5% and 50%, respectively (dotted horizontal lines)

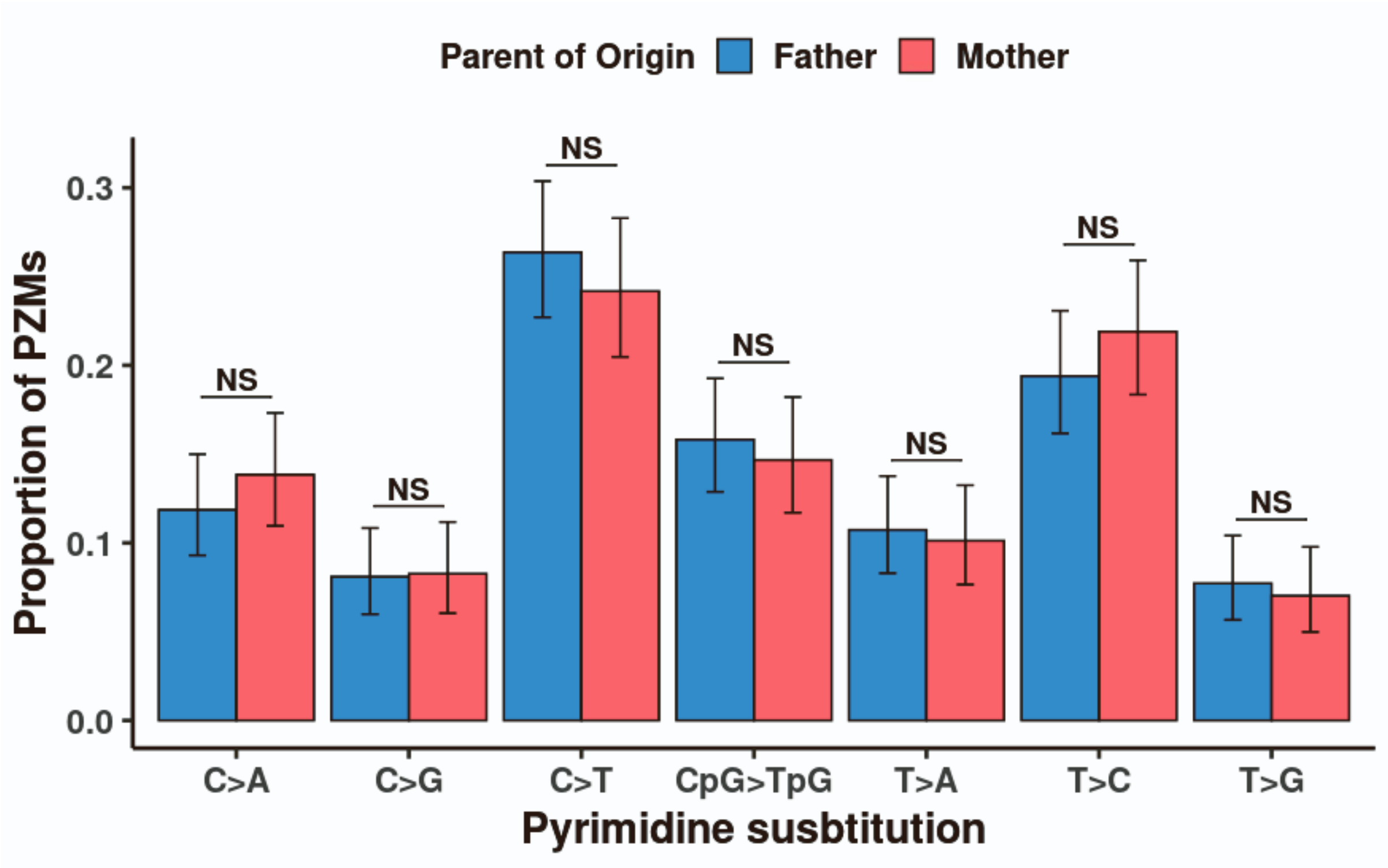

Figure S11. Spectral characteristics of PZMs stratified by sex. Bars represent 95% confidence intervals for grouped pyrimidine substitution proportion (one-sample proportion test with continuity correction).

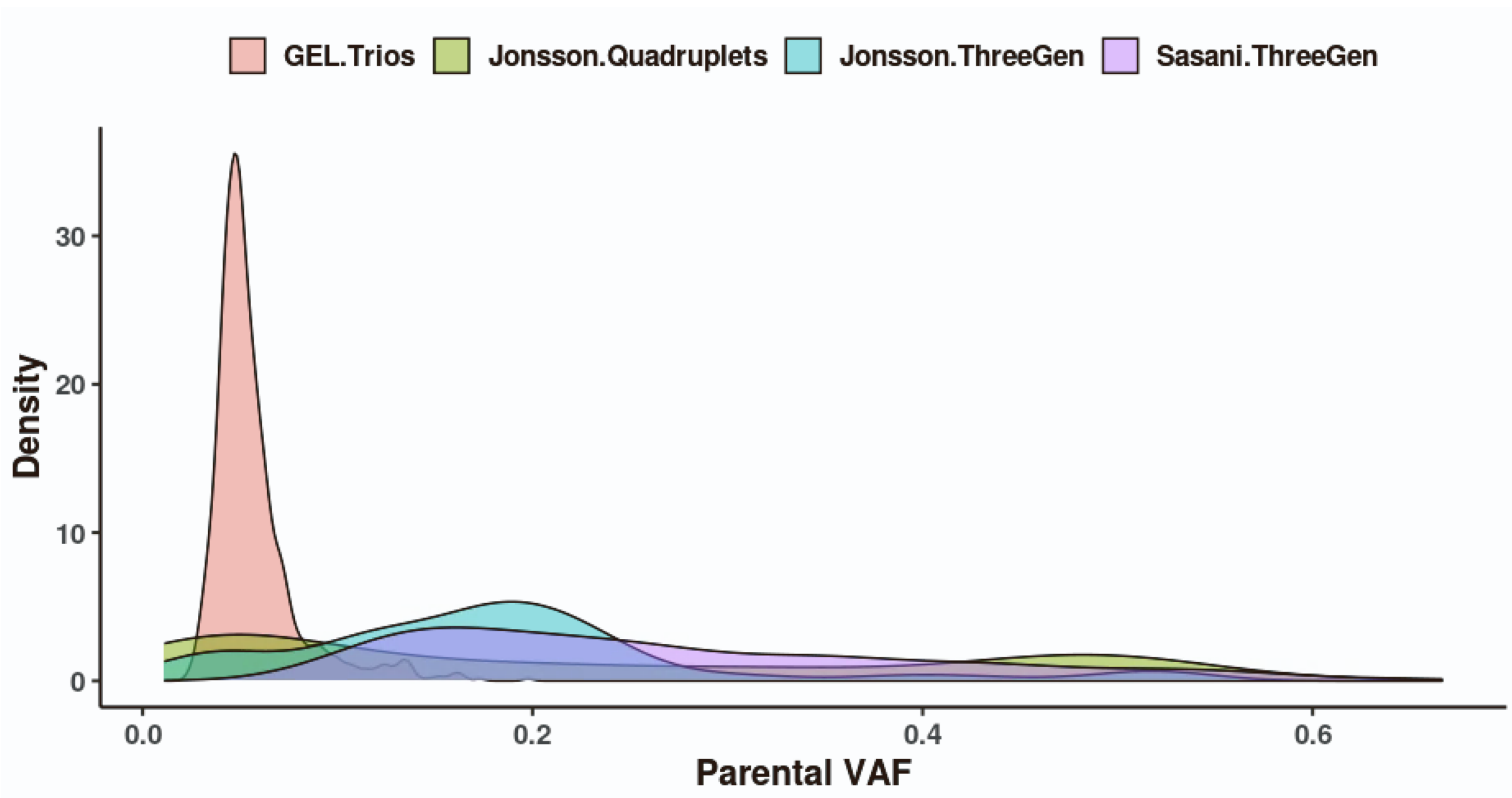

Figure S12. PZM VAF distributions stratified by study and PZM ascertainment method

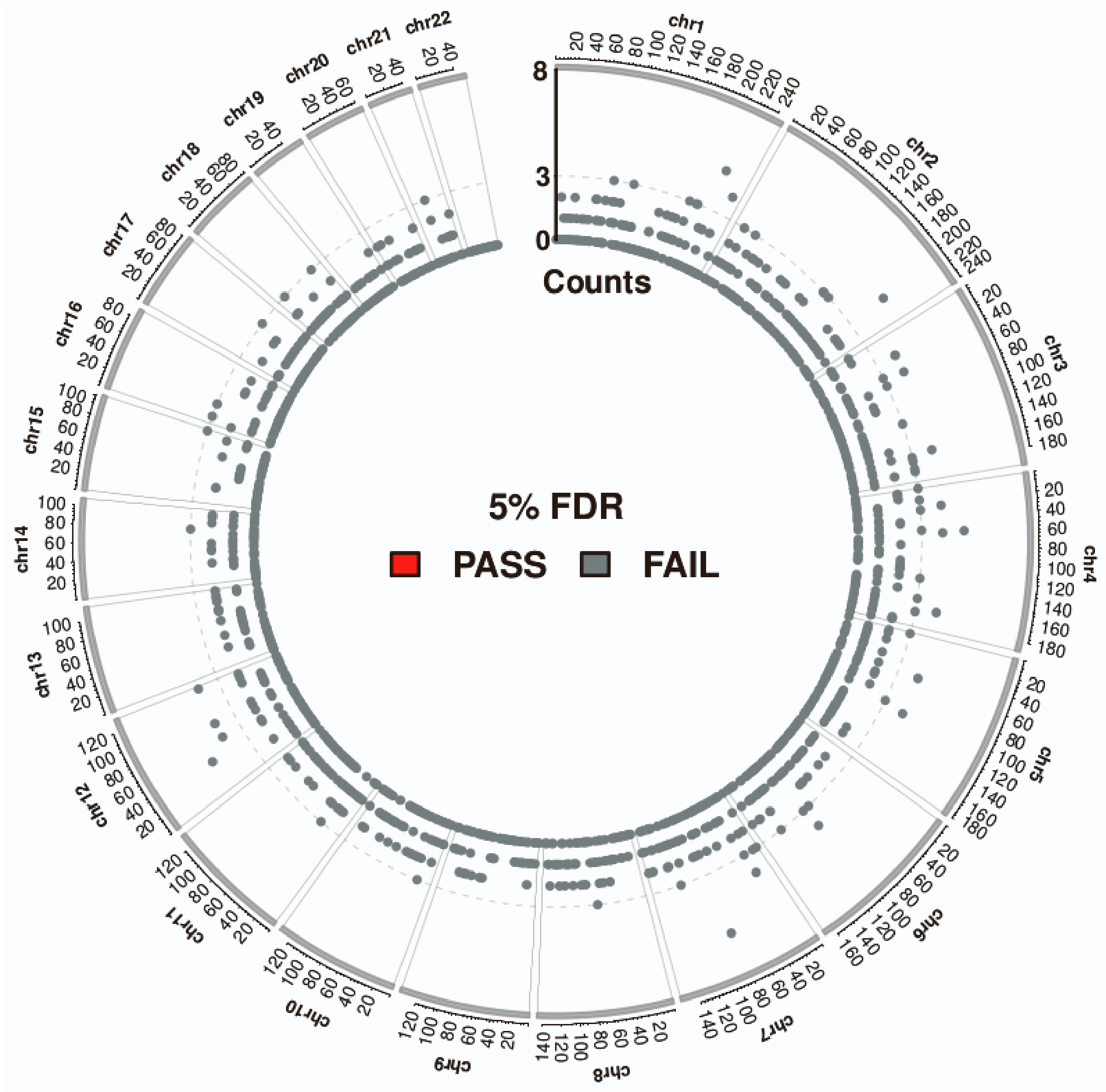

Figure 13. Genomic distribution of PZMs. Each point represents cohort-wide PZM counts for a given non-overlapping 2Mb window. No bin was found significantly enriched after multiple testing significance correction.

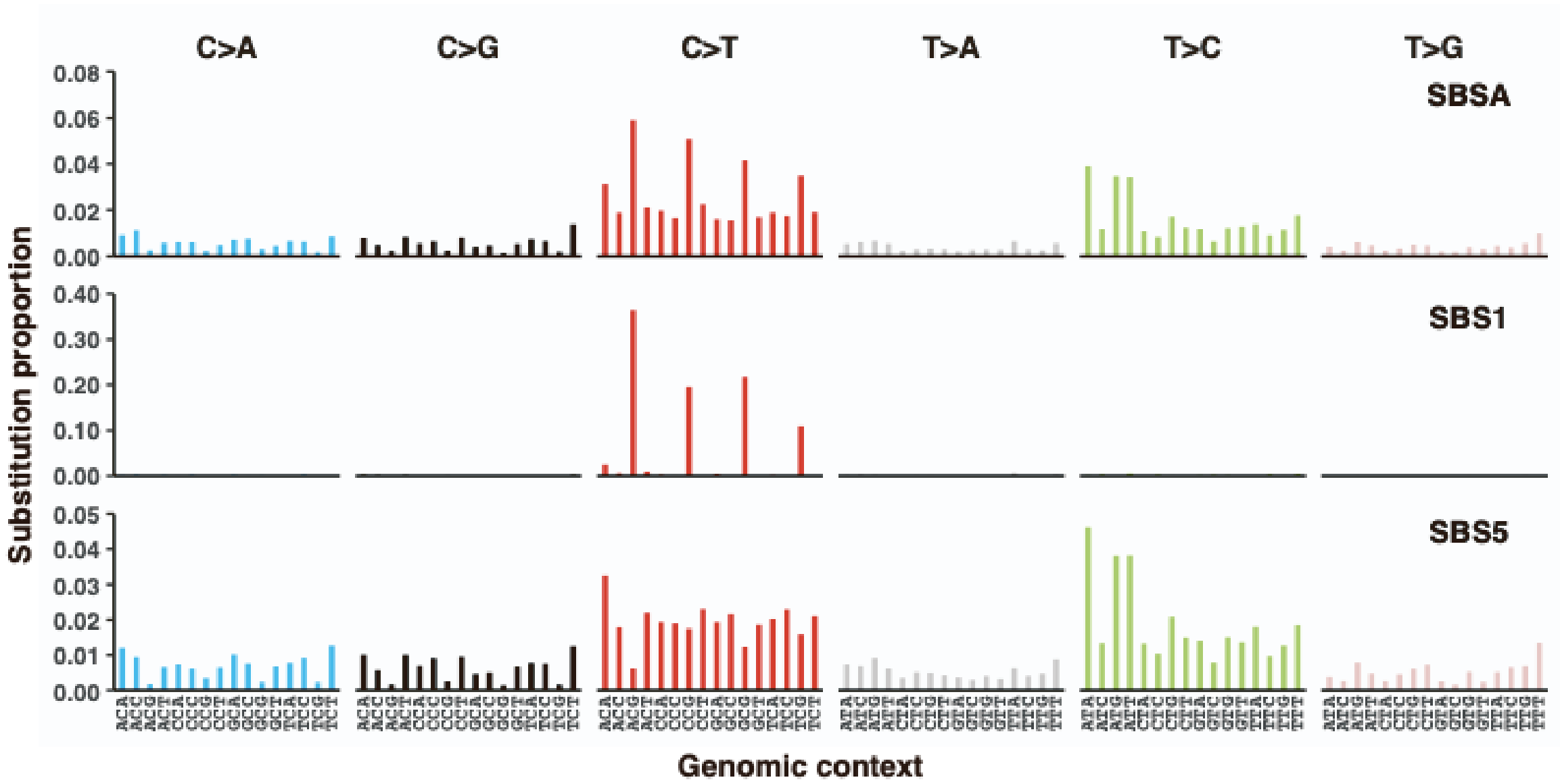

Figure S14. Mutational signature extraction and deconvolution of PZMs. Showing single nucleotide mutation proportions across the 16 possible 5’-3’ base-flanking contexts for (from top to bottom): SBSA (de novo extracted signature profile from GEL DNMs and PZMs), deconvoluted COSMIC SBS1, and and COSMIC SBS5 (cosine similarity to SBSA = 0.98).

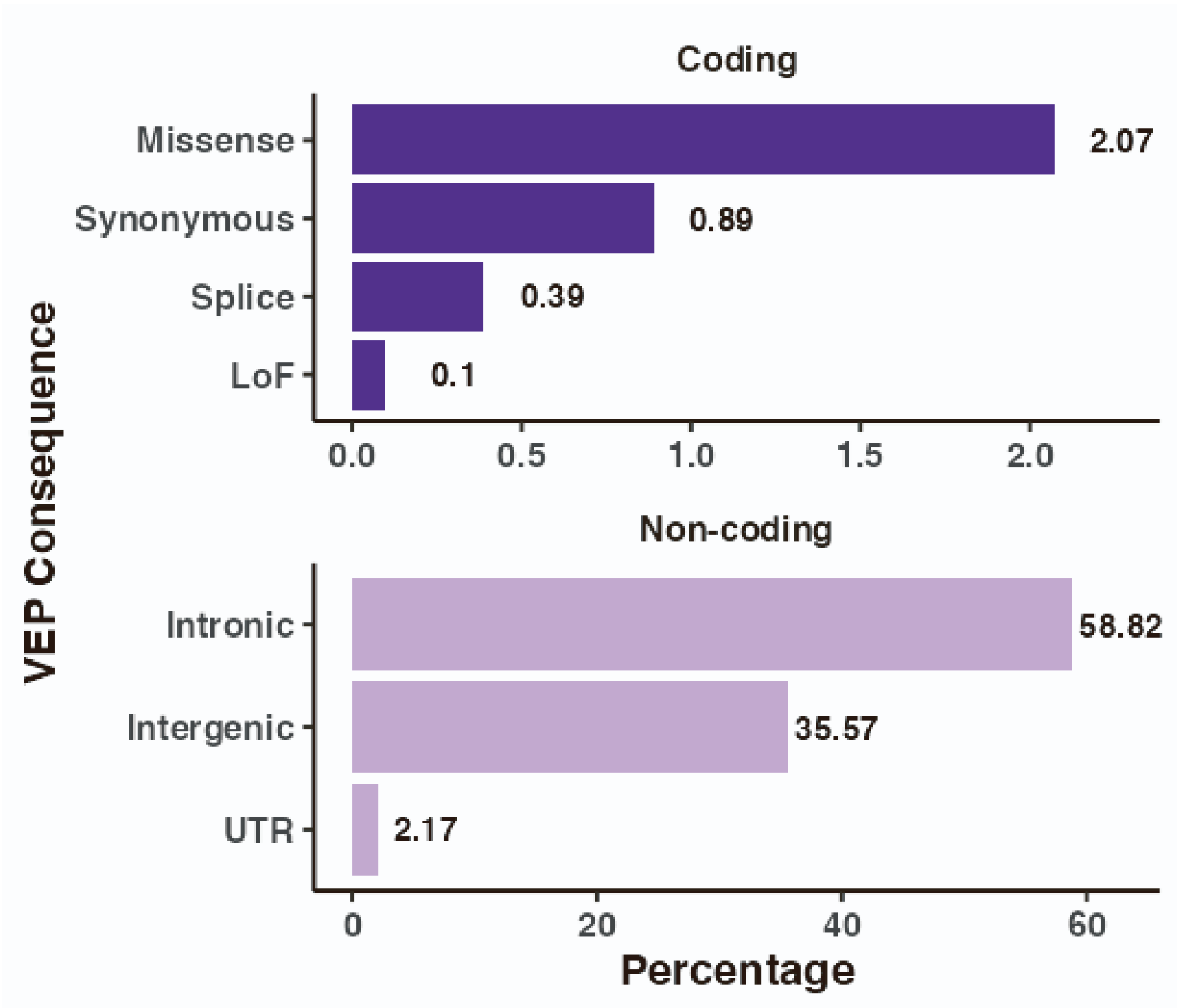

Figure S15. Consequence effect prediction for final cPZMs set (n = 1,015).

Supplemental tables

Embryonic cell division	Number of Cells	Expected cell proportion carrying the mutant allele	Expected variant allele fraction in parental somatic tissues
1	2	0.5	0.25
2	4	0.25	0.125
3	8	0.125	0.0625
4	16	0.0625	0.03125
5	32	0.03125	0.015625
6	64	0.015625	0.0078125
7	128	0.0078125	0.00390625
8	256	0.00390625	0.001953125
9	512	0.001953125	0.0009765625
10	1024	0.0009765625	0.00048828125

Table S1. Expected PZM VAFs and mutant cell proportions in adult tissues. Simplified model assuming synchronous and equal cell division, a stable diploid genome, no selection on mutant cells, and an even distribution of mutant cells across adult tissues for PZM events occurring between the 1st and 10th cell division.

Study design [Publication]	Quadruplets [Jonsson et al., 2021]	Three Generation [Jonsson et al., 2021]	Sibling shared DNMs [Sasani et al., 2019]	Three Generation [Sasani et al., 2019]	Simple trios [GEL]
Median Parental Somatic VAF [VAF range]	0.173 [0.01, 0.63]	0.176 [0.02, 0.53]	NA*	0.241 [0.10, 0.67]	0.049 [0.02,0.34]
Pyrimidine Substitution	Pyrimidine Proportion Odds Ratio (PZMs/DNMs) [95% confidence intervals]				
C>A	1.55 [1.17, 2.02]*	1.13 [0.50, 2.23]	1.55 [1.22, 1.95]*	1.22 [0.88, 1.67]	1.34 [1.11, 1.61]*
C>G	0.62 [0.42, 0.89]*	0.50 [0.16, 1.21]	0.70 [0.49, 0.96]*	0.88 [0.60, 1.27]	0.86 [0.68, 1.08]
C>T	1.24 [1.04, 1.49]*	1.26 [0.82, 1.89]	0.87 [0.71, 1.05]	1.02 [0.81, 1.27]	1.06 [0.92, 1.22]
CpG>TpG	0.78 [0.61, 0.99]*	0.30 [0.11, 0.67]*	1.55 [1.30, 1.85]*	0.85 [0.64, 1.11]	0.85 [0.71, 1.01]
T>A	1.15 [0.82, 1.58]	1.53 [0.74, 2.86]	0.73 [0.49, 1.06]	1.58 [1.11, 2.22]*	1.67 [1.35, 2.04]*
T>C	0.68 [0.54, 0.86]*	1.24 [0.81, 1.87]	0.79 [0.65, 0.96]*	0.88 [0.69, 1.11]	0.79 [0.67, 0.91]*
T>G	1.97 [1.42, 2.67]*	1.27 [0.46, 2.88]	0.67 [0.46, 0.95]*	1.06 [0.73, 1.53]	1.11 [0.86, 1.40]
Total SNV PZMs	529	102	720	442	1,015

Table S2. Differences in mutation spectra between PZMs and DNMs. Fisher's exact test odds ratios for pyrimidine substitution count differences across PZMs and DNMs identified by Sasani et al., 2019, Jonsson et al., 2021 and the Genomics England cohort. PZM counts reported by Sasani and Jonsson were stratified by the extended family trio strategy used to identify PZMs. The most likely enrichment of parental VAF for each ascertainment group is also shown. Asterisks indicate significant differences (PZM/DNMs proportions) after accounting for multiple testing correction in each PZM group (FDR≤5%).

NC_000017.11:g.8161693C>G (*VAMP2*)<br>Pathogenic (class 5, posterior probability: 0.994)	
PM2 applied (moderate)	Not present in gnomAD with adequate coverage
PS1 applied (strong)	Applied on the basis of a quality case report (PMID 37901860[42]) with the same amino acid change in a de novo state in the proband, and one Clinvar pathogenic submission from GeneDx.
PP2 applied (supporting)	Missense variants in gene are a common mechanism of disease
PP3 applied (supporting)	REVEL score: 0.628 intermediate (< 0.7); CADD score: 31 deleterious (≥ 30); SpliceAI score: 0 tolerated (≤ 0.2)
PS2 applied (moderate)	Applied at reduced strength, moderate level
NC_000014.9:g.101985930C>T (*DYNC1H1*)<br>Likely pathogenic (class 4, posterior probability: 0.949)	
PM2 applied (moderate)	Not present in gnomAD with adequate coverage
PS1 applied (moderate)	Applied on the basis of a quality case report (PMID 38953796[43]) with the same amino acid change in a de novo state in the proband; Clinvar evidence was deemed insufficient for PS1 for this variant.
PP2 applied (supporting)	Missense variants in gene are a common mechanism of disease
PP3 and BP7 not applied	REVEL score: 0.523 intermediate (< 0.7); CADD score: 26.2 intermediate (< 30); SpliceAI score: 0.01 tolerated (≤ 0.2)
PS2 applied (moderate)	Applied at reduced strength, moderate level
NC_000011.10:g.32389172C>A (*WT1*)<br>VUS (class 3, posterior probability: 0.812)	
PM2 applied (moderate)	Not present in gnomAD with adequate coverage
PP2 applied (supporting)	Missense variants in gene are a common mechanism of disease
PP3 and BP7 not applied	REVEL score: 0.311 intermediate (< 0.7); CADD score: 24.6 intermediate (< 30); SpliceAI score: 0.02 tolerated (≤ 0.2)
PS2 applied (moderate)	Applied at reduced strength, moderate level

Table S3. ACMG classification of parental PZMs with potential diagnostic relevance.

Supplemental materials and methods

Section 1. Identification of candidate parental postzygotic mutations

We accessed a comprehensive set of 14,446,487 unfiltered Mendelian inconsistencies, generated using the Platypus variant caller[1] from blood whole genome sequencing (WGS) data across 13,928 family trios, consisting of both parents and one offspring[2]. Variant calling was performed on a per trio basis, with each inconsistency representing a site where the offspring is heterozygous (HET) for the alternative (ALT) allele while both parents are homozygous for the reference (REF) allele. When multiple offspring were recruited for a given family, variant calling was performed separately for each offspring, meaning a given site could be flagged multiple times across siblings.

From this initial set, we retained only autosomal (chr1-22) single nucleotide variants (SNVs; A, T, G, C alleles) called on GRCh38-mapped genomes (n = 5,739,417). We excluded all trios previously identified to have biological or technical sources of germline hypermutation[3] yielding 5,720,018 SNVs across 11,996 trios, involving 21,644 unique parents.

From these, we excluded 1,473,685 genotypes with zero ALT reads in both parents, reasoning that these are consistent with true *de novo* germline mutations transmitted to the child rather than parental PZMs, keeping 4,246,333 genotypes . For each trio, we excluded genotypes where the ALT allele had any coverage (>0 reads) in both parents (n = 1,628,565), retaining 2,627,735 genotypes that were considered candidate postzygotic mutations (cPZMs) for downstream filtering.

Additionally, because a single family could contribute multiple trios when parents had more than one sequenced offspring, the same candidate parental PZM could be detected repeatedly via transmission to different children. Since our analyses focus on parental mosaicism rather than transmission counts, we de-duplicated these cases by retaining a single record for each unique parent–variant combination. After this step, we retained

2,574,260 cPZM genotypes spanning 753,618 unique genomic positions across 21,644 parents (mean = 118.93, Ts/Tv = 1.48).

Section 2. Bioinformatic filtering of candidate parental postzygotic mutations

We applied a rigorous, multi-step filtering pipeline to curate a high confidence set of candidate parental postzygotic mutations (**Figure S1**). Although 21,644 unique parents were originally considered, a total of 123 were removed due to missing individual or trio-level metadata. Hence, mean cPZMs per person were calculated based on a total of 21,521 parents.

1. **Removal of problematic genomic regions:** We excluded variants overlapping genomic regions prone to mapping errors, including interspersed repeats, low complexity regions (e.g. satellite repeats), centromeres, segmental duplications, alternative haplotypes, and genome assembly patches. These regions were identified from the GRCh38 RepeatMasker, Segmental Dups, Simple Repeats, GRC Patches and alternative haplotypes (fixSeqLiftOverPsl; altSeqLiftOverPsl), and Centromeres tracks from the UCSC table browser[4]. Assuming that all the bases per chromosome in the GRCh38 genome assembly (as determined in the UCSC hg38.chrom.sizes file) are callable, the removal of these regions means that our dataset is only powered to nominate PZM candidates in ~43% of the autosomal human genome. This step reduced the dataset to 237,880 cPZM genotypes over 108,468 unique positions, across 21,456 parents (mean = 11.05, Ts/Tv = 0.86).

2. **Filtering common variants:** We removed variants with a global minor allele frequency (MAF) ≥ 1% in the 1000 Genomes Project (Phase 3)[5], resulting in 203,195 cPZM genotypes at 96,030 unique positions across 21,072 parents (mean = 9.44, Ts/Tv = 0.77).

3. **Excess coverage filter:** To remove potential sequencing artefacts, we excluded variants with site depth above an individual-specific upper threshold representing the

top 1% of the expected coverage distribution, calculated using $qpois()$ in R ($lambda$ = mean whole-genome coverage; $lower.tail = FALSE$). We also removed 1,097 cPZMs from 117 parents with missing sequencing coverage metadata. After filtering 197,482 cPZM genotypes at 93,999 sites remained across 20,849 parents (mean = 9.17, Ts/Tv = 0.75).

4. **Minimum read coverage support filter:** Using $bamreadcount$[6], we calculated strand-specific ALT allele coverage for each cPZM and retained only variants with at least one ALT read on both strands. This yielded 77,548 cPZMs at 26,518 positions across 14,898 parents (mean = 3.60; Ts/Tv = 0.69). Notably, 7,120 cPZMs had been assigned ALT = 1 in Platypus due to stringent filtering on windowed read support (see $bad - reads$ parameter[7]). Because the GEL *de novo* filtering pipeline allows candidate DNMs to have a maximum parental ALT allele count of one, these variants were thus misclassified as candidate DNMs and kept in further filtering steps of that pipeline.

5. **Variant allele fraction (VAF) consistency filter:** we used $binom.test()$ in R to assess whether parental VAFs were significantly <0.5 (one-sided test), and offspring VAFs consistent with 0.5 (two-sided test). We excluded variants failing either test ($p > 0.05$ for parental VAFs, and $p \leq 0.05$ for offspring VAFs), and 26 cPZMs from 6 parents with missing offspring read data. This retained 23,651 cPZMs at 12,325 unique positions across 8,848 parents (mean = 1.09, Ts/Tv = 0.77).

6. **Read quality based on edit distance filter:** We calculated the median edit distance (NM tag) of ALT supporting reads using $samtools\ view$ and $samjdk.jar$[8]. Reads with mapping quality <20 or matching the bitflag (--excl-flags) 3844 (i.e., unmapped reads, not primary alignments, reads failing vendor quality checks, optical and PCR duplicates, and supplementary alignments) were excluded (**Figure S2**). We kept variants with median NM ≤4, yielding 4,733 cPZMs at 2,796 sites across 3,955

individuals (mean = 0.21, Ts/Tv = 1.43). Details on the derivation of the NM threshold are described in **Supplemental Methods - Section 3**.

7. **Variant-specific error filter:** To identify and exclude error-prone genomic positions, we estimated the background allelic error rate by pooling reads from 2,500 randomly selected unrelated parents at genomic sites harboring candidate PZMs (**Supplemental Methods - Section 4; Figure S3**). This background alternative allele (ALT) rate reflects the expected probability of observing a specific non-reference allele at a given genomic position under sequencing noise, shared technical artefacts, or population common variation. Importantly, this metric is distinct from a sample-level variant allele fraction and is expected to be close to zero for a real mutation event. For each position, we calculated the proportion of pooled reads supporting each nucleotide, and excluded variants showing a background ALT rate >0.00112, corresponding to the Illumina HiSeq 2500 error rate[9]). Such positions are likely to represent systematic sequencing errors or common variation rather than true low-level mosaicism This step retained 1,501 cPZMs at 1,492 sites across 1,401 parents (mean = 0.069, Ts/Tv = 1.68).

8. **Population frequency-based filtering:** We annotated the remaining cPZMs with their internal GEL cohort and gnomAD v3 population frequencies[10]. Only 9/1,501 variants were shared across multiple unrelated GEL parents. For external AFs, we found that 569/1,501 cPZMs (50.4%) were present in gnomAD. Only 98 of these variants (6.5%) were present at AFs >0.1%. To set an expectation of population allele frequency for *de novo* variation, we compared the gnomAD AFs from cPZMs with those from 778,347 filtered DNMs from the GEL cohort, previously reported by Kaplanis et al., 2022[24]. We observed that ~69% of DNMs were absent from gnomAD and 26% were present at AFs ≤0.1% (**Figure S4**). However, we found significant differences between the proportion of variants absent from gnomAD and those present in gnomAD at AFs ≤0.1% between DNMs and cPZMs (single sample

proportion test, p-value = 2.18×10^{-09}, and 2.18×10^{-05} , respectively), indicating possible contamination of cPZMs with inherited HET variation.

To test for contamination by inherited HETs or sequencing errors, we compared VAFs for cPZMs across allele frequency bins. Variants absent from gnomAD or with AFs ≤0.1% had significantly lower VAFs than common variants (Wilcoxon rank-sum test, p = 2.2×10^{-16} ; **Figure S5A**). cPZMs shared by multiple parents in the dataset also had higher VAFs than cPZMs seen in just one parent (which we refer to as 'singletons') (Wilcoxon rank-sum test, p = 3.23×10^{-07} ; **Figure S5B**). Despite the ultra-rare group showing the lowest parental VAFs, the mean VAF of singleton variants absent from gnomAD was significantly lower than that of variants present at any AF in gnomAD (one way ANOVA and Tukey HSD post-hoc testing adjusted p-value ≤0.05; **Figure S5C**). In contrast, among cPZMs shared across unrelated individuals in the GEL cohort, there was no difference in mean VAF between those absent from gnomAD versus present in any of the tested allele frequency bins in gnomAD (one way ANOVA and Tukey HSD post-hoc testing adjusted p-value > 0.05; **Figure S5C**).

Based on the described observations, and to prevent the inclusion of putative HET variants, we first removed variants having a gnomAD AF >0.1% or shared among unrelated parents, retaining 1,389 cPZMs across 1,302 parents (mean = 0.064, Ts/Tv = 1.60).

9. **Empirical HET VAF threshold-based filter:** To remove putative heterozygous variants, we estimated an individual-specific lower VAF bound for true germline HET sites using parental sequencing data. These sites were used as internal controls to characterise the distribution of VAF expected for *bona fide* heterozygous variants under each individual's sequencing depth and technical noise.

To do this, we started by obtaining germline genotypes for parents with at least one singleton cPZM (n = 1,302) from aggregated germline variant calls (AggV2) across 78,195 GEL participants in plink .pgen format, processed as described by Kousathanas et al. (2022)[40]. Genotypes were available for 1,293 individuals, as not all GEL participants were included in the aggregated dataset[41]. Using plink2, we generated an A-transposed genotype matrix (.traw) for SNPs with allele frequency between 0.2 and 0.8, identified sites called heterozygous (0/1) in at least one individual, and counted their occurrences among the 1,293 individuals.

Because no single SNP is heterozygous in all individuals, we prioritised variants that were most commonly observed in the heterozygous state across the subcohort, in order to maximise overlap while retaining sufficient SNPs per individual. For each individual, we selected two SNPs per autosome (44 SNPs total), prioritising those most common in the subcohort. This resulted in 74 unique SNPs overall, of which at least 44 were shared by ≥90% of individuals (**Figure S6A**).

We calculated VAF for each SNP using $bamreadcount$[6] (minimum base and mapping quality 20) and defined the lower bound for HET SNPs as 2.5 times the standard deviation of VAF across all SNPs in that individual (**Figure S6B**). For nine individuals lacking SNP data, we used the mean threshold from the others (mean = 0.27).

We then applied this threshold to the 1,389 remaining cPZMs, testing whether their VAFs fell significantly below the individual-specific lower bound using a one-sided $binom.test()$ (**Figure S7A**). Variants with FDR-adjusted p-value >0.05 were excluded, leaving 1,015 high-confidence cPZMs (**Figure S7B**) across 953 parents (mean VAF = 0.046, Ts/Tv = 1.57). No significant difference in parental VAFs was observed between candidates absent from versus present in gnomAD (Wilcoxon rank-sum test, p = 0.98).

To ensure that the final PZM candidate set was not likely dominated by variants called from the same DNA fragment we applied the sanity check described in **Section 5 (Figure S8)**. A summary of the number of cPZMs per individual found across the whole cohort throughout the main filtering steps in this pipeline is shown in **Figure S9**. Our final PZM catalogue had a Ts/Tv ratio of 1.57, which is lower than that expected from WGS variant calling (2-3[11]). This ratio is similar to that estimated for early PZMs reported by Sassani et al., 2019 (Ts/Tv = 1.6)[12], which is consistent with reports showing that somatic mutation catalogues are expected to have a lower Ts/Tv ratios due to different cell selection processes operating in the relevant tissues[13]. Importantly, active and passive selection processes are known to occur prior to the differentiation of the epiblast[14], where PGCs originate[15].

Section 3. Estimating an empirical maximum NM value for single nucleotide variants in GEL data

The NM metric, which is produced during the alignment step of sequencing and can be extracted from the CIGAR string, represents the number of mismatches per read[16]. To calibrate a hard threshold for median NMs in cPZMs, we extracted these values from a set of high quality common polymorphic sites. Specifically, we extracted median NMs for a set of common SNPs overlapping with high-quality mapping regions of the human genome (GRCh38 reference) from parents carrying cPZMs. To do this, from the gnomAD v3.1.1 variant set[10] and for each autosomal chromosome (chr1-22), we extracted a random selection of 1000 bi-allelic SNPs (i.e. min and max alleles = 2), which we then intersected with high-confidence mapping coordinates produced by the Illumina's Platinum Genomes Project[17,18]. These genomic regions are known to produce consistent variant calls, as these have been validated through consistent inheritance patterns across a three-generation pedigree[17,18]. To retain only common SNPs likely to be shared by most individuals regardless of ancestry, we set a filter on gnomAD global allele frequency ≥ 0.8.

As the purpose of calculating the median NM per variant is to approximate an expected number of mismatches in reads carrying the ALT allele in short read sequencing data, high quality SNPs obtained from previous steps were first compared against genotypes of the *de novo* cohort trio parents (n parents = 15,125). We checked that the alternate allele in the selected SNPs was found in heterozygous or homozygous form in at least 80% of the cPZM carriers. This was done using readily available aggregate VCF files containing filtered germline variant calls from 78,195 participants of the 100,000 genomes project[19], which includes all of the family trio parents studied in this section. Most of the selected SNPs were at least heterozygous in 80% of the family trio parents (n ~ 900 SNPs per chromosome). To reduce computing times, we only kept the 200 most common SNPs (i.e. most frequent in the cPZM carrier parents) per chromosome, and a random selection of 800 individuals for further testing. Between 4,397 and 4,600 (median = 4,600) genome-wide SNPs were tested per individual. In the same way to cPZM genotypes, we extracted reads mapping to the ALT allele in each of the selected SNPs and calculated the median NM per site and individual. We found that >98% of high-quality SNPs had a median NM ≤4 (**Figure S2**), so we used this as a hard threshold to filter cPZMs genotypes.

Section 4. Generating variant-specific error rates for genomic sites harbouring cPZMs

The variant-specific error rate is an empirical measure of the expected probability of observing a read with a given allele at a specific genomic position under random noise. In this context, it reflects either sequencing errors or common variation in the GEL cohort, with low 'error rate' variants being a product of the first instance, and variants with high 'error rate' being a product of the second.

To obtain the variant-specific error for a given genomic position, we pooled mapped reads across 2,500 randomly selected unrelated trio parents, and calculated the proportion of reads mapping to each single nucleotide allele (A, T, C, G, -). A list of unrelated individuals was readily available in the Genomics England data resources[20], and was produced by

pairwise pruning of up to third-degree related individuals (e.g., cousins and great-grandparents) based on genetic relatedness scores obtained using the KING-robust algorithm. Random individuals were selected using the R $dplyr::sample\_n()$ function setting $replace = FALSE$. Reads were pooled using the R $deepSNV::loadAllData()$ function[21]. Base and mapping quality were set to a minimum of 20, and reads with the bitflag 3844 (i.e., unmapped reads, not primary alignments, reads failing vendor quality checks, optical and PCR duplicates, and supplementary alignments) were excluded. The variant-specific error rate distributions obtained using this method for the 4,733 cPZMs remaining after step 6 of the filtering are shown in **Figure S3**.

Section 5. Ensuring independent read support for final cPZM

Of the 1,015 final PZM candidates, 604 are supported by exactly two ALT-supporting reads, one on each strand, as per our filtering in **Section 2.4**. In these cases, if both reads derive from the same read pair (e.g., due to short insert sizes causing forward and reverse reads to overlap at the variant position), they would represent a single DNA fragment rather than two independent observations. To assess this, we extracted read names for all ALT-supporting reads across all 1,015 final PZM candidates, applying the same read-filtering criteria described in **Section 2.4**. For each variant, we compared the number of unique read names to the total ALT read count: if both values are equal, each ALT-supporting read derives from an independent fragment; if the unique count is lower, at least two reads share a fragment. Only 2/1,015 PZMs showed a discrepancy between unique and total ALT read counts, with only one of these having an ALT read count of exactly two (**Figure S8**). We therefore conclude that the vast majority of our final PZM candidates are supported by at least two independent DNA fragments.

Section 6. Estimation of VAF-ascertainment bias for parental PZMs selected from Mendelian inconsistencies nominated by Platypus

Our PZM ascertainment model is conditional on diploid genotype calls from the Platypus variant caller, which is not mosaic-aware and for which putative parental PZMs present at intermediate or high VAFs are more likely to be called as heterozygous and therefore excluded at the Mendelian inconsistency selection stage, resulting in an ascertainment bias toward low-VAF parental mosaics in our final PZM catalogue.

To better understand the relationship between VAF and genotype call by Platypus, we obtained the empirical probability of obtaining a heterozygous call (0/1) for a given VAF bin. For this, from a subset of Mendelian inconsistencies matching our initial PZM candidate selection (i.e., GRCh38 autosomal single nucleotide variants, not mapping to simple repeats, segmental duplications, genome gaps, and having a non-zero parental ALT coverage), we randomly selected 6,000 variants. For the selected variants, we calculated offspring and parental VAFs from the ALT coverage and the total read depth reported by Platypus. Then, we binned the resulting VAFs into 50 bins of equal size (N = 240 genotypes per bin) and obtained the proportion of variants called heterozygous in each bin (**Figure S10**). According to this analysis, variants with a VAF ≥0.14 have at least a 5% probability of being called heterozygous by Platypus, rising to 50% at VAFs ≥0.17, implying that our pipeline has an increasing likelihood of excluding genuine mosaic variants above this VAF threshold. Furthermore, this calling behaviour has direct implications for the VAF distribution observed in our final PZM catalogue. At the average sequencing depth of this cohort (~30X), variants with a VAF below 0.14 correspond to those supported by approximately 4 or fewer alternative reads. Since these are the variants most likely to be called homozygous reference by Platypus and therefore retained as Mendelian inconsistencies, our ascertainment strategy is inherently enriched for PZMs supported by a small number of alternative reads, which directly explains the monomodal VAF distribution centred around 0.05 observed in our catalogue. This ascertainment bias is therefore a predictable consequence of applying a standard germline variant caller at standard sequencing depth,

rather than an artefact of our filtering approach.an increasing likelihood of excluding genuine mosaic variants above this VAF threshold.

Section 7. Characterisation of PZMs

A. Comparing average number of early PZMs per individual across studies

For Rahbari et al. (2016), we consulted the "Table 1" in their main text, and obtained the number of autosomal (chr1:22) SNV PZMs assigned to each parent in the three families included in this study (i.e., 6 parents). For each parent, we counted mutations marked as found in parental blood (SM or SP), or in the parental blood alone (M or P). In total, this study reports 24 PZMs meeting the above criteria across six individuals, corresponding to an average of 4 PZMs per parent.

For Sassani et al. (2019), we consulted their online supplementary material (https://github.com/quinlan-lab/ceph-dnm-manuscript), specifically the "gonosomal.dnms.summary.csv" table. This included all third generation (i.e., trio offspring) candidate DNMs where second generation individuals (i.e., in trio parents), showed incomplete linkage with heterozygous SNPs from first generation individuals (i.e., trio offspring grandparents) across 69 trio parents. To obtain the mean per individual, we summed the "snv_autosomal_dnms", which totalled 442 autosomal single nucleotide PZMs and corresponded to an average of 6.40 PZMs per individual.

For Jonsson et al. (2021) we accessed the "Supplementary Data 2", available in the online version from this publication. This table includes all candidate DNMs identified using their described quadruplets approach (i.e., candidate DNMs in the proband's offspring; absent from proband's twin and spouse/partner). From this table, we kept pre-PGC PZMs following the rule described in the "*Determination of somatic presence and dating of mutations*" section from this publication's supplementary note, after which we removed variants showing

an alternative allele read support of less than 3 reads in the proband (i.e., one of the trio parents), keeping 581 PZMs. From these, and to make a fair comparison with our data, we removed INDELs and variants mapping to the chrX, keeping a total of 523 autosomal SNV PZMs. To obtain the mean per parent, we considered all of the probands included in their quadruplet approach (n = 451).

For our study, we obtained the global average across all individuals that were not intentionally excluded (n = 123), totalling 21,521 individuals out of an initial selection of 12,644 parents. Individuals were excluded due to: a) presenting a form of true or false positive hypermutation, as reported by Kaplanis et al., (2022)[3], or b) participant ID inconsistencies in GEL data that prevented indexing metadata. Altogether, we identified an average PZM mutation rate of 0.046 PZMs per parent.

B. Association between parental ages at conception and trio DNMs and PZMs

DNMs detected in a trio are known to be associated with parental ages at conception, since this type of mutation accumulates with age in single gametes and gamete progenitor cells in the case of males[22]. Since parental PZMs are expected to have originated during the parental embryonic development, a correlation between the total number of parental PZMs detected in an offspring and the parental age at conception is not expected. To corroborate this in our data, for each trio we obtained PZMs per trio, by summing the PZMs ascertained from the mother and father. We accounted for the de-duplication of PZMs performed earlier in our filtering pipeline, and we assigned sibling-shared PZMs to the corresponding offspring (n = 20). For all offspring carrying at least one PZMs (n = 955), we obtained the unphased DNMs counts. These corresponded to a set of pre-filtered and bioinformatically ascertained per trio DNMs, previously reported by Kaplanis et al. (2022). We then used a generalised linear regression model from the “Poisson” family to model mutation counts (either total DNMs or PZMs) as a function of parental ages at conception while controlling technical covariates that could have affected the mutation calling process, as follows:

$$mutation\ counts = \beta_0 + maternal\ age\ at\ conception \bullet \beta_1 +$$
$$+ paternal\ age\ at\ conception \bullet \beta_2 +$$
$$mean\ sequencing\ depth_{mother} \bullet \beta_3 + mean\ sequencing\ depth_{father} \bullet \beta_4 +$$
$$mean\ sequencing\ depth_{offspring} \bullet \beta_6 +$$
$$percent\ aligned\ reads_{mother} \bullet \beta_5 + percent\ aligned\ reads_{father} \bullet \beta_6 +$$
$$percent\ aligned\ reads_{offspring} \bullet \beta_7 + \varepsilon$$

We found no significant association between PZM counts per trio and maternal or paternal ages at conception (p-value = 0.54, p-value = 0.420, respectively), while these were significantly associated with DNM counts (maternal effect = 7.2×10^{-3}, p-value = 6.37×10^{-14}; paternal age effect 1.8×10^{-2}, p-value < 2×10^{-16}).

C. Mutation spectra annotation of PZMs

We annotated each SNV using according to the pyrimidine change caused by the ALT allele (i.e., C>A, C>T, C>G, T>A, T>C, T>G). In addition, we differentiate between C>T sites occurring in CpG sites (CpG>TpG) and elsewhere in the genome, to account for the differential number of transitions of these genomic contexts[30,31]. Using the seven pyrimidine substitution classifications outlined above, we calculated differences across sexes and DNM events. For each group comparison (i.e., paternal vs maternal PZMs; DNMs vs PZMs), we estimated if proportions of mutations assigned to each mutation category were significantly different using the Fisher's exact test to obtain per pyrimidine substitution Odds Ratios (OR), 95% confidence intervals, and p-value for ORs. P-values for each experiment were adjusted to account for multiple comparisons using the FDR method. Since our PZM filtering explicitly removes variants at gnomAD AFs > 0.1%, we performed the same operation for GEL DNMs since "common" DNMs could be enriched on CpG>TpG transitions, which could bias our detected PZM/DNM differences.

To test if PZMs and DNMs have a different mutational origin, as defined by their mutation spectra, we performed mutational signature extraction using SigProfiler[32]. For this, we annotated the pyrimidine substitutions obtained above with their 5' and 3' contexts and generated counts matrices for the resulting combinations (n = 96). We used this as input for

SigProfiler extractor to estimate *de novo* mutational signatures. We deconvoluted the resulting signatures into reference signatures reported in the COSMIC[33] database using the relevant method in the SigProfiler software.

D. PZM/DNM mutation spectra comparison with Sasani et al. (2019) and Jonsson et al. (2021)

Sasani et al. (2019) and Jonsson et al., (2021) ascertained PZMs from candidate DNMs at whole genome scale, which can be used to contrast the mutation spectra of PZMs in our catalogue, although with some caveats. Both of the mentioned studies featured extended family-trio study designs, including combinations of parents, parental monozygotic twins, offspring siblings and/or grandparents, which allowed them to nominate PZM candidates across broader developmental windows, including those occurring around the first cell division, and having a VAF close to 0.5 in parental blood, or close and even after the differentiation of the progenitor germ cells, thus having a very low (or even null) VAF in the parental blood. Further, the use of specific extended family trio study designs (e.g., offspring, parents, and grandparents - i.e., three-generation) biases the detection of PZMs occurring at specific developmental windows, which is reflected on the VAF distribution of PZMs discovered under each strategy (**Figure S12**).

In summary, Sasani et al., (2019) detected PZMs applying WGS (~30X) in a three generation cohort including offspring, parents and grandparents (third, second, and first generation, respectively). They first detected DNM candidates present in second generation individuals at incomplete linkage with heterozygous SNPs from first generation individuals, and that were constitutively inherited by third generation individuals. This effectively ascertains PZMs likely occurring before the differentiation of PGCs in second generation individuals, and that are present as mosaics in both somatic and germline tissues. Secondly, they detected DNM candidates in third generation individuals that were shared by two or more siblings in the same family and that present no evidence of ALT read support in second

generation individuals. This effectively ascertains PZMs that occurred after or around the differentiation of PGCs, and are likely to be confined to the germline tissue in second generation individuals. Using their three generation approach, they detected 442 PZMs (median parental VAF = 0.241; parental VAF range: 0.10-0.67) and 720 DNMs using their sibling shared DNM approach. Since the latter represent events mostly confined to the germline tissue, the VAF of these events in parental somatic tissues is either null or undetectable using standard variant filtering criteria.

Jonsson et al. (2021) detected PZMs applying WGS (~30X) in a cohort of families of monozygotic twins. First, they detected PZMs using quadruplets, which comprise a standard two-generation family-trio, but where one of the parents (i.e., the proband) has a monozygotic. In this setting, they first detected PZMs by identifying DNM candidates in the trio offspring for which there was evidence of ALT read support in the proband but not their twin. Secondly, for a subset of twins whose parents had sequencing data available, they identified PZMs by identifying DNM candidates in the trio offspring for which there was evidence of ALT read support in both twins but not their parents, or the proband's partner. Both of these strategies effectively ascertain PZMs occurring before the differentiation of PGCs, but after or prior twinning, and thus later or earlier during development, respectively. Using their quadruplet and three generation approaches, Jonsson et al. (2021) detected 529 PZMs (median parental VAF = 0.173; parental VAF range: 0.01, 0.63), and 102 PZMs (median parental VAF = 0.176; parental VAF range: 0.02, 0.53).

In contrast to these extended designs, in our study PZM ascertaining relies on WGS (~30X) of only two-generation family trios, genotypes designated as REF homozygous in parents (mostly supported by ~2 reads), and include several steps aiming to reduce the chances of including inherited parental constitutive variants. This resulted in ascertainment bias towards PZMs occurring before the differentiation of PGCs, but likely after the first cell divisions, displaying a VAF range of 0.02-0.34 (median = 0.049) in parental blood.

Taking all of the above into account, we compared the pyrimidine substitution spectra between DNMs and PZMs in the GEL, Sasani et al. (2019), and Jonsson et al. (2021) datasets as described in the “Mutation spectra annotation of PZMs” section from these supplementary methods. Since the PZM ascertainment strategy influences the power to identify PZMs occurring at specific developmental timings, we split the PZMs in Sasani and Jonsson according to the type of extended family trio used to identify them. Since each study generated their own DNM catalogues, we compared PZMs in each study to their own DNM candidates to prevent batch effects. The results from each comparison are shown in **Table S2.**

We found that our catalogue recapitulated the C>A enrichment and T>C depletion of PZMs relative to DNMs reported by both Sasani and Jonsson in their PZM subsets enriched for mutations arising at later developmental stages. Notably, our catalogue also recapitulates a T>A depletion in PZMs relative to DNMs, a signal reported by Sasani et al. (2019) specifically in their earlier-development PZM group. Although our ascertainment strategy is underpowered to systematically detect high-VAF PZMs due to stringent parental genotyping filtering, we nonetheless detect a subset of such events, and this minority is sufficient to recapitulate the T>A depletion observed in Sasani et al.'s early development group (n = 442, median VAF = 0.241), which is better powered than the equivalent group in Jonsson et al. (n = 102, median VAF = 0.176). Together, these results suggest that our catalogue is enriched for PZMs arising at later developmental stages, while still retaining genuine spectral signals from earlier developmental events, lending biological credibility to the observed mutation spectra differences and confirming that our catalogue captures true PZM events despite its design constraints.

E. Testing genomic clustering of PZMs and DNMs

To assess whether mutations (PZMs or DNMs) are clustered in specific genomic regions, we performed a permutation test on binned mutation counts. We first divided the genome into

consecutive two-megabase (Mb) windows and counted the number of mutations in each bin. To generate the null distribution, we randomly reassigned the total number of observed mutations across all bins, while accounting for differences in bin size due to mappability and filtering.

Specifically, we adjusted for the callable size of each bin by subtracting the number of bases overlapping regions excluded during filtering (e.g., segmental duplications, centromeres). We then computed a per-bin probability of receiving a mutation as the callable size of the bin divided by the total callable genome size. This weighted random assignment was repeated 10,000 times to generate a null distribution of mutation counts per bin. Finally, we computed one-tailed empirical p-values per bin as the proportion of permutations in which the number of mutations assigned to a bin was equal to or greater than the observed count **(Figure S13)**. We corrected our p-values to account for multiple testing using the "Benjamini-Hochberg" method.

F. Testing the association of genomic features with clustered PZMs and DNMs

Using the bin definition outlined in the previous section, we tested whether mutation-enriched bins had differential genomic features as defined by GC content and replication timing (RT). We annotated GC content per bin by extracting the GRCh38 sequences from each bin using the R libraries GenomicRanges and Biostrings[23,24]. For each bin, we counted the total number of non "N" nucleotides and determined the GC content as the proportion of "G" and "C" nucleotides out of this total. RTs were obtained from Massey et al., (2019)[25]. These were derived from the GM12878 lymphoblastoid cell line from which RT was inferred for the S-phase sorted cells, using G1-sorted cells as baseline[25]. This profile was selected as it correlates with RT across at least 50% of profiled cell types[26,27].

We modelled the likelihood of a bin being enriched for mutations as a function of GC content, RT, and callable size using a generalised linear model with a binomial link function. Coefficients represent the change in odds of bin enrichment per unit increase in each

predictor. Because no PZM bins survived FDR correction (see **Section 6E**), enriched bins for PZMs were defined using a nominal permutation $p \leq 0.05$ threshold (n = 49). For DNMs, where sufficient bins passed multiple testing correction, enriched bins were defined at FDR-adjusted permutation $p \leq 0.05$ (n = 236). Applying this analysis to DNM-enriched bins yielded a positive association with GC content (OR = 1.08, $p = 2.15 \times 10^{-4}$) and a negative association with RT (OR = 0.60, $p = 9.58 \times 10^{-7}$) consistent with prior reports[28,29] and supporting the robustness of our approach for detecting genuine genomic feature associations.

G. Variant functional annotation and identification of potentially clinically relevant PZMs

We used the Ensembl variant effect predictor (VEP, version 112) to generate functional effect predictions for PZMs[34]. We used bcftools +split-vep plugin[35] to keep the transcript for which the worst consequence was found. VAF differences across VEP categories were assessed using a linear regression model of the gamma family to account for the skewed VAF distribution of PZMs. We first modelled VAF as a function of six VEP categories, namely: LoF, missense, splice, synonymous, UTR, and non-coding variants. We also modelled VAF as a function of variants found overlapping coding exons or not (exonic, non-exonic variants). None of the tested categories was significantly associated with PZMs VAF (p-value >0.05).

We also tested enrichment of PZMs across genomic regions predicted to have specific functions. For this, we assigned genomic coordinates (GRCh38) to one of three categories: transcriptional units (i.e., introns, exons, UTR regions), putative regulatory regions (5000 bases upstream transcription start sites or downstream transcription termination sites), and intergenic regions. Coordinates for these elements were inferred from exon, intron, and UTR coordinates from the Refseq MANE select coding transcripts, obtained from the UCSC table browser[36]. Then we used the genomic association tester (GAT) algorithm to test if the

observed number PZMs overlapping each annotation was significantly different from expectation under random chance[37].

To identify PZMs with potential clinical value, we looked at those whose VEP functional impact was classed as either HIGH or MODERATE (n = 21), and extracted the HUGO symbols for the affected genes. Then, we intersected these genes with PanelApp genes (n = 6,461)[38], keeping 12 genes (one per PZM variant) for further inspection. We then kept PZMs in genes found in gene panels in the "green" category (i.e., those with definitive evidence of causality for disease[38]), and that have a dominant effect in disease (i.e., monoallelic inheritance) according to the same resource. This resulted in the identification of five genes *(VAMP2, WT1, DYNC1H1, PHIP,* and *NLRP3*). From these, we followed up genes whose variant carrier was classed as "undiagnosed" in GEL records. We discarded *NLRP3* since the proband was reported to have been diagnosed on the basis of a different gene variant. For the rest, we inspected individual phenotype records, and compared these against the phenotypes reported by PanelApp and in the literature. We discarded *PHIP* from further description since the individuals's phenotypes were not a good match with Chung-Jansen Syndrome, associated with *PHIP* mutations[39].

The remaining three variants, all missense, in *VAMP2*, *WT1*, and *DYNC1H1*, were assessed based on the pathogenicity evidence given under the ACMG standards and guidelines[40] according to the January 2018 ClinGen SVI Bayesian classification framework[41]. For all of these cases, the PS2 criteria was applied at reduced strength (moderate) since probands are heterozygous and parents are low level mosaics and clinically unaffected. The ACMG criteria applied to each variant are detailed in **Table S3**.

Supplemental references

1. Rimmer, A., Phan, H., Mathieson, I., Iqbal, Z., Twigg, S.R.F., WGS500 Consortium, Wilkie, A.O.M., McVean, G., and Lunter, G. (2014). Integrating mapping-, assembly- and haplotype-based approaches for calling variants in clinical sequencing applications. Nat. Genet. *46*, 912–918. https://doi.org/10.1038/ng.3036.

2. Caulfield, M., Davies, J., Dennys, M., Elbahy, L., Fowler, T., Hill, S., Hubbard, T., Jostins, L., Maltby, N., Mahon-Pearson, J., et al. (2020). National Genomic Research Library. Preprint at figshare.

3. Kaplanis, J., Ide, B., Sanghvi, R., Neville, M., Danecek, P., Coorens, T., Prigmore, E., Short, P., Gallone, G., McRae, J., et al. (2022). Genetic and chemotherapeutic influences on germline hypermutation. Nature *605*, 503–508. https://doi.org/10.1038/s41586-022-04712-2.

4. Karolchik, D., Hinrichs, A.S., Furey, T.S., Roskin, K.M., Sugnet, C.W., Haussler, D., and Kent, W.J. (2004). The UCSC Table Browser data retrieval tool. Nucleic Acids Res. *32*, D493–D496. https://doi.org/10.1093/nar/gkh103.

5. 1000 Genomes Project Consortium, Auton, A., Brooks, L.D., Durbin, R.M., Garrison, E.P., Kang, H.M., Korbel, J.O., Marchini, J.L., McCarthy, S., McVean, G.A., et al. (2015). A global reference for human genetic variation. Nature *526*, 68–74. https://doi.org/10.1038/nature15393.

6. Khanna, A., Larson, D.E., Srivatsan, S.N., Mosior, M., Abbott, T.E., Kiwala, S., Ley, T.J., Duncavage, E.J., Walter, M.J., Walker, J.R., et al. (2021). Bam-readcount - rapid generation of basepair-resolution sequence metrics. ArXiv *7*, 3722. https://doi.org/10.21105/joss.03722.

7. 1. Introduction — Platypus 0.1.5 documentation https://rahmanteamdevelopment.github.io/Platypus/documentation.html.

8. Lindenbaum, P. (2015). JVarkit: java-based utilities for Bioinformatics. Preprint at figshare, https://doi.org/10.6084/M9.FIGSHARE.1425030 https://doi.org/10.6084/M9.FIGSHARE.1425030.

9. Stoler, N., and Nekrutenko, A. (2021). Sequencing error profiles of Illumina sequencing instruments. NAR Genom. Bioinform. *3*, lqab019. https://doi.org/10.1093/nargab/lqab019.

10. Chen, S., Francioli, L.C., Goodrich, J.K., Collins, R.L., Kanai, M., Wang, Q., Alföldi, J., Watts, N.A., Vittal, C., Gauthier, L.D., et al. (2024). A genomic mutational constraint map using variation in 76,156 human genomes. Nature *625*, 92–100. https://doi.org/10.1038/s41586-023-06045-0.

11. Wang, J., Raskin, L., Samuels, D.C., Shyr, Y., and Guo, Y. (2015). Genome measures used for quality control are dependent on gene function and ancestry. Bioinformatics *31*, 318–323. https://doi.org/10.1093/bioinformatics/btu668.

12. Sasani, T.A., Pedersen, B.S., Gao, Z., Baird, L., Przeworski, M., Jorde, L.B., and Quinlan, A.R. (2019). Large, three-generation human families reveal post-zygotic mosaicism and variability in germline mutation accumulation. Elife *8*, e46922. https://doi.org/10.7554/eLife.46922.

13. Xia, J., Wang, Q., Jia, P., Wang, B., Pao, W., and Zhao, Z. (2012). NGS catalog: A

database of next generation sequencing studies in humans. Hum. Mutat. *33*, E2341–E2355. https://doi.org/10.1002/humu.22096.

14. van Neerven, S.M., and Vermeulen, L. (2023). Cell competition in development, homeostasis and cancer. Nat. Rev. Mol. Cell Biol. *24*, 221–236. https://doi.org/10.1038/s41580-022-00538-y.

15. Castillo-Venzor, A., Penfold, C.A., Morgan, M.D., Tang, W.W., Kobayashi, T., Wong, F.C., Bergmann, S., Slatery, E., Boroviak, T.E., Marioni, J.C., et al. (2023). Origin and segregation of the human germline. Life Sci. Alliance *6*. https://doi.org/10.26508/lsa.202201706.

16. The SAM/BAM Format Specification Working Group Sequence alignment/map format specification. https://samtools.github.io/hts-specs/SAMv1.pdf.

17. Eberle, M.A., Fritzilas, E., Krusche, P., Källberg, M., Moore, B.L., Bekritsky, M.A., Iqbal, Z., Chuang, H.-Y., Humphray, S.J., Halpern, A.L., et al. (2017). A reference data set of 5.4 million phased human variants validated by genetic inheritance from sequencing a three-generation 17-member pedigree. Genome Res. *27*, 157–164. https://doi.org/10.1101/gr.210500.116.

18. Zook, J. (2012). Genome in a Bottle | NIST.

19. Aggregated Variant Calls (AggV2) - Genomics England Research Environment User Guide https://re-docs.genomicsengland.co.uk/aggv2/.

20. AggV2 Principal Components and genetically inferred relatedness - Genomics England Research Environment User Guide https://re-docs.genomicsengland.co.uk/principal_components/.

21. Gerstung, M., Beisel, C., Rechsteiner, M., Wild, P., Schraml, P., Moch, H., and Beerenwinkel, N. (2012). Reliable detection of subclonal single-nucleotide variants in tumour cell populations. Nat. Commun. *3*, 811. https://doi.org/10.1038/ncomms1814.

22. Moore, L., Cagan, A., Coorens, T.H.H., Neville, M.D.C., Sanghvi, R., Sanders, M.A., Oliver, T.R.W., Leongamornlert, D., Ellis, P., Noorani, A., et al. (2021). The mutational landscape of human somatic and germline cells. Nature *597*, 381–386. https://doi.org/10.1038/s41586-021-03822-7.

23. Lawrence, M., Huber, W., Pagès, H., Aboyoun, P., Carlson, M., Gentleman, R., Morgan, M.T., and Carey, V.J. (2013). Software for computing and annotating genomic ranges. PLoS Comput. Biol. *9*, e1003118. https://doi.org/10.1371/journal.pcbi.1003118.

24. Biostrings Bioconductor. https://bioconductor.org/packages/release/bioc/html/Biostrings.html.

25. Massey, D.J., Kim, D., Brooks, K.E., Smolka, M.B., and Koren, A. (2019). Next-generation sequencing enables spatiotemporal resolution of human centromere replication timing. Genes (Basel) *10*, 269. https://doi.org/10.3390/genes10040269.

26. Hansen, R.S., Thomas, S., Sandstrom, R., Canfield, T.K., Thurman, R.E., Weaver, M., Dorschner, M.O., Gartler, S.M., and Stamatoyannopoulos, J.A. (2010). Sequencing newly replicated DNA reveals widespread plasticity in human replication timing. Proc. Natl. Acad. Sci. U. S. A. *107*, 139–144. https://doi.org/10.1073/pnas.0912402107.

27. Ryba, T., Hiratani, I., Lu, J., Itoh, M., Kulik, M., Zhang, J., Schulz, T.C., Robins, A.J.,

Dalton, S., and Gilbert, D.M. (2010). Evolutionarily conserved replication timing profiles predict long-range chromatin interactions and distinguish closely related cell types. Genome Res. *20*, 761–770. https://doi.org/10.1101/gr.099655.109.

28. Besenbacher, S., Sulem, P., Helgason, A., Helgason, H., Kristjansson, H., Jonasdottir, A., Jonasdottir, A., Magnusson, O.T., Thorsteinsdottir, U., Masson, G., et al. (2016). Multi-nucleotide de novo mutations in humans. PLoS Genet. *12*, e1006315. https://doi.org/10.1371/journal.pgen.1006315.

29. Acuna-Hidalgo, R., Veltman, J.A., and Hoischen, A. (2016). New insights into the generation and role of de novo mutations in health and disease. Genome Biol. *17*, 241. https://doi.org/10.1186/s13059-016-1110-1.

30. Rahbari, R., Wuster, A., Lindsay, S.J., Hardwick, R.J., Alexandrov, L.B., Turki, S.A., Dominiczak, A., Morris, A., Porteous, D., Smith, B., et al. (2016). Timing, rates and spectra of human germline mutation. Nat. Genet. *48*, 126–133. https://doi.org/10.1038/ng.3469.

31. Cooper, D.N., and Krawczak, M. (1989). Cytosine methylation and the fate of CpG dinucleotides in vertebrate genomes. Hum. Genet. *83*, 181–188. https://doi.org/10.1007/BF00286715.

32. Islam, S.M.A., Díaz-Gay, M., Wu, Y., Barnes, M., Vangara, R., Bergstrom, E.N., He, Y., Vella, M., Wang, J., Teague, J.W., et al. (2022). Uncovering novel mutational signatures by de novo extraction with SigProfilerExtractor. Cell Genom *2*, None. https://doi.org/10.1016/j.xgen.2022.100179.

33. Sondka, Z., Dhir, N.B., Carvalho-Silva, D., Jupe, S., Madhumita, McLaren, K., Starkey, M., Ward, S., Wilding, J., Ahmed, M., et al. (2024). COSMIC: a curated database of somatic variants and clinical data for cancer. Nucleic Acids Res. *52*, D1210–D1217. https://doi.org/10.1093/nar/gkad986.

34. McLaren, W., Gil, L., Hunt, S.E., Riat, H.S., Ritchie, G.R.S., Thormann, A., Flicek, P., and Cunningham, F. (2016). The Ensembl Variant Effect Predictor. Genome Biol. *17*, 122. https://doi.org/10.1186/s13059-016-0974-4.

35. Danecek, P., Bonfield, J.K., Liddle, J., Marshall, J., Ohan, V., Pollard, M.O., Whitwham, A., Keane, T., McCarthy, S.A., Davies, R.M., et al. (2021). Twelve years of SAMtools and BCFtools. Gigascience *10*. https://doi.org/10.1093/gigascience/giab008.

36. Perez, G., Barber, G.P., Benet-Pages, A., Casper, J., Clawson, H., Diekhans, M., Fischer, C., Gonzalez, J.N., Hinrichs, A.S., Lee, C.M., et al. (2024). The UCSC Genome Browser database: 2025 update. Nucleic Acids Res. https://doi.org/10.1093/nar/gkae974.

37. Heger, A., Webber, C., Goodson, M., Ponting, C.P., and Lunter, G. (2013). GAT: a simulation framework for testing the association of genomic intervals. Bioinformatics *29*, 2046–2048. https://doi.org/10.1093/bioinformatics/btt343.

38. Stark, Z., Foulger, R.E., Williams, E., Thompson, B.A., Patel, C., Lunke, S., Snow, C., Leong, I.U.S., Puzriakova, A., Daugherty, L.C., et al. (2021). Scaling national and international improvement in virtual gene panel curation via a collaborative approach to discordance resolution. Am. J. Hum. Genet. *108*, 1551–1557. https://doi.org/10.1016/j.ajhg.2021.06.020.

39. de Fallois, J., Sieckmann, T., Schönauer, R., Petzold, F., Münch, J., Pauly, M., Vasileiou,

G., Findeisen, C., Kampmeier, A., Kuechler, A., et al. (2024). Pathogenic PHIP variants are variably associated with CAKUT. Kidney Int. Rep. *9*, 2484–2497. https://doi.org/10.1016/j.ekir.2024.05.024.

40. Richards, S., Aziz, N., Bale, S., Bick, D., Das, S., Gastier-Foster, J., Grody, W.W., Hegde, M., Lyon, E., Spector, E., et al. (2015). Standards and guidelines for the interpretation of sequence variants: a joint consensus recommendation of the American College of Medical Genetics and Genomics and the Association for Molecular Pathology. Genet. Med. *17*, 405–424. https://doi.org/10.1038/gim.2015.30.

41. Tavtigian, S.V., Greenblatt, M.S., Harrison, S.M., Nussbaum, R.L., Prabhu, S.A., Boucher, K.M., Biesecker, L.G., and ClinGen Sequence Variant Interpretation Working Group (ClinGen SVI) (2018). Modeling the ACMG/AMP variant classification guidelines as a Bayesian classification framework. Genet. Med. *20*, 1054–1060. https://doi.org/10.1038/gim.2017.210.

42. Bogue, D., Ryan, G., Wassmer, E., Research Consortium, G.E., and Naik, S. (2023). VAMP2 gene-related neurodevelopmental disorder: A differential diagnosis for Rett/Angelman-type spectrum of disorders. Mol. Syndromol. *14*, 449–456. https://doi.org/10.1159/000530150.

43. Cuccurullo, C., Cerulli Irelli, E., Ugga, L., Riva, A., D’Amico, A., Cabet, S., Lesca, G., Bilo, L., Zara, F., Iliescu, C., et al. (2024). Clinical features and genotype-phenotype correlations in epilepsy patients with de novo DYNC1H1 variants. Epilepsia *65*, 2728–2750. https://doi.org/10.1111/epi.18054.
