# Supplementary material for: Landscape of parental postzygotic mutations across >11,000 rare disease trios
Source: Am J Hum Genet. 2026 Jul 13;113(8):1762–70. doi: 10.1016/j.ajhg.2026.06.015 (PMC13504344; doi:10.1016/j.ajhg.2026.06.015)
Supplement: Supplementary file 2 [file mmc2.pdf]

Landscape of parental postzygotic mutations across >11,000 rare disease trios

Authors

O. Isaac Garcia-Salinas, Katrina A. Andrews, Rashesh Sanghvi, ..., Aylwyn Scally, Hilary C. Martin, Raheleh Rahbari

Correspondence

hcm@sanger.ac.uk (H.C.M.),
rr11@sanger.ac.uk (R.R.)

Garcia-Salinas et al. detect early parental postzygotic mutations from standard-depth trio WGS across 12,015 rare disease trios, identifying 1,015 high-confidence events. Their catalog reveals distinct mutational features of early embryonic mutagenesis and demonstrates that parental mosaicism can be recovered at scale with implications for diagnosis and recurrence risk.

Garcia-Salinas et al., 2026, The American Journal of Human Genetics *113*, 1762–1770
August 6, 2026 © 2026 The Authors. Published by Elsevier Inc. on behalf of American Society of Human Genetics.
https://doi.org/10.1016/j.ajhg.2026.06.015

CellPress

REPORT

Landscape of parental postzygotic mutations across >11,000 rare disease trios

O. Isaac Garcia-Salinas,[1] Katrina A. Andrews,[1] Rashesh Sanghvi,[1] John A. Sayer,[2] Maria Torra I Benach,[1] My H. Pham,[1] Aylwyn Scally,[3] Hilary C. Martin,[1,4,*] and Raheleh Rahbari[1,4,*]

Summary

Early postzygotic mutations (PZMs) that arise after fertilization but prior to primordial germ cell specification may be present in both somatic and germ cells, causing mosaicism in a parent and constitutive inheritance in their offspring. In clinical family-trio whole-genome sequencing (WGS), such variants are systematically missed because their sub-heterozygous variant allele fraction (VAF) prevents heterozygous calling in the parent, while residual parental allele support disqualifies the variant as a candidate germline *de novo* mutation (DNM) in the child. Here, we developed a bioinformatic approach to ascertain parental PZMs from unfiltered DNM candidates in standard-depth (∼30×) trio WGS and applied it to 12,015 trios from the Genomics England 100,000 Genomes Project. We identified 1,015 high-confidence early autosomal parental PZMs, a large single-source catalog of this mutation class. These exhibited a monomodal VAF distribution centered around 5% in parental blood, consistent with empirically characterized ascertainment boundaries imposed by standard-depth sequencing and germline variant calling. PZMs showed no parental age or sex bias and displayed a mutational spectrum distinct from that of DNMs, with enrichment for C>A and T>A substitutions and depletion of T>C. Mutational signature analysis revealed that both mutation types are shaped by clock-like signatures SBS1 and SBS5 in similar proportions, suggesting that spectral differences reflect shifts within shared mutagenic processes. Exploratory genomic distribution analysis revealed a negative PZM association with GC content, in contrast to the positive association for DNMs. Among these, we found variants in *DYNC1H1* and *WT1* with potential clinical relevance that were missed by routine diagnostic pipelines.

Most heritable mutations arise in adult germ cells throughout aging and gametogenesis and are known as germline *de novo* mutations (DNMs).[1,2] However, mutations can also arise as early as the first zygotic cell division. These are termed postzygotic mutations (PZMs).[1,3–5] Depending on developmental timing, PZMs can be classified into two categories. Early PZMs arise before somatic and germline fates are specified (approximately 2 weeks post-fertilization and within the first 10–15 cell divisions)[6,7] and generate mosaicism in both compartments when the mutation was acquired by a cell progenitor of both lineages. Germline-confined PZMs occur later in germline progenitors and are restricted to the germline lineage. Importantly, the level of mosaicism in fully developed tissues reflects the timing of the mutation, with earlier events present at higher cell fractions and more likely to recur in the mosaic individual's future offspring[8,9] (Figures 1A and 1B). In a family trio, PZMs arising in either a parent or their child can be misidentified as classical DNMs in the offspring or discarded as sequencing artifacts. Here, we focus on parental PZMs, where the mutation arose during the parent's early development and was subsequently transmitted to the offspring.

Although parental PZMs are a recognized source of sporadic genetic disease,[4,9,10] they are systematically overlooked by standard germline variant-calling pipelines, which assume constitutive heterozygous allele fractions in an individual and therefore fail to detect variants present at mosaic levels.[11] This limitation has particular consequences in the clinical sequencing of family trios (typically an affected child and two unaffected parents), where pathogenic parental PZMs escape detection through two distinct filtering mechanisms. First, because the mosaic parent usually carries the variant below the heterozygous detection threshold, it is not called as a parental genotype, yet the residual allelic support in that parent is sufficient to disqualify the variant as a DNM in the child, which requires near-absent parental evidence. Second, even in rare cases where the mosaic parent is clinically affected, the sub-heterozygous variant allele fraction (VAF) may still fall below the calling threshold for standard heterozygous variant detection. In both scenarios, the variant escapes clinical interpretation entirely, and recurrence risk to future offspring is underestimated or not communicated at all.[8,9,12]

One approach to recovering parental PZMs from trio data is to analyze the parental VAF of variants initially flagged as candidate DNMs by standard pipelines, where non-zero alternative-allele support in a parent suggests postzygotic origin followed by transmission to the offspring.[4,13] However, at standard whole-genome sequencing (WGS) depth, low-VAF variants are difficult

[1]Wellcome Sanger Institute, Wellcome Genome Campus, Hinxton, UK; [2]Biosciences Institute, Faculty of Medical Sciences, Newcastle University, Renal Services, The Newcastle upon Tyne Hospitals NHS Foundation Trust, NIHR Newcastle Biomedical Research Centre, Newcastle upon Tyne, UK; [3]Department of Genetics, University of Cambridge, Cambridge, UK
[4]These authors contributed equally
*Correspondence: hcm@sanger.ac.uk (H.C.M.), rr11@sanger.ac.uk (R.R.)
https://doi.org/10.1016/j.ajhg.2026.06.015.
© 2026 The Authors. Published by Elsevier Inc. on behalf of American Society of Human Genetics.
This is an open access article under the CC BY license (http://creativecommons.org/licenses/by/4.0/).

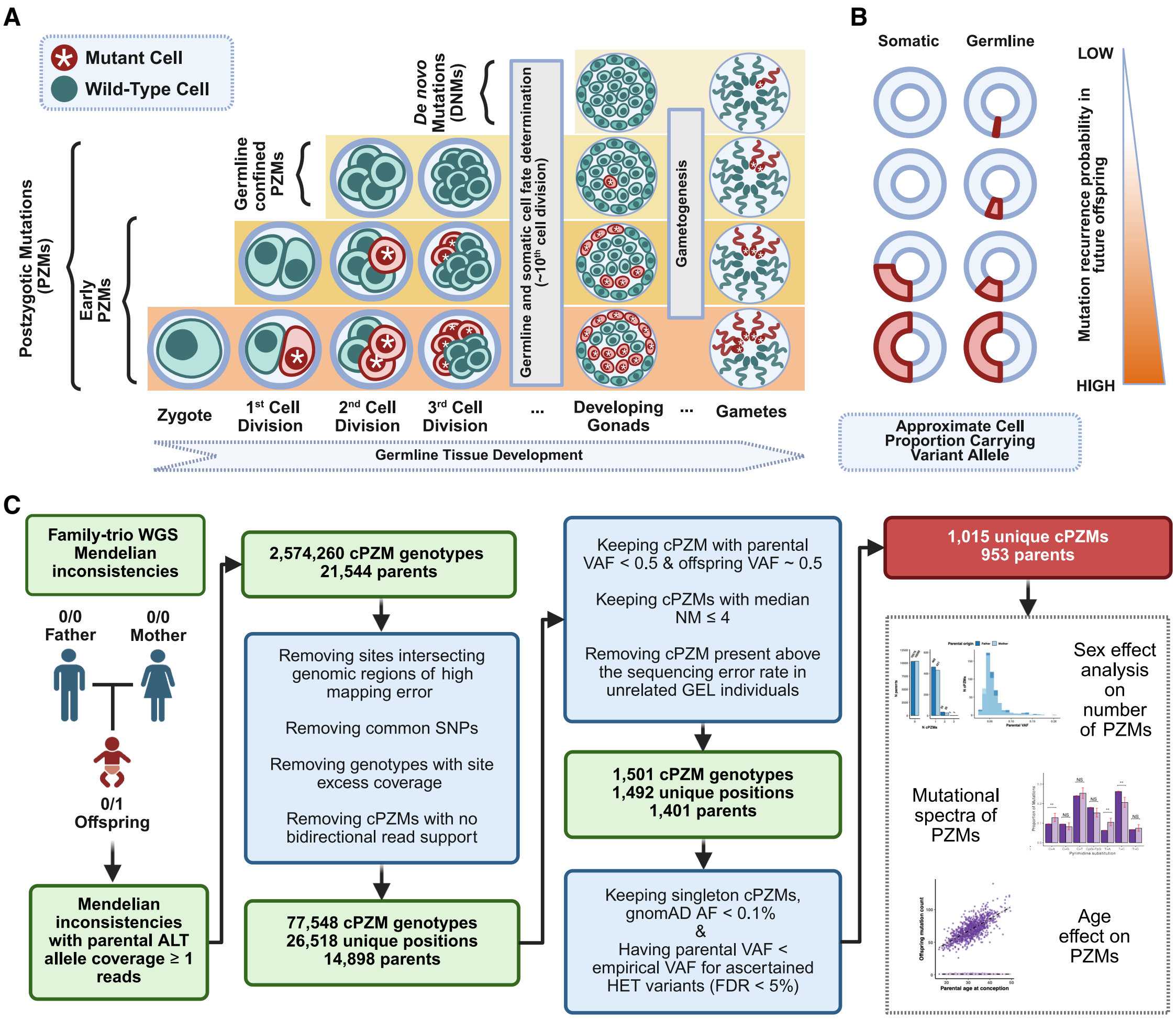

Figure 1. Origins of germline mutations and bioinformatic ascertainment of parental postzygotic mutations

(A) Classification of germline mutations according to mutation timing. Postzygotic mutations (PZMs; rows 1–3, bottom to top) can affect both somatic and germline lineages if they occur before segregation of somatic and germline fates (i.e., early PZMs) or be restricted to the germline tissue if they arise in germline progenitors (i.e., germline-confined PZMs). For both categories, the earlier a mutation occurs, the larger the fraction of future gametes that will carry it. In contrast, *de novo* mutations (DNMs,; top row) arise later in life during gametogenesis and affect only a subset of germline progenitors or single gametes. Gametes are represented with sperm cells; however, germline mutations can arise in both the male and female germline.

(B) Approximate proportions of somatic and germline cells carrying each type of mutation, with the right-hand scale indicating the likelihood of transmission to offspring.

(C) Overview of this study: analysis of trio WGS data for Mendelian inconsistencies supported by $\geq$ 1 ALT read, yielding 1,015 early candidate PZMs (cPZMs) in parents. Created in BioRender (https://BioRender.com/zrncn74).

to distinguish from sequencing and mapping artifacts, particularly in regions with poor coverage.[13,14] Statistical frameworks that integrate features such as read depth, allele balance, and quality scores can improve this discrimination,[15] but robust detection at the genome-wide scale ultimately requires either deep sequencing-based validation or extended family designs involving multiple generations or siblings, neither of which is readily scalable to large cohorts. Consequently, only three studies have attempted systematic characterization of early parental PZMs to date. Rahbari et al.[1] identified 24 PZMs across 6 parents across a multi-sibling design, while Sasani et al.[16] and Jónsson et al.[17] cataloged 442 and 523 autosomal single-nucleotide variant (SNV) PZMs from 69 and 451 parents, respectively, using extended family structures (supplemental material and methods). Collectively, these studies estimate between 1.15 and 6.4 early PZMs per individual but differ substantially in sequencing strategy, family structure, and variant filtering, limiting direct comparison.

At current scales, many genomic features of parental PZMs remain incompletely characterized. The mutational spectrum, which reflects the genomic context of mutagenesis and is informative of underlying processes,[18] illustrates this limitation. The two largest of these catalogs have shown that PZMs ascertained across different developmental windows exhibit distinct spectral features, with substitution-type composition varying between pre-PGC and germline-confined mutations.[16,17] However, these differences are often subtle,[19] and resolving them requires

catalogs with sufficient power to sample PZMs across the relevant VAF range, a constraint that existing datasets have only partially addressed.

To address these limitations, we developed a bioinformatic annotation approach to detect early parental PZMs in large family-trio cohorts (Figure 1C). Our method filters candidate DNMs within two-generation trios, retaining those in which the alternate allele is detectable in parental reads while minimizing artifacts. We applied this pipeline to 12,015 trios from the Genomics England (GEL) 100,000 Genomes Project (100kGP), an initiative aiming to identify the genetic causes of rare diseases through WGS in individuals from the United Kingdom.[20] The original GEL analysis of germline variation did not include methods for ascertaining parental PZMs, making this cohort well suited for retrospective recovery of these variants. From this dataset, we identified 1,015 high-confidence early autosomal PZMs (Figure 1C), characterized their mutational spectrum and genomic distribution, and assessed their clinical relevance in the context of rare disease diagnosis, providing further evidence that parental mosaicism represents an underrecognized inheritance mode with direct implications for diagnostic yield and recurrence risk estimation.

We accessed a previously identified set of 5,720,018 autosomal SNVs exhibiting Mendelian inconsistencies across an initial set of 11,996 trios and 21,644 unique parents (after accounting for parents sequenced as part of multiple trios), recruited through the rare disease arm of the 100kGP[21] (supplemental material and methods). Mendelian inconsistencies were defined as sites where the offspring was called as heterozygous for the alternative allele, while both parents were called as homozygous for the reference allele. These sites are also referred to as candidate DNMs since they are the starting point for DNM ascertainment in family sequencing studies. In GEL, these were identified on a per-trio basis using the variant caller Platypus, combining local realignment, haplotype assembly, and Bayesian statistical modeling.[22]

Among these, 2,627,735 had alternative allele read support in a single parent, suggesting parental postzygotic mosaicism, hereafter referred to as candidate PZMs (cPZMs). Because parents with multiple sequenced offspring could contribute the same cPZM across different trios, we retained a single record for each unique parent-variant combination, yielding an initial set of 2,574,260 cPZMs (mean = 118.93 cPZMs per parent). This set was then subjected to a custom filtering pipeline to distinguish true PZMs from sequencing artifacts and common germline polymorphisms (supplemental material and methods; Figures S1–S8), obtaining 1,015 high-confidence cPZMs, corresponding to 0.039% of the initial candidates (Figure S9).

After excluding individuals with missing metadata ($n = 123$; supplemental material and methods), we identified an average of 0.046 PZMs per parent across the remaining cohort, substantially lower than previous whole-genome estimates ranging from 1.15 to 6.4 PZMs per parent[1,16,17] (supplemental material and methods). However, this difference is expected since previous studies actively enriched for PZM detection through deep sequencing or additional family members, whereas our cohort-scale approach relies on standard sequencing depth without multigenerational or sibling data, limiting sensitivity to variants with lower somatic VAF or those approaching constitutive heterozygosity. Our per-parent estimate is therefore likely conservative.

Across all cPZMs, we observed a monomodal VAF distribution centered on 0.05 ($\pm 2.77 \times 10^{-3}$, 95% confidence interval [CI]), with no significant difference between parental sexes (Wilcoxon rank-sum test $p = 0.09$; Figure 2B). Under a simplified model assuming synchronous cell divisions, stable diploidy, no selection, and uniform contribution of mutant cells across adult tissues, this is consistent with mutations arising around the third embryonic cell division (Table S1). The observed distribution reflects both this developmental window and the technical boundaries of our ascertainment, which impose lower and upper detection limits. At the lower end, limited sensitivity at standard sequencing depth restricts detection to events occurring before approximately the 5th division (VAF$\sim$0.02; Table S1), well before PGC specification ($\sim$10th–15th division). At the upper end, detection is limited both by our deliberate exclusion of high-VAF variants to avoid germline heterozygotes and by the tendency of standard germline callers to call intermediate-to-high VAF mosaic variants as constitutive heterozygotes, excluding them at the Mendelian inconsistency stage. In our cohort, parental variants at VAFs ≥ 0.14 had an empirically estimated $\geq$5% probability of being called heterozygous by Platypus, rising to 50% at VAFs $\geq$ 0.17 (supplemental material and methods; Figure S10). Our catalog is therefore most informative for PZMs that arise in the intermediate developmental window between these boundaries.

Having established the detection boundaries of our approach, we first confirmed that our PZM catalog exhibits expected biological properties before characterizing features not previously assessed at the genome-wide scale.

The parental age effect on DNM rate is well established and has been proposed to result from the interplay between DNA damage and repair throughout aging.[23] Since PZMs arise during early development, we do not expect them to be associated with parental age. To test this, we aggregated PZM counts across both parents for each offspring and modeled the total as a function of maternal and paternal age at conception, adjusting for technical covariates (supplemental material and methods; Figure 2C). As expected, neither maternal nor paternal age showed a significant effect on PZM counts ($p = 0.5$ and 0.42, respectively). In contrast, DNM counts were significantly and positively associated with both maternal age (effect = 7.2×10^{-3}, $p = 6.37 \times 10^{-14}$) and paternal age (effect = 1.8×10^{-2}, $p < 2 \times 10^{-16}$). These results support the

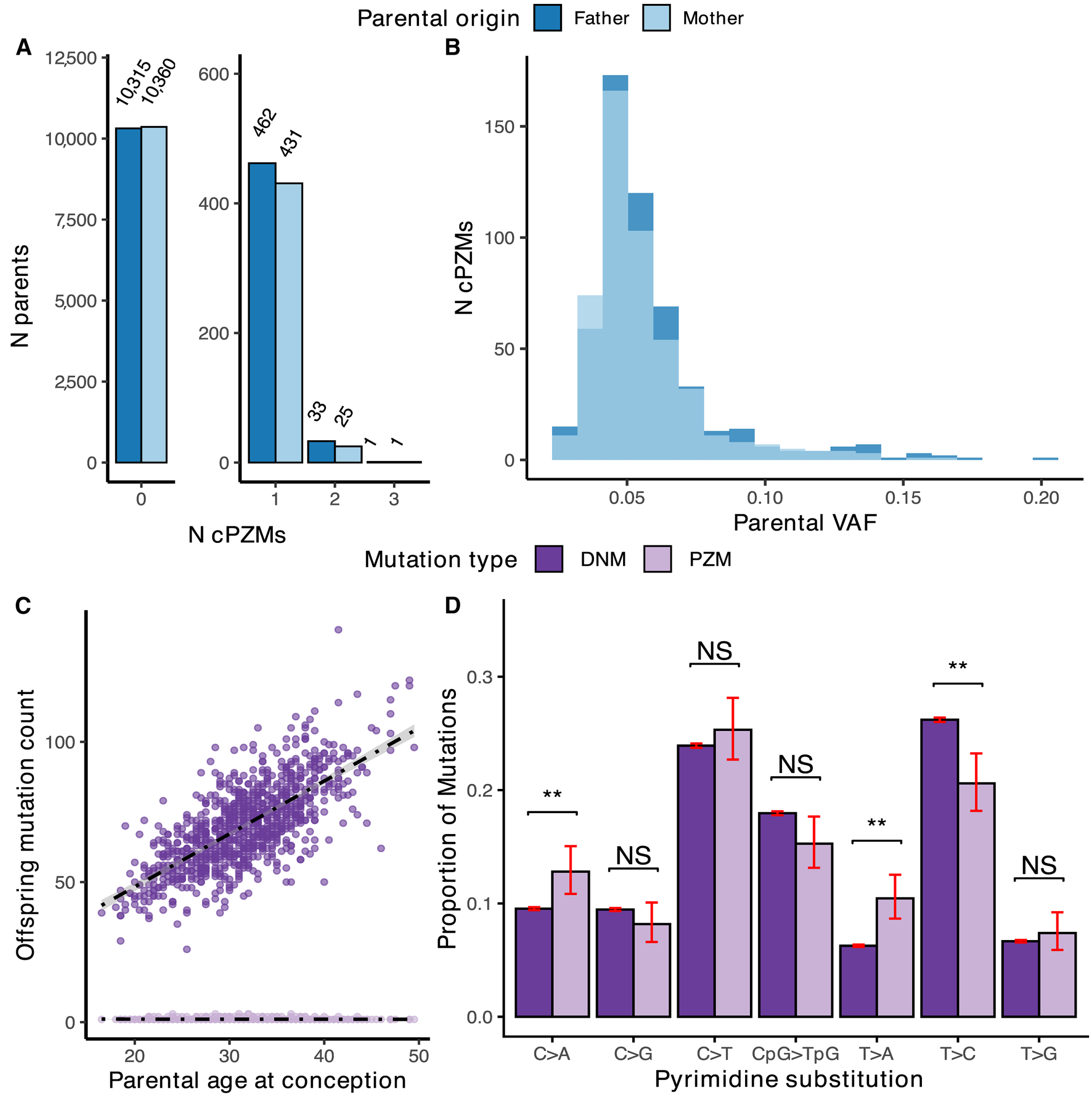

Figure 2. Characteristics of parental postzygotic mutations

(A) Distribution of PZM burden (number per individual) across the parental cohort ($n = 21{,}650$).

(B) Distribution of PZM variant allele fraction (VAF). No significant maternal vs. paternal differences in burden or VAF (Wilcoxon rank-sum $p = 0.19$ and 0.09, respectively).

(C) Effect of mean parental age at conception on offspring PZMs (light purple) and DNMs (dark purple). There was no significant age effect for PZMs (generalized linear regression $p = 0.98$).

(D) Mutation spectra comparison between PZMs and DNMs. PZMs were significantly enriched (FDR $\leq$ 5%) for C>A and T>A mutations (Fisher's exact test, adjusted $p = 5.3 \times 10^{-3}$ and 2.1×10^{-5}, respectively) and depleted for T>C (Fisher's exact test, adjusted $p = 4.5 \times 10^{-3}$) compared to DNMs. Red bars indicate 95% confidence intervals for 1-sample proportion test per pyrimidine proportion.

specificity of our PZM calls and suggest that they are not confounded by misclassified DNMs or parental-age-related somatic variants.

Consistent with the absence of a parental age effect and in line with previous observations in smaller cohorts,[1] we found no significant difference in PZM counts between parental sexes (Wilcoxon rank-sum test $p = 0.19$; Figure 2A), with 52.3% of events identified in fathers. The scale of our catalog allowed us to test this with greater statistical power than has previously been possible, lending further support to the hypothesis that the opportunity to acquire early PZMs is equivalent across sexes in humans. This contrasts with mice, where a paternal bias has been attributed to earlier PGC specification in males,[24] suggesting that these sex-specific developmental mechanisms do not operate equivalently in humans.

We next investigated the mutational spectra of PZMs to characterize the mutagenic processes underlying early developmental mutations. We classified mutations according to six substitution types (C>A, C>T, C>G, T>A, T>C, and T>G) based on the pyrimidine substitution caused by each single point mutation,[18] additionally separating CpG>TpG transitions to account for spontaneous cytosine deamination[1,25] (supplemental material and

methods). Comparing substitution-type proportions between paternal and maternal PZMs revealed no significant differences (two-sample proportion test, false discovery rate [FDR]-adjusted $p > 0.05$; Figure S11), extending the sex equivalence observed in mutation counts to the underlying mutational processes.

Comparing PZM and DNM spectra, we identified three substitution classes with significant differences in relative frequency (Fisher's exact test, FDR-adjusted $p \leq 0.05$; Figure 2D). PZMs were significantly enriched for C>A and T>A mutations relative to DNMs (PZM/DNM odds ratio [OR] = 1.34; 1.66, adjusted $p = 5.3 \times 10^{-3}$ and 2.1×10^{-5}, respectively) and significantly depleted in T>C mutations (PZM/DNM OR = 0.78; adjusted $p = 4.5 \times 10^{-3}$). To contextualize these findings, we compared our spectral profile against developmental-window-stratified PZM subsets from Sasani et al.[16] and Jonsson et al.,[17] accounting for differences in VAF distributions introduced by their extended family designs (supplemental material and methods; Figure S12; Table S2). Our C>A enrichment and T>C depletion relative to DNMs are consistent with significant patterns observed in PZM subsets enriched for events arising around or after PGC specification.[16,17] In contrast, the T>A enrichment we observe aligns with a PZM subset capturing pre-PGC events at higher parental VAFs (median = 0.241).[16] Although our catalog is centered on a narrow, low-VAF window (median = 0.049), its spectral profile does not correspond exclusively to a single developmental stage, instead reflecting a mixture of mutational signatures associated with distinct developmental windows across independent studies. This indicates that despite our ascertainment constraints, the catalog retains sufficient representation across postzygotic developmental timings to capture reproducible spectral features of postzygotic mutagenesis.

Having validated our catalog through substitution-level spectral comparisons, we next characterized the genomic distribution and mutational processes underlying early PZMs.

We investigated whether PZMs exhibit non-random genomic distributions by partitioning the genome into 2 Mb bins and testing for significant PZM excess relative to a uniform null model (supplemental material and methods). We identified 49 nominally enriched bins (one-tailed empirical $p \leq 0.05$), none of which survived multiple testing correction (FDR >5%; Figure S13). With this caveat in mind, we asked whether the nominally enriched bins were associated with GC content or replication timing (supplemental material and methods). Using binomial regression while adjusting for callable size, we found that GC content was negatively associated with PZM enrichment (OR = 0.87, $p = 0.016$), whereas replication timing showed no significant effect ($p = 0.51$). Applying the same approach to DNM-enriched bins recapitulated the established positive association with GC content and negative association with replication timing,[26,27] supporting the robustness of our method (supplemental material and methods). The negative GC content association for PZMs contrasts with the well-documented positive association for germline DNMs, which is largely driven by spontaneous deamination of methylated cytosines at CpG sites enriched in GC-rich regions.[26] One possible explanation is that the extensive DNA demethylation that occurs during post-fertilization epigenetic reprogramming[28] attenuates this source of mutagenesis in GC-rich regions during the cleavage divisions in which PZMs arise, though this interpretation should be considered speculative given the exploratory nature of our analysis.

To identify the mutational processes driving the observed spectral differences between PZMs and DNMs, we performed mutational signature extraction and deconvolution (supplemental material and methods). Both mutation types were predominantly shaped by SBS1 and SBS5[29] (Figure S14), two clock-like mutational signatures whose effects are widespread across somatic[18,30] and germline[1] tissues. SBS1 is dominated by cytosine transitions in the CpG context, and its accumulation rate increases with cellular turnover. SBS5 has a more diffuse distribution across mutation types and is thought to reflect background endogenous cellular damage to DNA.[23] Because the relative contributions of these two processes are known to vary across developmental contexts and cell types,[31] we compared the proportion of mutations attributed to SBS1 and SBS5 between PZMs and DNMs. However, we found no significant difference in the proportion of mutations attributed to SBS1 or SBS5 between PZMs and DNMs (two-sample proportion test, $p = 0.39$ and 0.47, respectively), indicating that the same mutational processes underlie both mutation types in broadly similar proportions. The C>A, T>A, and T>C differences observed at the substitution level may therefore reflect subtle context-dependent shifts in the activity of these shared processes across distinct developmental environments rather than entirely distinct mutational origins.

Finally, we assessed the functional distribution of PZMs. Consistent with the sequence composition of the genome, *in silico* consequence prediction[32] showed that 96.6% of PZMs occurred in non-exonic locations (Figure S15). We tested whether PZMs were preferentially located in specific functional contexts, including transcriptional units (introns, exons, and UTRs), putative regulatory regions (5,000 bases upstream of transcription start sites or downstream of transcription termination sites), and intergenic regions (supplemental material and methods), but found no greater-than-expected overlap with any tested category. PZM VAFs also did not differ significantly between consequence classes, suggesting that functional context does not detectably influence the retention or detection of early PZMs.

A primary goal of GEL is to identify disease-causing genetic variations in individuals suffering from rare diseases.[21] This involves evaluating pathogenic and likely pathogenic protein-coding variants that either arise *de novo* in the affected offspring or are inherited as heterozygous from an affected parent and that fall within

phenotype-relevant genes curated in PanelApp.[20,33] As parental PZMs fall outside such conventional inheritance classifications, the 1,015 variants identified here fall into two groups. The majority ($n = 917$) were not called by standard variant-calling pipelines in either parent or child and therefore represent novel variation not previously evaluated for clinical relevance. The remaining 98 correspond to parental PZMs with extremely low VAFs, typically supported by only two reads, which were initially misclassified as DNMs.

Although these 98 variants entered clinical interpretation workflows, their misclassification as DNMs leads to underestimation of recurrence risk when the variant is pathogenic. Among them, we identified a clinically relevant missense variant in *VAMP2* (MIM: 185881), a gene associated with neurodevelopmental disorders,[34] previously reported as the diagnostic variant in an individual with specific learning disability and global developmental delay (Tables 1 and S3). Although in this instance the finding did not alter clinical management because the mosaic carrier's parents were beyond reproductive age, it illustrates how parental PZMs misclassified as DNMs can carry recurrence implications that are missed under standard interpretation.

For the 917 previously untested PZMs, we intersected genes carrying protein-coding variants with a VEP "MODERATE" or "HIGH" consequence against PanelApp green genes, identifying four additional variants in genes with established clinical relevance: *WT1*, *DYNC1H1*, *PHIP*, and *NLRP3* (MIM: 607102, 600112, 612870, and 606416, respectively). For each, we reviewed the proband's diagnosis status and recorded phenotypes against gene-disease associations documented in PanelApp and the literature. The proband carrying the *NLRP3* variant had already received a diagnosis based on another pathogenic variant, and the *PHIP* variant carrier showed only a limited phenotypic match for this gene (supplemental material and methods). The remaining two variants, in *WT1* and *DYNC1H1*, were considered potentially clinically relevant for the previously undiagnosed offspring in each trio and reported back to the individuals' clinicians. In both cases, the variant was present as a somatic mosaic in an unaffected parent and transmitted to the offspring as a constitutive heterozygous variant (Tables 1 and S3).

The missense variant in *DYNC1H1* affects the canonical transcript (ENST00000360184.10, exon 8/78), is absent from gnomAD, and was classified as likely pathogenic by ClinPred and AlphaMissense and mildly pathogenic by CADD (scores = 0.97, 0.99, and 24.8, respectively). *DYNC1H1* is associated with several neuromuscular and neurodevelopmental disorders (MIM: 614228, 614563, and 158600), including seizure disorders.[35,36] Inspection of the GEL variant tiering database revealed that the same variant had been independently identified as a germline DNM in an unrelated GEL participant with seizures and had also been previously reported in the literature in an individual with a *de novo* occurrence of the variant and a seizure disorder.[37]

The missense variant in *WT1* affects the canonical transcript (ENST00000452863.10, exon 10/10), absent from gnomAD and predicted to be deleterious by CADD and ClinPred (scores = 23.4 and 0.99, respectively). Mutations in *WT1* are known to cause Wilms tumor (MIM: 194070) and gonadal and urinary tract malformations (Denys-Drash syndrome, MIM: 194080).[38] In this trio, the variant was present as a postzygotic mosaic mutation in the parent and transmitted to the offspring, who showed congenital anomalies of the kidney and urinary tract (CAKUTs), including hydroureter, vesicoureteral reflux, and proteinuria. Although *WT1* was not included in the diagnostic panel originally applied to this individual, both vesicoureteral reflux and proteinuria are recognized phenotypic features of pathogenic *WT1* variants,[39,40] and the recruiting clinician considered the variant clinically reportable for both individual clinical management and familial risk assessment. These examples illustrate the potential diagnostic value of systematic parental PZM ascertainment in recovering clinically relevant variants that would otherwise escape routine pipelines.

In conclusion, we developed a bioinformatic annotation pipeline to detect early parental mosaicism from standard-depth trio WGS. Although detection across the VAF spectrum is constrained by the boundaries imposed by standard genotype calling in the trio setting, we demonstrate that our annotation criteria enable recovery of true PZM events and provide an empirical derivation of these boundaries that can inform future efforts to identify PZMs from comparable sequencing data. Since this approach does not require specialized sequencing or extended family designs, it permitted population-scale characterization of early PZMs. While most spectral features detected are consistent with findings from smaller studies, the scale of our cohort enabled analyses, including mutational signature extraction and exploratory genome-wide distribution testing, suggesting that PZMs may be depleted in GC-rich regions, contrasting the well-documented positive association for germline DNMs. Although our method yields a lower per-family yield than approaches employing deep or long-read sequencing, it requires no additional data beyond standard clinical WGS, making it readily deployable as a lightweight annotation step in both routine trio analysis and reanalysis of existing WGS datasets. Parental PZMs, though infrequent, can cause rare conditions with direct implications for recurrence risk, and as most remain undetected by current clinical pipelines, systematic screening at scale is needed to improve diagnostic yield and outcomes for affected families.

Data and code availability

There are restrictions on the availability of the catalog of PZMs described in this manuscript due to GEL policies on the export of individual-level information. This catalog can be accessed

Table 1. Summary of clinically relevant parental PZMs in the GEL cohort

RefSeq HGVS variant IDs	Gene symbol (OMIM gene ID)	Panels (PanelApp) applied by clinical team	Reclassed DNM	Parental VAF (alternative allele/total reads)	Missense predictions	Phenotype	ClinVar status	ACMG class prediction (ACMG codes)	Clinician assessment
g.8161693C>G (GenBank: NC_000017.11); c.197G>C (GenBank: NM_014232.3); p.Arg66Pro (GenBank: NP_055047.2)	*VAMP2* (MIM: 185881)	intellectual disability	yes	3.08% (2/65)	AM = 0.99; CADD = 33; CP = 0.99	specific learning disability; global developmental delay	pathogenic	pathogenic (PM2, PS1, PP2, PP3, PS2)	variant already reported as a pathogenic DNM in this individual
g.101985930C>T (GenBank: NC_000014.9); c.1705C>T (GenBank: NM_001376.5); p.Arg569Trp (GenBank: NP_001367.2)	*DYNC1H1* (MIM: 600112)	malformations of cortical development; early onset or syndromic epilepsy	no	5.26% (2/38)	AM = 0.99; CADD = 24.8; CP = 0.97	intellectual disability; bilateral tonic-clonic seizure	pathogenic for different phenotype; no entry for relevant phenotype	likely pathogenic (PM2, PS1, PP2, PS2)	not available for this individual; same variant occurred as DNM in another GEL individual in whom it was determined to be pathogenic
g.32389172C>A (GenBank: NC_000011.10); c.1455G>T (GenBank: NM_024426.6); p.Lys485Asn (GenBank: NP_077744.4)	*WT1* (MIM: 607102)	proteinuric renal disease; unexplained kidney failure in young people	no	3.33% (2/60)	AM = N/A; CADD = 23.4; CP = 0.99	hydroureter; vesicoureteral reflux; proteinuria	not in ClinVar	VUS (PM2, PP2, PS2)	variant considered clinically reportable after review by clinician

A description of the codes used for the ACMG class assignment is provided in Table S3. MCD, malformations of cortical development; AM, AlphaMissense; CP, ClinPred; N/A, not available.

from the GEL research environment upon request to join the GEL research network at https://www.genomicsengland.co.uk/join-us/academics. In the GEL research environment, our catalog of PZMs and its associated code can be found in the following path: /re_gecip/shared_allGeCIPs/parental_pzm_cat.

Acknowledgments

We thank the Wellcome Trust for funding this research. R.R. and H.C.M. were supported by the Wellcome grant 220540/Z/20/A. This research was made possible through access to data in the National Genomic Research Library, which is managed by Genomics England Limited (a wholly owned company of the Department of Health and Social Care). The National Genomic Research Library holds data provided by patients and collected by the NHS as part of their care, as well as data collected as part of their participation in research. The National Genomic Research Library is funded by the National Institute for Health Research and NHS England. We also thank Graciella Martin Rijo for generously sharing her views and suggestions on data visualization. For the purpose of open access, the authors have applied a CC-BY public copyright license to any author-accepted manuscript version arising from this submission.

Author contributions

O.I.G.-S. conducted the analyses and drafted the manuscript; R.S., M.T.I.B., and M.H.P. advised on QC or specific analyses and contributed to the generation of figures; K.A.A. and J.A.S. provided clinical interpretation; A.S. provided scientific advice; and H.C.M. and R.R. jointly supervised the study.

Declaration of interests

The authors declare no competing interests.

Web resources

GenBank, https://www.ncbi.nlm.nih.gov/genbank/.
OMIM, https://www.omim.org.

Supplemental information

Supplemental information can be found online at https://doi.org/10.1016/j.ajhg.2026.06.015.

Received: September 16, 2025
Accepted: June 23, 2026
Published: July 13, 2026

References

1. Rahbari, R., Wuster, A., Lindsay, S.J., Hardwick, R.J., Alexandrov, L.B., Turki, S.A., Dominiczak, A., Morris, A., Porteous, D., Smith, B., et al. (2016). Timing, rates and spectra of human germline mutation. Nat. Genet. *48*, 126–133. https://doi.org/10.1038/ng.3469.
2. Goldmann, J.M., Veltman, J.A., and Gilissen, C. (2019). De Novo mutations reflect development and aging of the human germline. Trends Genet. *35*, 828–839. https://doi.org/10.1016/j.tig.2019.08.005.
3. Jónsson, H., Sulem, P., Arnadottir, G.A., Pálsson, G., Eggertsson, H.P., Kristmundsdottir, S., Zink, F., Kehr, B., Hjorleifsson, K.E., Jensson, B.Ö., et al. (2018). Multiple transmissions of de novo mutations in families. Nat. Genet. *50*, 1674–1680. https://doi.org/10.1038/s41588-018-0259-9.
4. Acuna-Hidalgo, R., Bo, T., Kwint, M.P., van de Vorst, M., Pinelli, M., Veltman, J.A., Hoischen, A., Vissers, L.E.L.M., and Gilissen, C. (2015). Post-zygotic point mutations are an underrecognized source of DE Novo genomic variation. Am. J. Hum. Genet. *97*, 67–74. https://doi.org/10.1016/j.ajhg.2015.05.008.
5. Martínez-Glez, V., Tenorio, J., Nevado, J., Gordo, G., Rodríguez-Laguna, L., Feito, M., de Lucas, R., Pérez-Jurado, L.A., Ruiz Pérez, V.L., Torrelo, A., et al. (2020). A six-attribute classification of genetic mosaicism. Genet. Med. *22*, 1743–1757. https://doi.org/10.1038/s41436-020-0877-3.
6. Scally, A. (2016). Mutation rates and the evolution of germline structure. Philos. Trans. R. Soc. Lond. B Biol. Sci. *371*, 20150137. https://doi.org/10.1098/rstb.2015.0137.
7. Wen, L., and Tang, F. (2019). Human germline cell development: From the perspective of single-cell sequencing. Mol. Cell *76*, 320–328. https://doi.org/10.1016/j.molcel.2019.08.025.
8. Bernkopf, M., Abdullah, U.B., Bush, S.J., Wood, K.A., Ghaffari, S., Giannoulatou, E., Koelling, N., Maher, G.J., Thibaut, L.M., Williams, J., et al. (2023). Personalized recurrence risk assessment following the birth of a child with a pathogenic de novo mutation. Nat. Commun. *14*, 853. https://doi.org/10.1038/s41467-023-36606-w.
9. Wright, C.F., Prigmore, E., Rajan, D., Handsaker, J., McRae, J., Kaplanis, J., Fitzgerald, T.W., FitzPatrick, D.R., Firth, H.V., and Hurles, M.E. (2019). Clinically-relevant postzygotic mosaicism in parents and children with developmental disorders in trio exome sequencing data. Nat. Commun. *10*, 2985. https://doi.org/10.1038/s41467-019-11059-2.
10. Veltman, J.A., and Brunner, H.G. (2012). De novo mutations in human genetic disease. Nat. Rev. Genet. *13*, 565–575. https://doi.org/10.1038/nrg3241.
11. Campbell, I.M., Shaw, C.A., Stankiewicz, P., and Lupski, J.R. (2015). Somatic mosaicism: implications for disease and transmission genetics. Trends Genet. *31*, 382–392. https://doi.org/10.1016/j.tig.2015.03.013.
12. Breuss, M.W., Antaki, D., George, R.D., Kleiber, M., James, K.N., Ball, L.L., Hong, O., Mitra, I., Yang, X., Wirth, S.A., et al. (2020). Autism risk in offspring can be assessed through quantification of male sperm mosaicism. Nat. Med. *26*, 143–150. https://doi.org/10.1038/s41591-019-0711-0.
13. Zemet, R., Van den Veyver, I.B., and Stankiewicz, P. (2022). Parental mosaicism for apparent de novo genetic variants: Scope, detection, and counseling challenges. Prenat. Diagn. *42*, 811–821. https://doi.org/10.1002/pd.6144.
14. Gerstung, M., Beisel, C., Rechsteiner, M., Wild, P., Schraml, P., Moch, H., and Beerenwinkel, N. (2012). Reliable detection of subclonal single-nucleotide variants in tumour cell populations. Nat. Commun. *3*, 811. https://doi.org/10.1038/ncomms1814.
15. Petrackova, A., Vasinek, M., Sedlarikova, L., Dyskova, T., Schneiderova, P., Novosad, T., Papajik, T., and Kriegova, E. (2019). Standardization of sequencing coverage depth in NGS: Recommendation for detection of clonal and subclonal mutations in cancer diagnostics. Front. Oncol. *9*, 851. https://doi.org/10.3389/fonc.2019.00851.

16. Sasani, T.A., Pedersen, B.S., Gao, Z., Baird, L., Przeworski, M., Jorde, L.B., and Quinlan, A.R. (2019). Large, three-generation human families reveal post-zygotic mosaicism and variability in germline mutation accumulation. eLife *8*, e46922. https://doi.org/10.7554/eLife.46922.
17. Jonsson, H., Magnusdottir, E., Eggertsson, H.P., Stefansson, O.A., Arnadottir, G.A., Eiriksson, O., Zink, F., Helgason, E.A., Jonsdottir, I., Gylfason, A., et al. (2021). Differences between germline genomes of monozygotic twins. Nat. Genet. *53*, 27–34. https://doi.org/10.1038/s41588-020-00755-1.
18. Alexandrov, L.B., Nik-Zainal, S., Wedge, D.C., Aparicio, S.A.J.R., Behjati, S., Biankin, A.V., Bignell, G.R., Bolli, N., Borg, A., Børresen-Dale, A.-L., et al. (2013). Signatures of mutational processes in human cancer. Nature *500*, 415–421. https://doi.org/10.1038/nature12477.
19. Kim, Y.-A., Leiserson, M.D.M., Moorjani, P., Sharan, R., Wojtowicz, D., and Przytycka, T.M. (2021). Mutational signatures: From methods to mechanisms. Annu. Rev. Biomed. Data Sci. *4*, 189–206. https://doi.org/10.1146/annurev-biodatasci-122320-120920.
20. Caulfield, M., Davies, J., Dennys, M., Elbahy, L., Fowler, T., Hill, S., Hubbard, T., Jostins, L., Maltby, N., Mahon-Pearson, J., et al. (2020). National Genomic Research Library. https://www.genomicseducation.hee.nhs.uk/supporting-the-nhs-genomic-medicine-service/national-genomic-research-library-information-for-clinicians/.
21. Smedley, D., Smith, K.R., Martin, A., Thomas, E.A., McDonagh, E.M., Cipriani, V., Ellingford, J.M., Arno, G., Tucci, A., Vandrovcova, J., et al. (2021). 100,000 Genomes Pilot on Rare-Disease Diagnosis in Health Care — Preliminary Report. N. Engl. J. Med. *385*, 1868–1880. https://doi.org/10.1056/NEJMoa2035790.
22. Rimmer, A., Phan, H., Mathieson, I., Iqbal, Z., Twigg, S.R.F., WGS500 Consortium, Wilkie, A.O.M., McVean, G., and Lunter, G. (2014). Integrating mapping-assembly- and haplotype-based approaches for calling variants in clinical sequencing applications. Nat. Genet. *46*, 912–918. https://doi.org/10.1038/ng.3036.
23. Spisak, N., de Manuel, M., Milligan, W., Sella, G., and Przeworski, M. (2024). The clock-like accumulation of germline and somatic mutations can arise from the interplay of DNA damage and repair. PLoS Biol. *22*, e3002678. https://doi.org/10.1371/journal.pbio.3002678.
24. Lindsay, S.J., Rahbari, R., Kaplanis, J., Keane, T., and Hurles, M.E. (2019). Similarities and differences in patterns of germline mutation between mice and humans. Nat. Commun. *10*, 4053. https://doi.org/10.1038/s41467-019-12023-w.
25. Cooper, D.N., and Krawczak, M. (1989). Cytosine methylation and the fate of CpG dinucleotides in vertebrate genomes. Hum. Genet. *83*, 181–188. https://doi.org/10.1007/BF00286715.
26. Besenbacher, S., Sulem, P., Helgason, A., Helgason, H., Kristjansson, H., Jonasdottir, A., Jonasdottir, A., Magnusson, O.T., Thorsteinsdottir, U., Masson, G., et al. (2016). Multi-nucleotide de novo mutations in humans. PLoS Genet. *12*, e1006315. https://doi.org/10.1371/journal.pgen.1006315.
27. Acuna-Hidalgo, R., Veltman, J.A., and Hoischen, A. (2016). New insights into the generation and role of de novo mutations in health and disease. Genome Biol. *17*, 241. https://doi.org/10.1186/s13059-016-1110-1.
28. Guo, H., Zhu, P., Yan, L., Li, R., Hu, B., Lian, Y., Yan, J., Ren, X., Lin, S., Li, J., et al. (2014). The DNA methylation landscape of human early embryos. Nature *511*, 606–610. https://doi.org/10.1038/nature13544.
29. Sondka, Z., Dhir, N.B., Carvalho-Silva, D., Jupe, S., Ahmed, M., Madhumita, McLaren, K., Starkey, M., Ward, S., Wilding, J., et al. (2024). COSMIC: a curated database of somatic variants and clinical data for cancer. Nucleic Acids Res. *52*, D1210–D1217. https://doi.org/10.1093/nar/gkad986.
30. Moore, L., Cagan, A., Coorens, T.H.H., Neville, M.D.C., Sanghvi, R., Sanders, M.A., Oliver, T.R.W., Leongamornlert, D., Ellis, P., Noorani, A., et al. (2021). The mutational landscape of human somatic and germline cells. Nature *597*, 381–386. https://doi.org/10.1038/s41586-021-03822-7.
31. Kaplanis, J., Ide, B., Sanghvi, R., Neville, M., Danecek, P., Coorens, T., Prigmore, E., Short, P., Gallone, G., McRae, J., et al. (2022). Genetic and chemotherapeutic influences on germline hypermutation. Nature *605*, 503–508. https://doi.org/10.1038/s41586-022-04712-2.
32. McLaren, W., Gil, L., Hunt, S.E., Riat, H.S., Ritchie, G.R.S., Thormann, A., Flicek, P., and Cunningham, F. (2016). The Ensembl Variant Effect Predictor. Genome Biol. *17*, 122. https://doi.org/10.1186/s13059-016-0974-4.
33. Martin, A.R., Williams, E., Foulger, R.E., Leigh, S., Daugherty, L.C., Niblock, O., Leong, I.U.S., Smith, K.R., Gerasimenko, O., Haraldsdottir, E., et al. (2019). PanelApp crowdsources expert knowledge to establish consensus diagnostic gene panels. Nat. Genet. *51*, 1560–1565. https://doi.org/10.1038/s41588-019-0528-2.
34. Bogue, D., Ryan, G., Wassmer, E., Research Consortium, G.E., and Naik, S. (2023). VAMP2 gene-related neurodevelopmental disorder: A differential diagnosis for Rett/Angelman-type spectrum of disorders. Mol. Syndromol. *14*, 449–456. https://doi.org/10.1159/000530150.
35. Becker, L.-L., Dafsari, H.S., Schallner, J., Abdin, D., Seifert, M., Petit, F., Smol, T., Bok, L., Rodan, L., Krapels, I., et al. (2020). The clinical-phenotype continuum in DYNC1H1-related disorders-genomic profiling and proposal for a novel classification. J. Hum. Genet. *65*, 1003–1017. https://doi.org/10.1038/s10038-020-0803-1.
36. Möller, B., Becker, L.-L., Saffari, A., Afenjar, A., Coci, E.G., Williamson, R., Ward-Melver, C., Gibaud, M., Sedláčková, L., Laššuthová, P., et al. (2025). The expanding clinical and genetic spectrum of DYNC1H1-related disorders. Brain *148*, 597–612. https://doi.org/10.1093/brain/awae183.
37. Cuccurullo, C., Cerulli Irelli, E., Ugga, L., Riva, A., D'Amico, A., Cabet, S., Lesca, G., Bilo, L., Zara, F., Iliescu, C., et al. (2024). Clinical features and genotype-phenotype correlations in epilepsy patients with de novo DYNC1H1 variants. Epilepsia *65*, 2728–2750. https://doi.org/10.1111/epi.18054.
38. Lipska-Ziętkiewicz, B.S. (1993). WT1 disorder. In GeneReviews(®) (University of Washington).
39. Lipska, B.S., Ranchin, B., Iatropoulos, P., Gellermann, J., Melk, A., Ozaltin, F., Caridi, G., Seeman, T., Tory, K., Jankauskiene, A., et al. (2014). Genotype-phenotype associations in WT1 glomerulopathy. Kidney Int. *85*, 1169–1178. https://doi.org/10.1038/ki.2013.519.
40. Lehnhardt, A., Karnatz, C., Ahlenstiel-Grunow, T., Benz, K., Benz, M.R., Budde, K., Büscher, A.K., Fehr, T., Feldkötter, M., Graf, N., et al. (2015). Clinical and molecular characterization of patients with heterozygous mutations in wilms tumor suppressor gene 1. Clin. J. Am. Soc. Nephrol. *10*, 825–831. https://doi.org/10.2215/CJN.10141014.

The American Journal of Human Genetics, Volume *113*

Supplemental information

Landscape of parental postzygotic mutations across >11,000 rare disease trios

O. Isaac Garcia-Salinas, Katrina A. Andrews, Rashesh Sanghvi, John A. Sayer, Maria Torra I Benach, My H. Pham, Aylwyn Scally, Hilary C. Martin, and Raheleh Rahbari

Supplemental figures

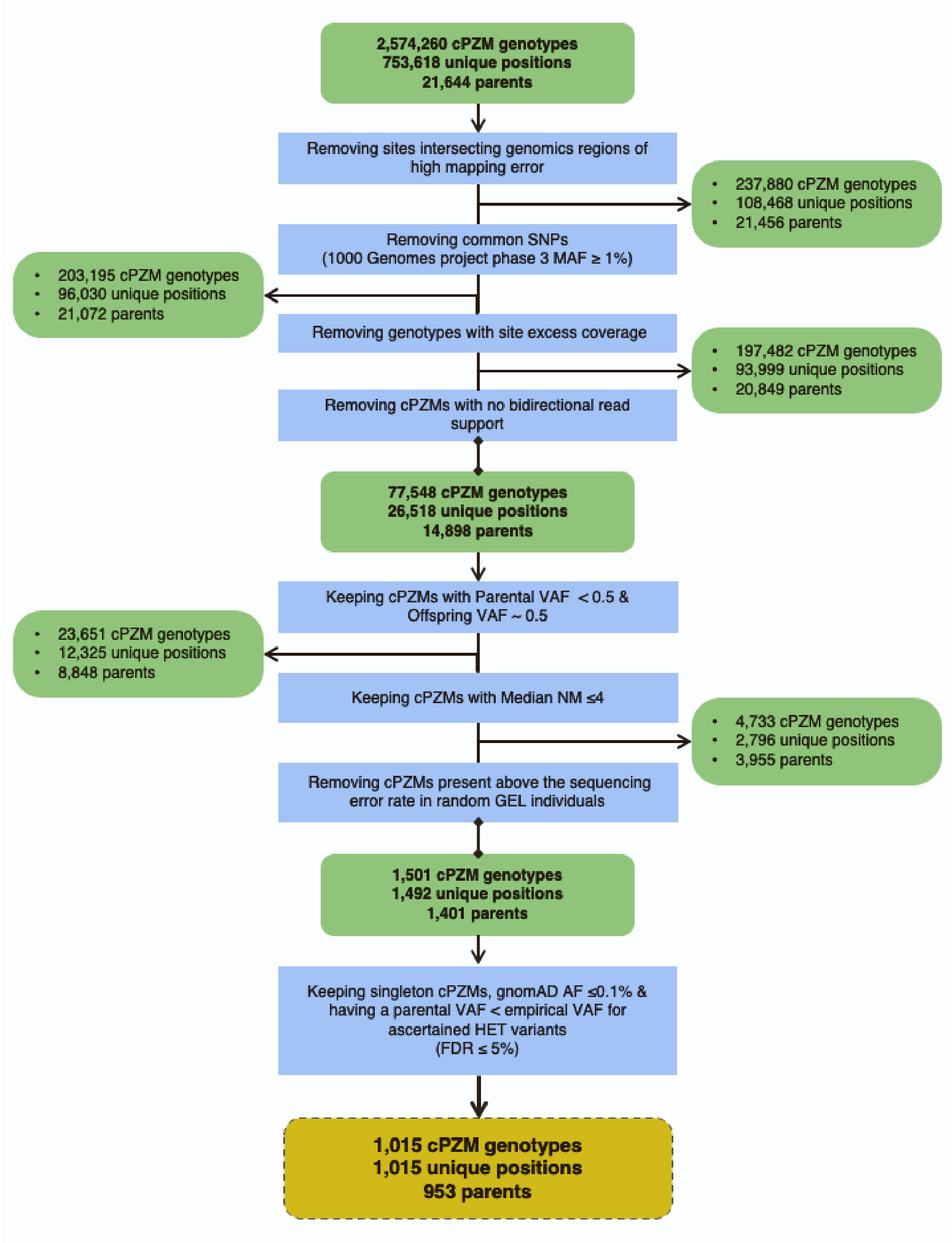

Figure S1. Summary of filtering steps for bioinformatic ascertainment of parental postzygotic mutation in the GEL family trio cohort.

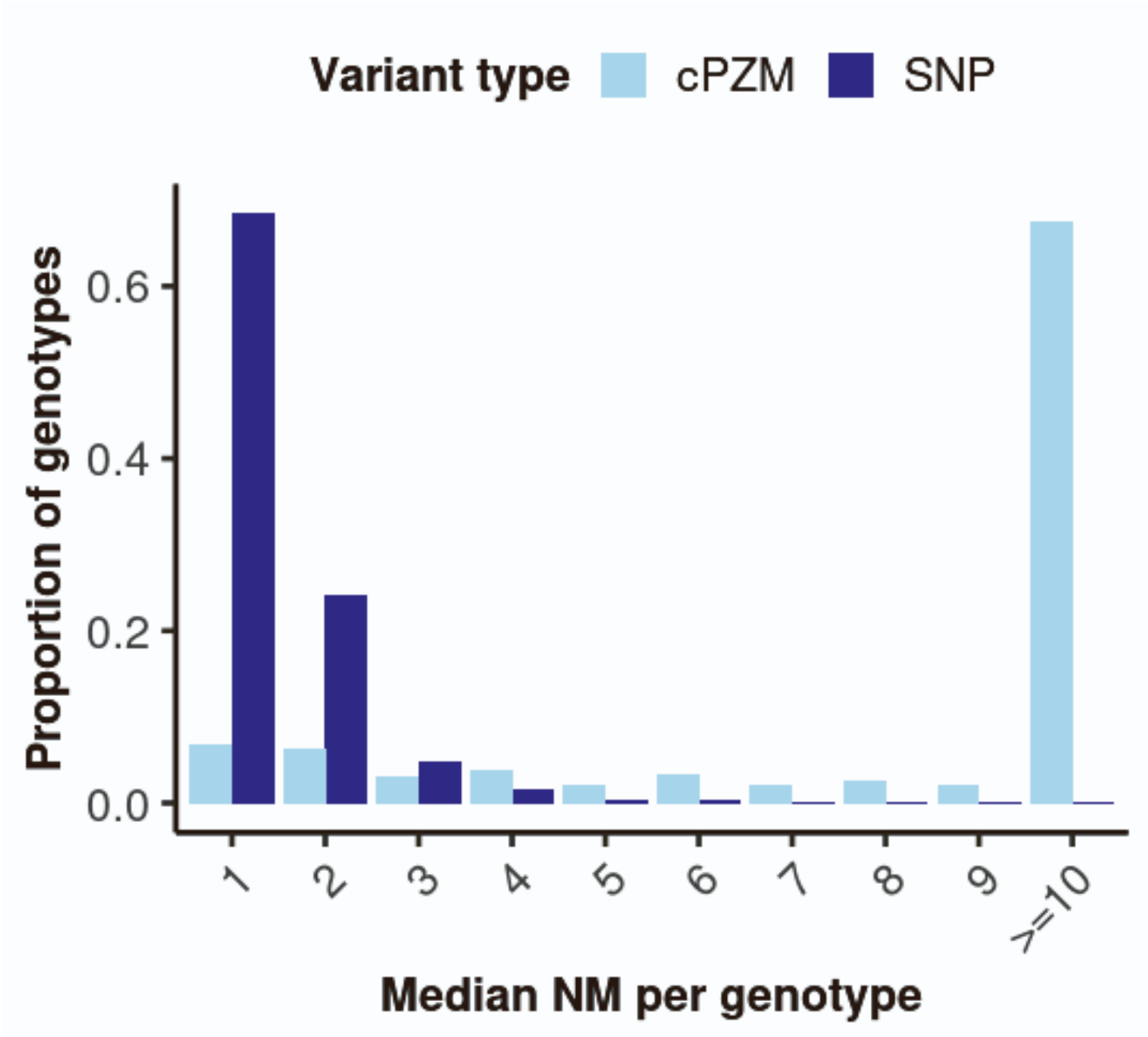

Figure S2. Distribution of median number of mismatches (NM) for reads mapping to cPZMs and GIAB SNPs. Showing proportions out of a total of 23,651 cPZMs passing filtering steps 1-5, and up to 4600 high quality SNPs.

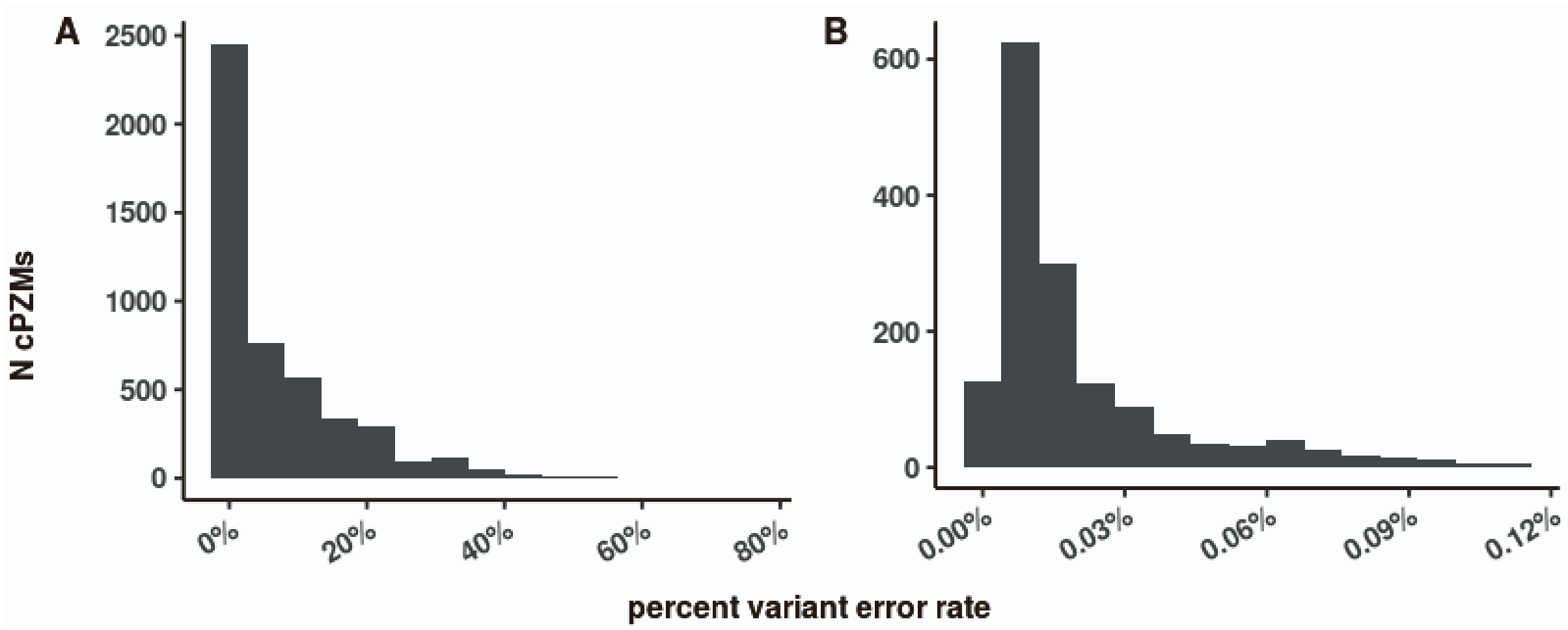

Figure S3. Variant-specific error rate distributions for cPZMs passing filtering steps 1-6. Showing variant specific error rates for 4,734 cPZMs **A.** passing filtering steps 1-6 **B.** passing filtering step 8 (n = 1,501). Error rate filtering threshold corresponds to the median Illumina HiSeq 2500 error rate (0.112%).

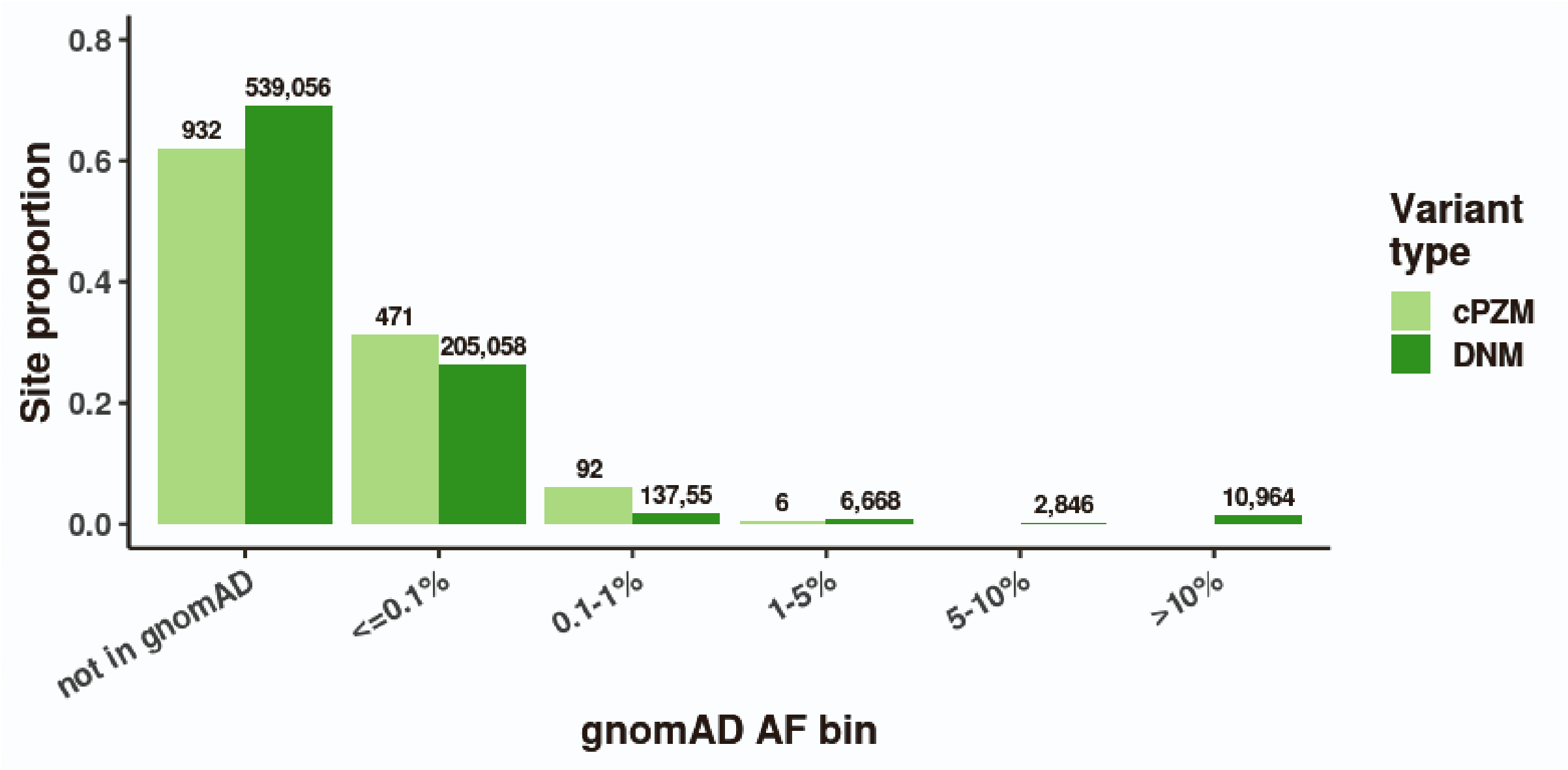

Figure S4. Distribution of population allele frequencies (gnomAD genomes v3). Showing binned gnomAD allele frequency distributions for 1,501 cPZMs remaining after step 1-7 filtering and filtered GEL DNMs per trio (stringent filter) reported by Kaplanis et al. [3]. Top numbers indicate counts per bin and variant type.

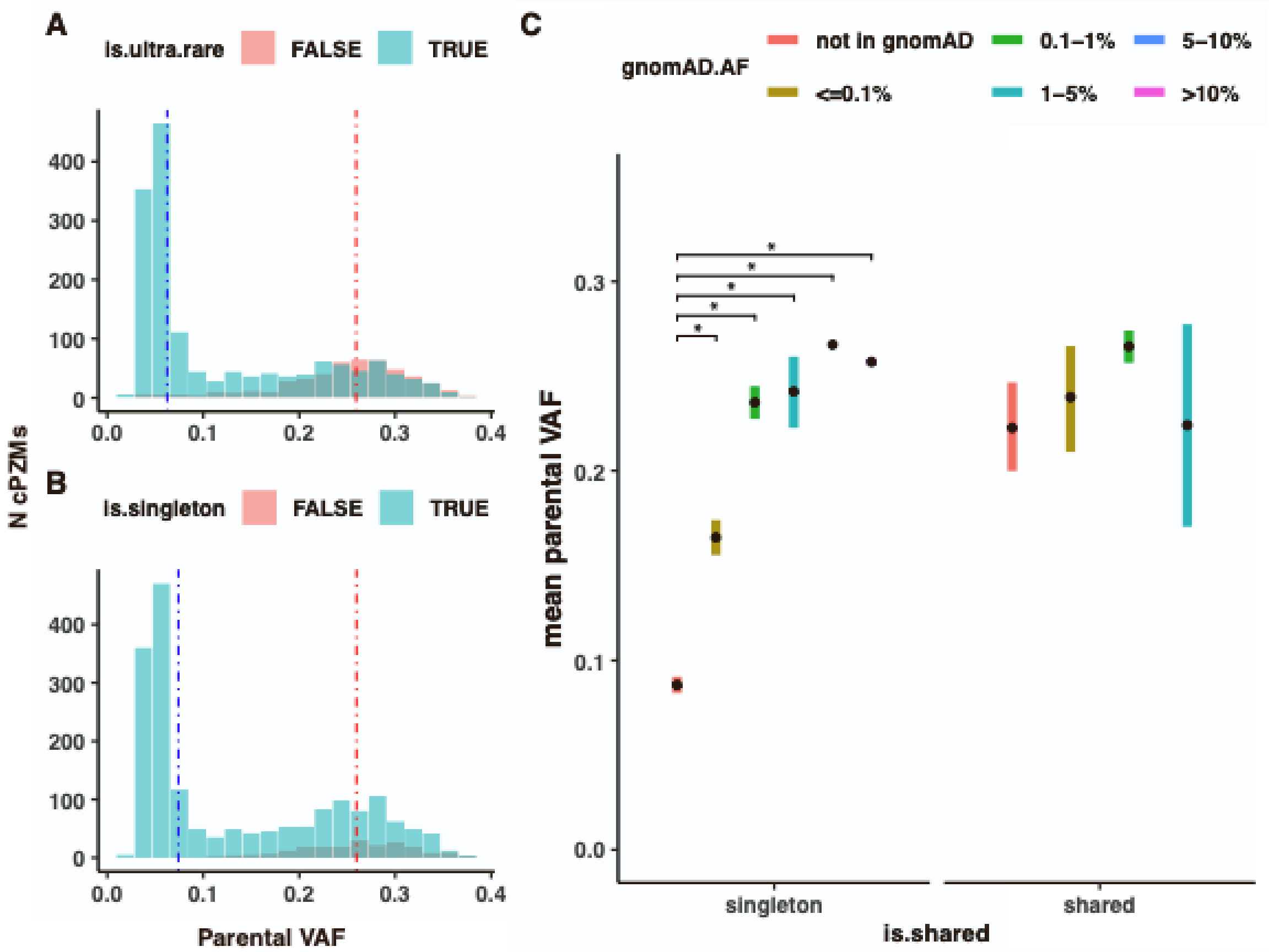

Figure S5. Parental VAF comparisons across population allele frequency bins for cPZMs passing filtering steps 1-7. A. Parental VAF of ultra-rare and non-ultra-rare cPZMs classes (n = 1,501). The ultra-rare group represents a composite between cPZMs absent from gnomAD or having a gnomAD AF ≤0.1%. **B.** Parental VAF of cPZMs found as singletons (i.e., found in a single parent) or shared across the 1,401 unrelated parents having at least one cPZM passing filtering steps 1-7. **C.** Mean parental VAFs for cPZMs stratified into six population allele frequency bins (gnomAD) and according to their presence across unrelated parents (i.e. singleton = seen in only one parent, shared = seen in one or more parent). Bars correspond to 95% confidence intervals for the mean estimate for each group. Horizontal lines and asterisks indicate significant VAF differences between groups (ANOVA and Tukey HSD post-hoc test adjusted p-values ≤ 0.05).

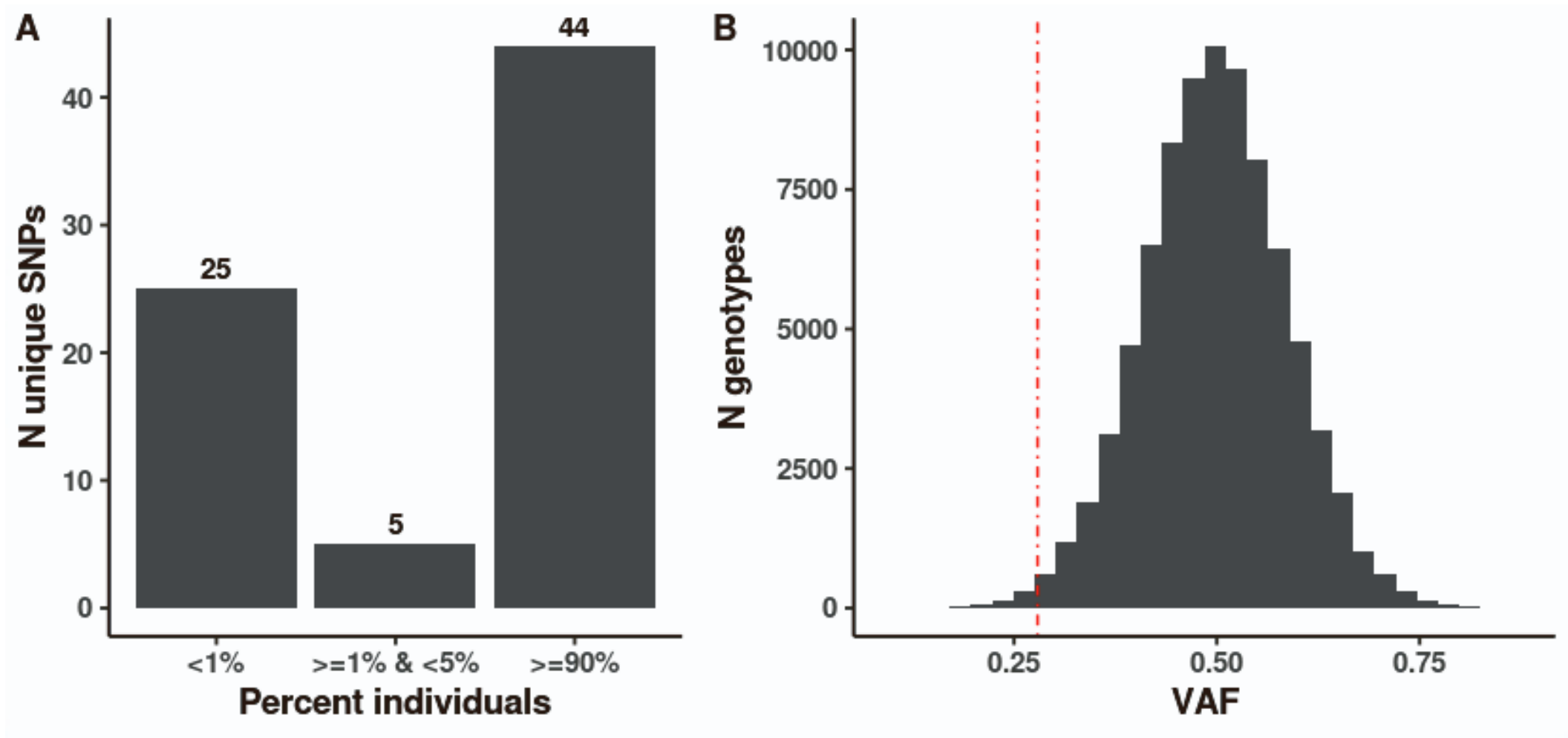

Figure S6. Description of SNPs selected to derive an empirical lower variant allele fraction (VAF) bound threshold for heterozygous (HET) variants. A. Number of HET SNPs shared across different proportions of the subcohort of interest (n = 1,293)**.** Top numbers indicate the number of individuals in each bar. **B.** VAF distribution for HET genotypes at the selected SNPs. The red line overlaps the mean lower-bound VAF threshold (mean VAF threshold = 0.27) across 1,293 individuals.

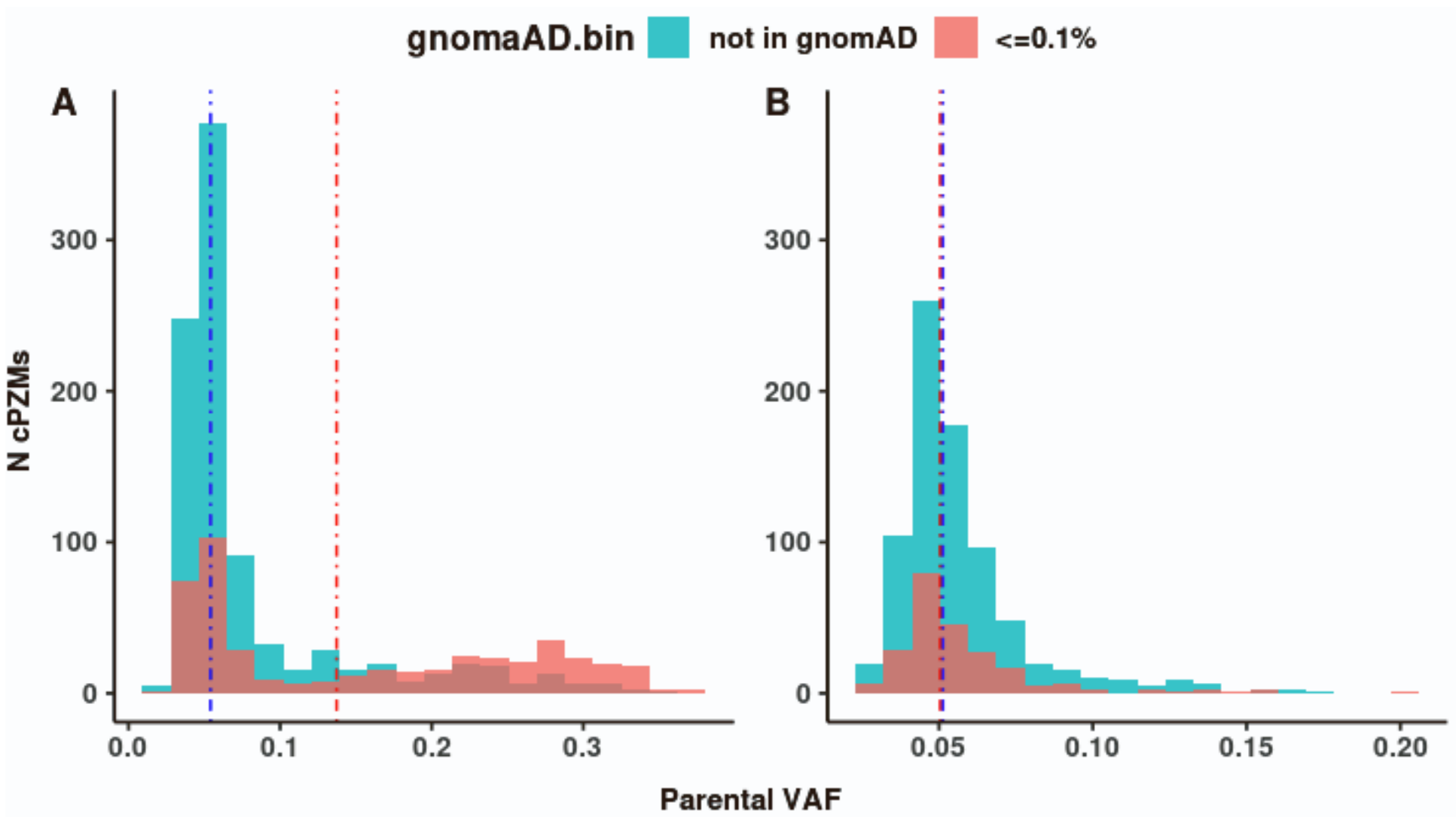

Figure S7. Parental VAF distribution comparisons for singleton cPZMs stratified by gnomAD allele frequency. A. All singleton cPZMs passing filtering steps 1-8 (n = 1,389). **B.** Singleton cPZMs after removing putative HET sites (filtering step 9; n = 1,015). Dotted lines intersect with median parental VAFs for cPZMs not found in gnomAD (blue), and found in gnomAD (red) in each panel. Median parental VAF is significantly different between groups before removing putative HETs (Wilcoxon rank-sum test p-value = 2.2×10^{-16}), and non-significant after filtering these sites (Wilcoxon rank-sum test p-value = 0.98).

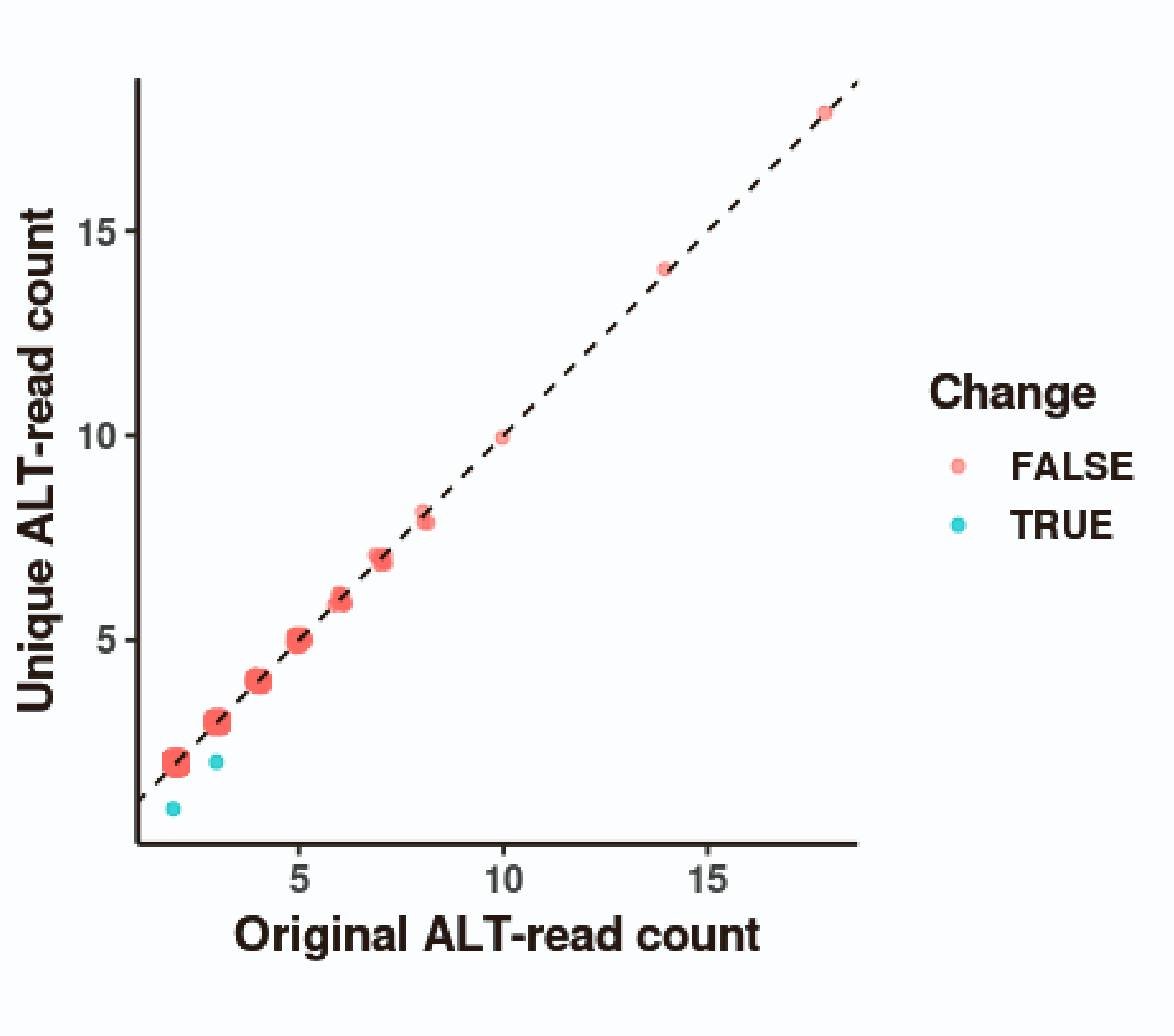

Figure S8. Sanity check for unique alternative allele read support for final PZM candidates. Showing the number of reads supporting the alternative (ALT) allele for all final PZM candidates (n = 1,015). For each PZM, the x-axis shows the alternative allele read support before subsetting to unique read names, while the y-axis shows the remaining read counts after keeping unique read names. Colors indicate whether there was reduction in the total ALT supporting reads after keeping unique read names.

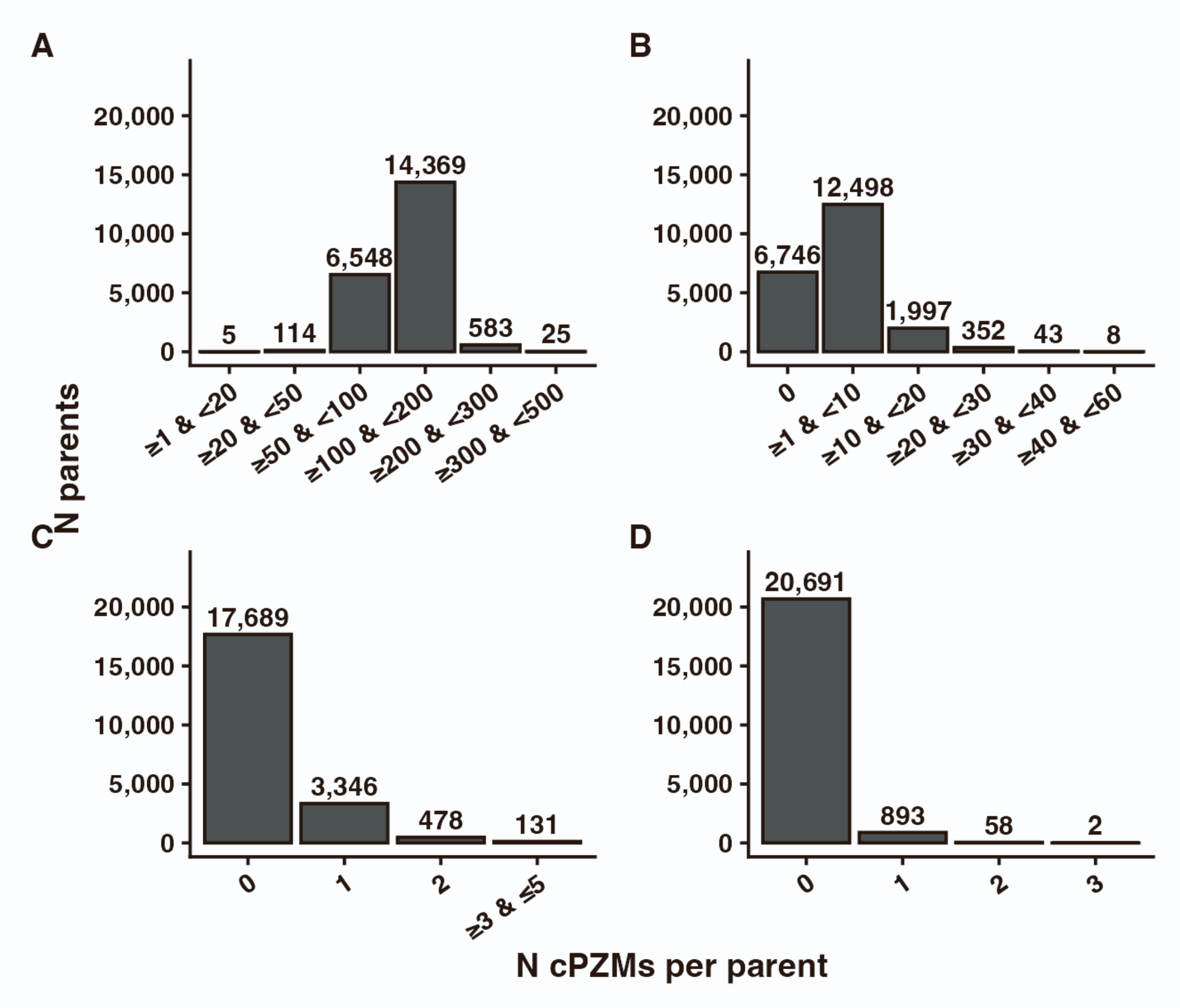

Figure S9. Number of cPZMs per parent across main filtering steps. Number of candidate cPZMs per individual across the whole initial cohort of 21,644 parents for: **A.** Initial selection of 2,574,260 cPZMs. **B.** 77,548 cPZMs remaining after removing variants intersecting genomic regions of high mapping error, reported in the 1kGP phase 3 (MAF ≥1%)[5], with a total site sequencing above individual sequencing-excess thresholds (as defined in filtering step 2 - **"**Excess coverage filter"), or supported by less than one read on each strand **C.** 1,501 cPZMs remaining after removing likely HET variants based on parental and offspring VAFs, having a median NM >4, or being above the median Illumina 2500 error rate in random GEL participants. **D.** 1,015 cPZMs remaining after removing variants with gnomAD AF >0.1%, shared across unrelated parents, or not being significantly different from the empirical lower-bound VAF threshold for ascertained HET variants (FDR >5%). Top numbers indicate counts per bin and variant type.

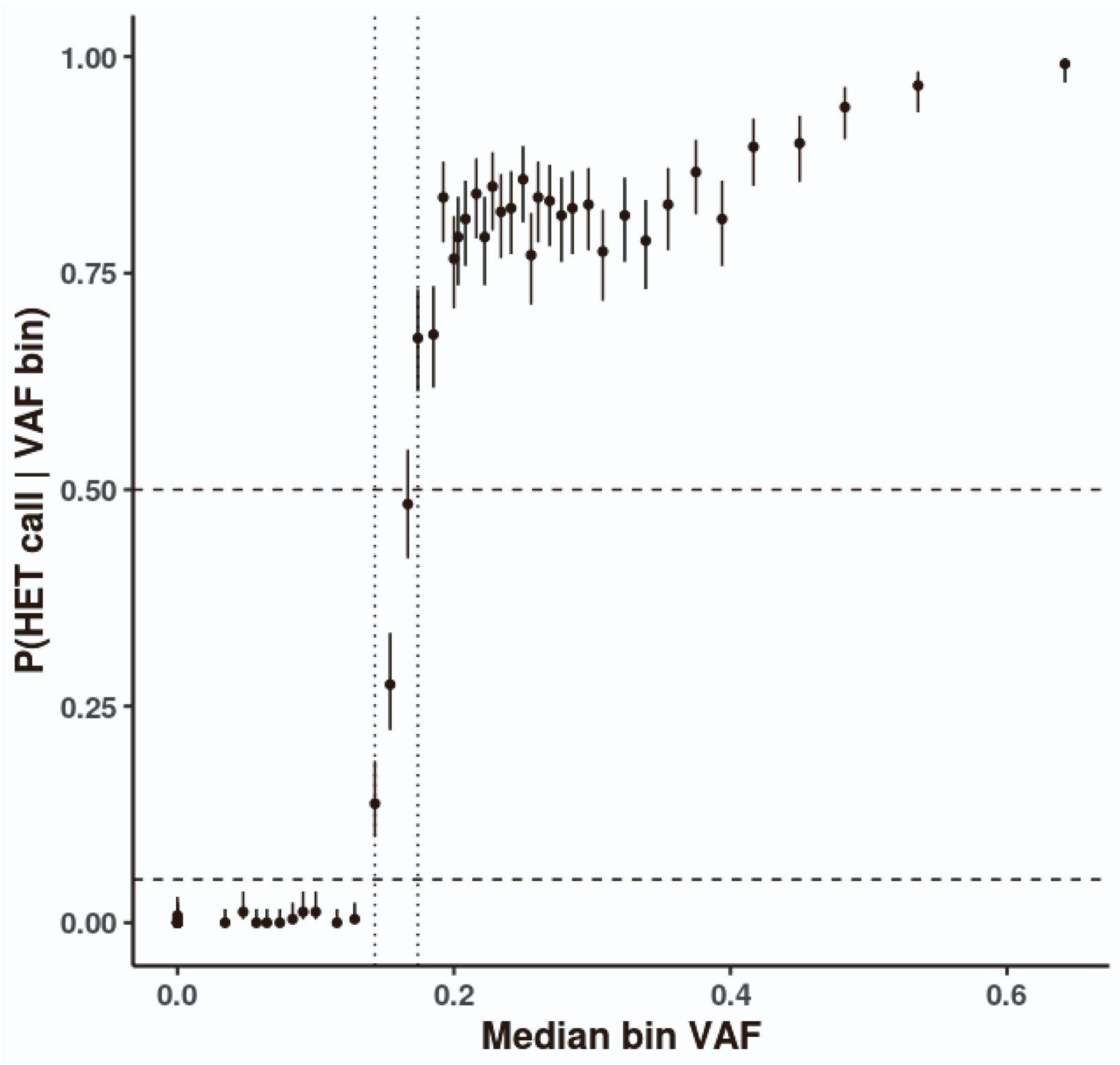

Figure S10. Platypus empirical VAF thresholds for HET genotype calling. Showing the empirical probability of HET calling by platypus across 50 VAF bins for 6,000 randomly selected Mendelian inconsistencies (12,000 genotypes across parents and offspring). Error bars are 95% Wilson score confidence intervals on the binomial proportion within each bin. Vertical dotted lines intersect VAF ≥ 0.14 and ≥ 0.17, where the probability of HET calling are 5% and 50%, respectively (dotted horizontal lines)

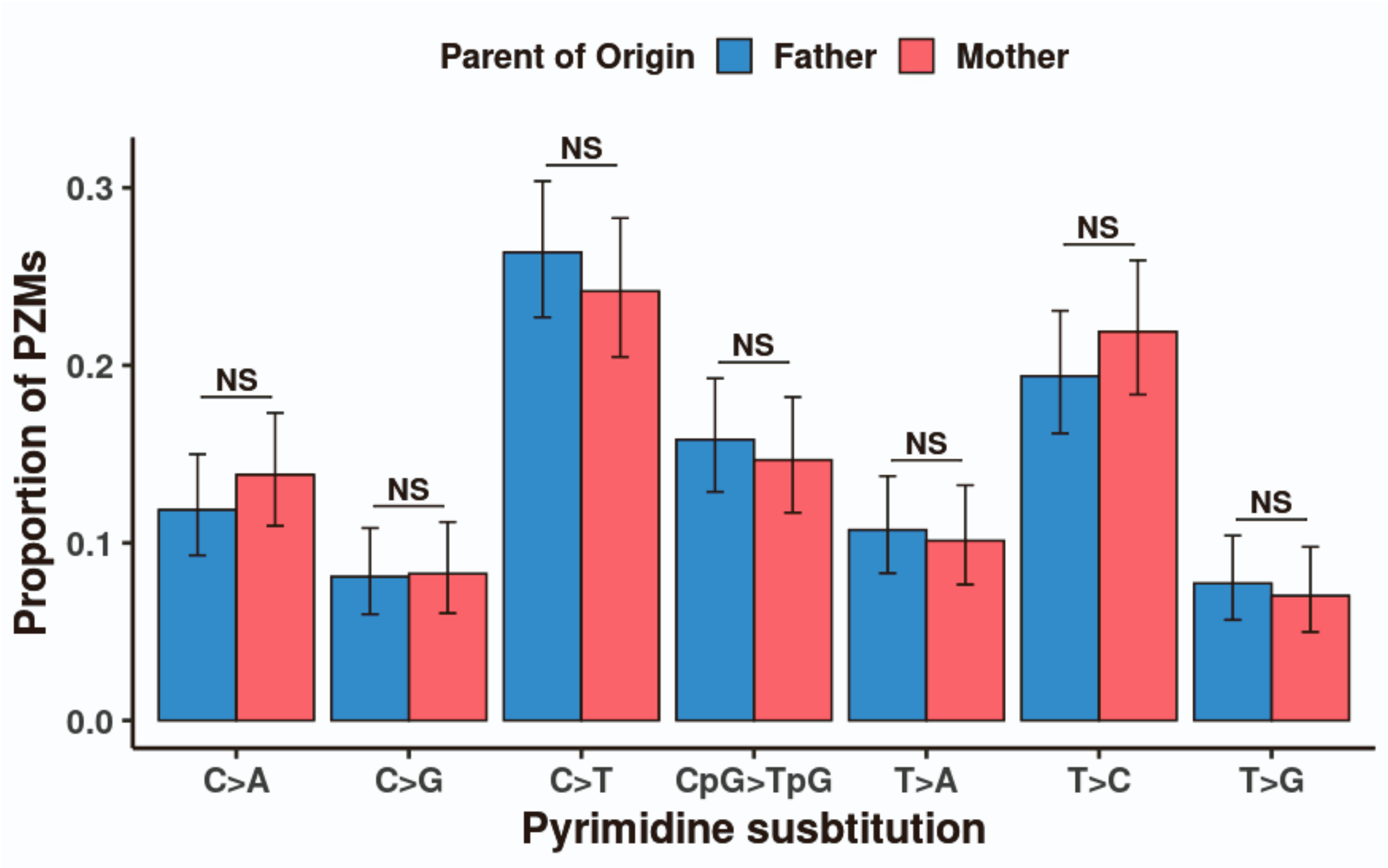

Figure S11. Spectral characteristics of PZMs stratified by sex. Bars represent 95% confidence intervals for grouped pyrimidine substitution proportion (one-sample proportion test with continuity correction).

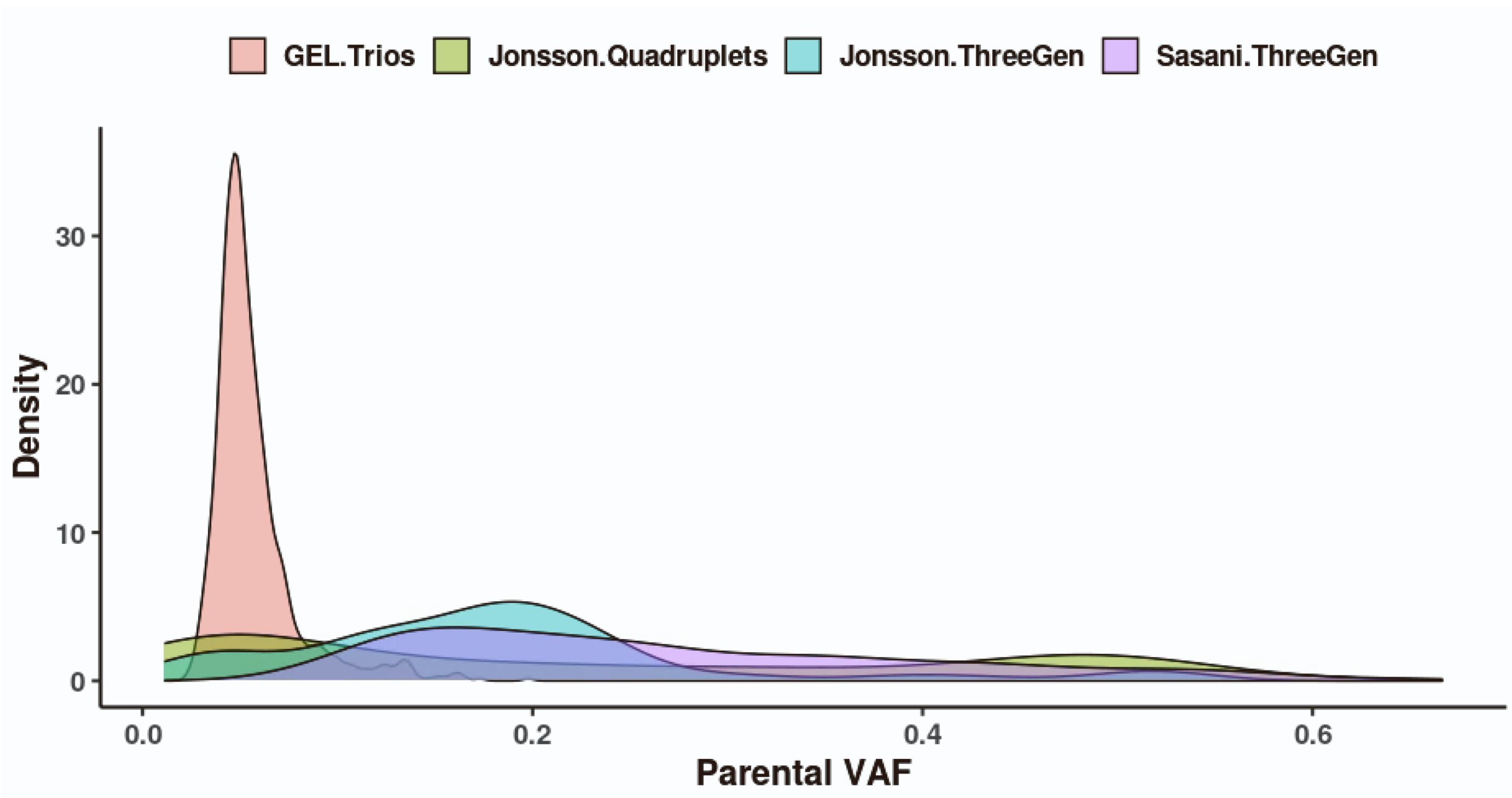

Figure S12. PZM VAF distributions stratified by study and PZM ascertainment method

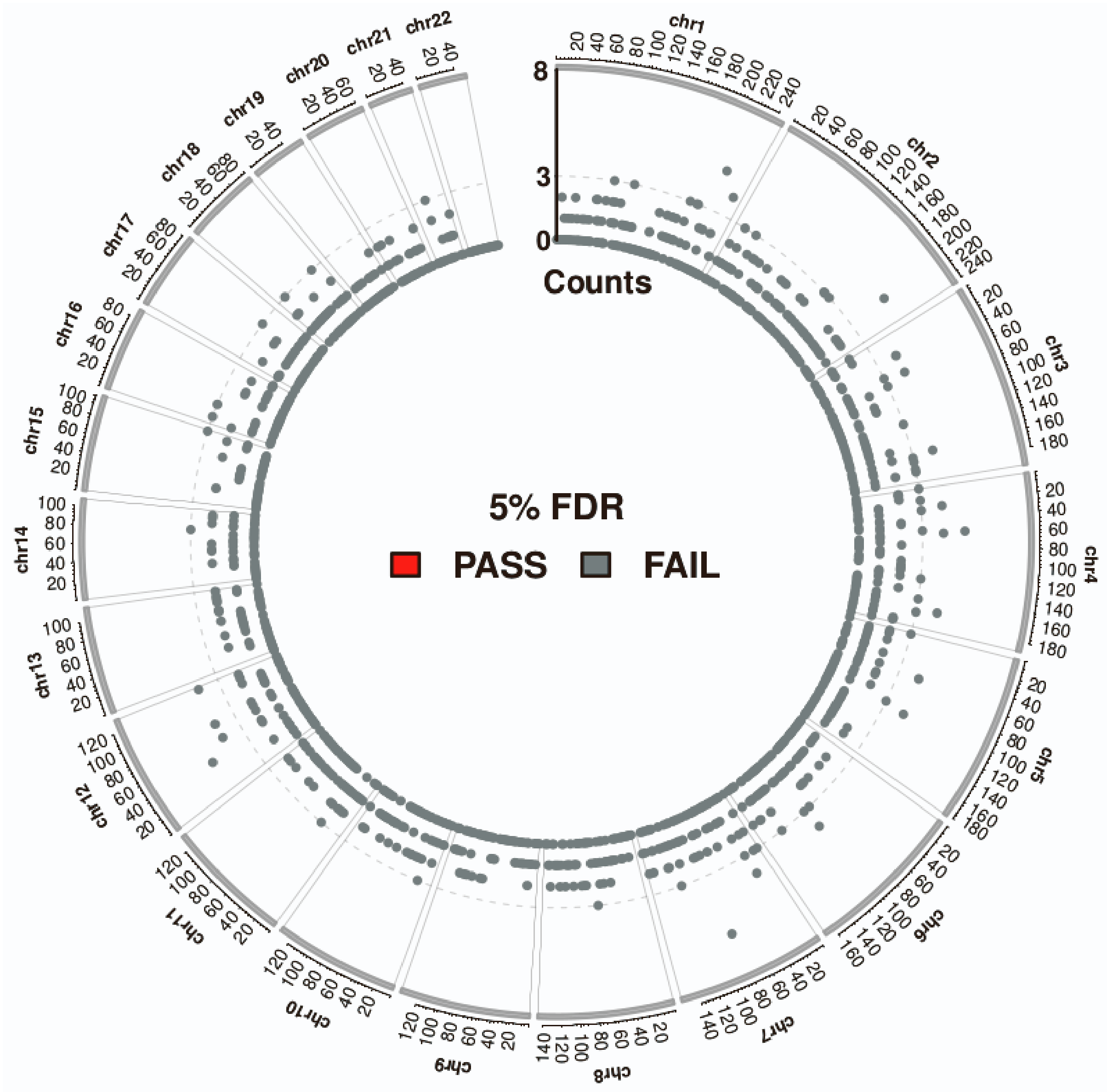

Figure 13. Genomic distribution of PZMs. Each point represents cohort-wide PZM counts for a given non-overlapping 2Mb window. No bin was found significantly enriched after multiple testing significance correction.

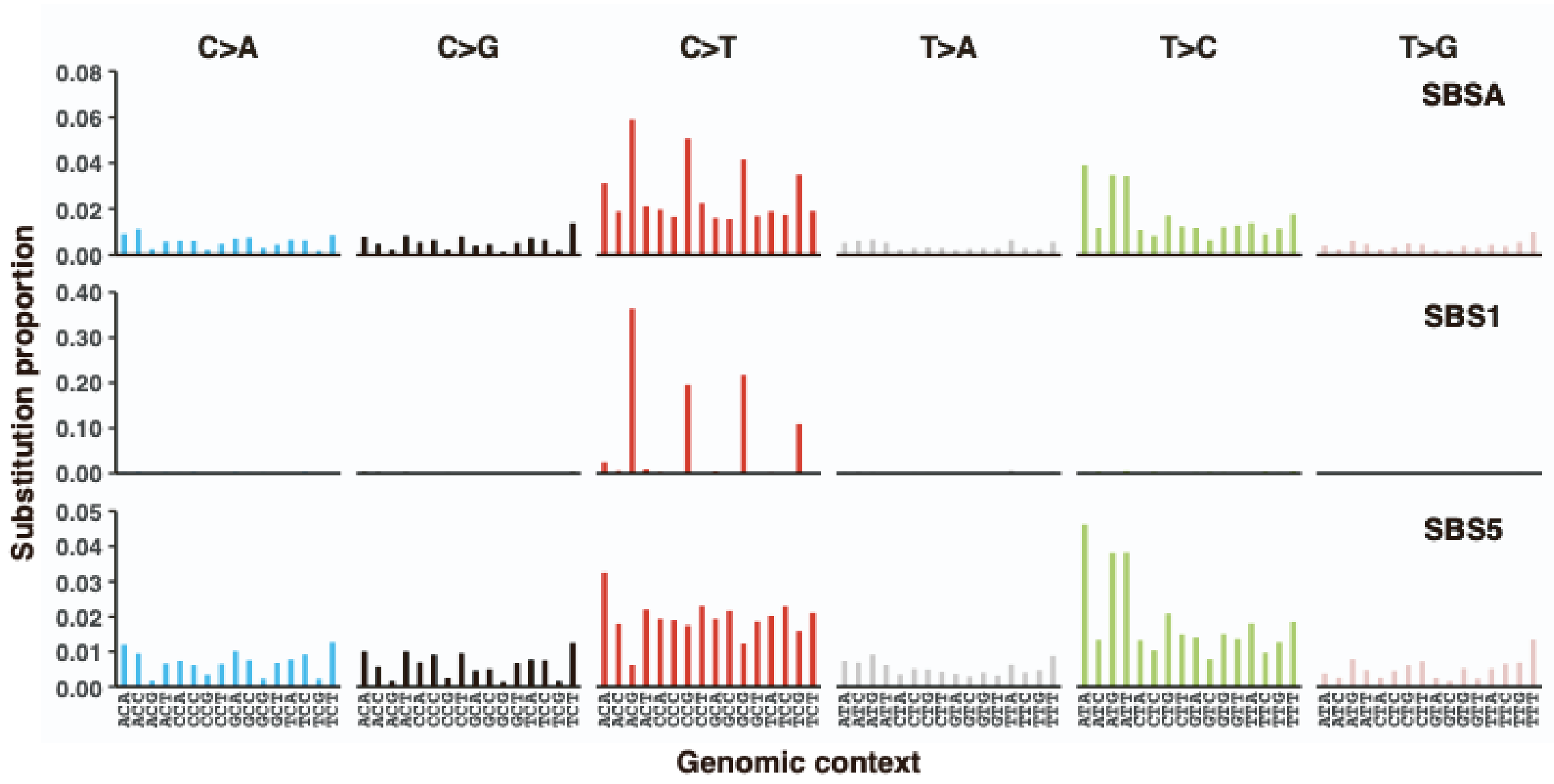

Figure S14. Mutational signature extraction and deconvolution of PZMs. Showing single nucleotide mutation proportions across the 16 possible 5’-3’ base-flanking contexts for (from top to bottom): SBSA (de novo extracted signature profile from GEL DNMs and PZMs), deconvoluted COSMIC SBS1, and and COSMIC SBS5 (cosine similarity to SBSA = 0.98).

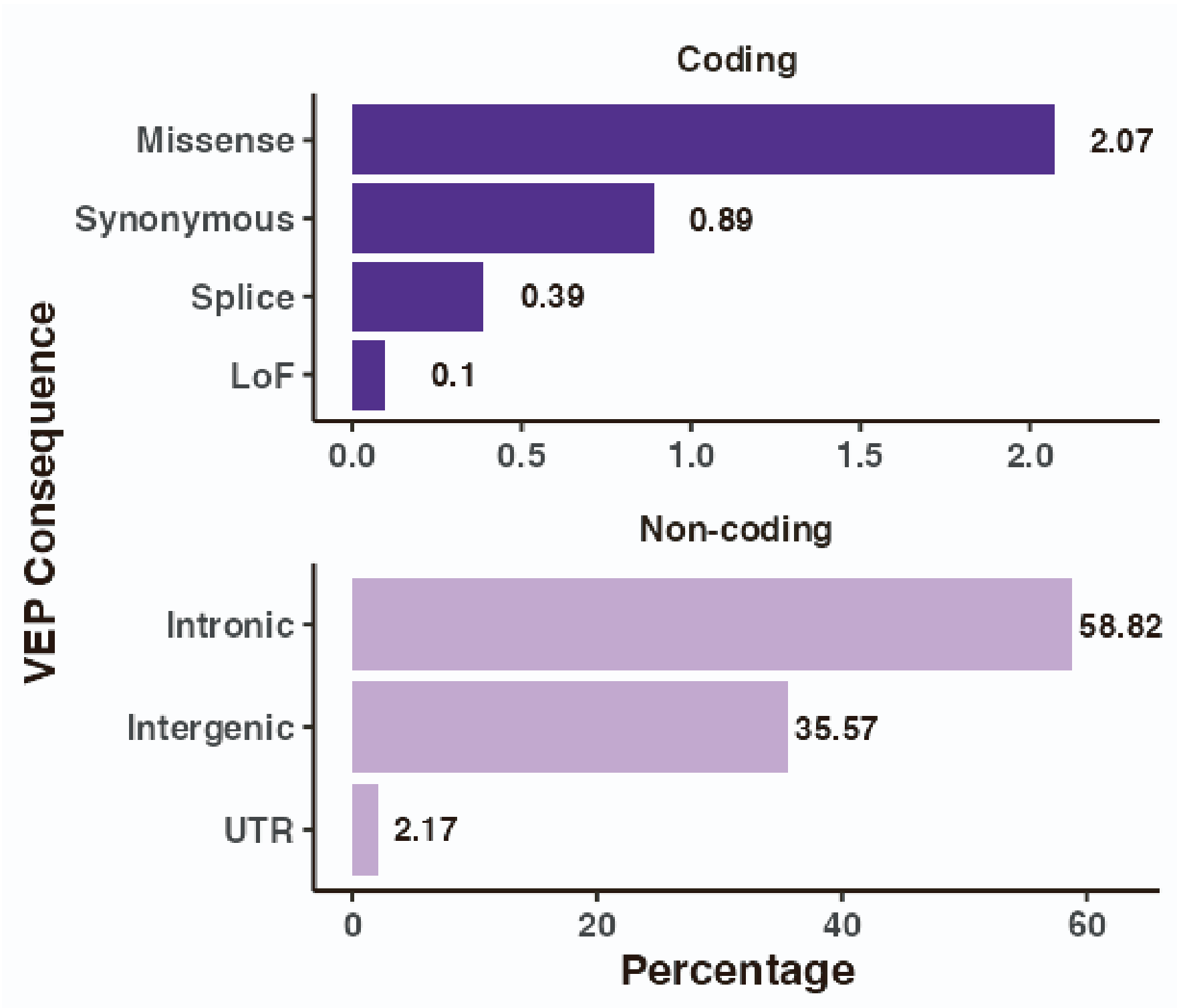

Figure S15. Consequence effect prediction for final cPZMs set (n = 1,015).

Supplemental tables

Embryonic cell division	Number of Cells	Expected cell proportion carrying the mutant allele	Expected variant allele fraction in parental somatic tissues
1	2	0.5	0.25
2	4	0.25	0.125
3	8	0.125	0.0625
4	16	0.0625	0.03125
5	32	0.03125	0.015625
6	64	0.015625	0.0078125
7	128	0.0078125	0.00390625
8	256	0.00390625	0.001953125
9	512	0.001953125	0.0009765625
10	1024	0.0009765625	0.00048828125

Table S1. Expected PZM VAFs and mutant cell proportions in adult tissues. Simplified model assuming synchronous and equal cell division, a stable diploid genome, no selection on mutant cells, and an even distribution of mutant cells across adult tissues for PZM events occurring between the 1st and 10th cell division.

Study design [Publication]	Quadruplets [Jonsson et al., 2021]	Three Generation [Jonsson et al., 2021]	Sibling shared DNMs [Sasani et al., 2019]	Three Generation [Sasani et al., 2019]	Simple trios [GEL]
Median Parental Somatic VAF [VAF range]	0.173 [0.01, 0.63]	0.176 [0.02, 0.53]	NA*	0.241 [0.10, 0.67]	0.049 [0.02,0.34]
Pyrimidine Substitution	Pyrimidine Proportion Odds Ratio (PZMs/DNMs) [95% confidence intervals]				
C>A	1.55 [1.17, 2.02]*	1.13 [0.50, 2.23]	1.55 [1.22, 1.95]*	1.22 [0.88, 1.67]	1.34 [1.11, 1.61]*
C>G	0.62 [0.42, 0.89]*	0.50 [0.16, 1.21]	0.70 [0.49, 0.96]*	0.88 [0.60, 1.27]	0.86 [0.68, 1.08]
C>T	1.24 [1.04, 1.49]*	1.26 [0.82, 1.89]	0.87 [0.71, 1.05]	1.02 [0.81, 1.27]	1.06 [0.92, 1.22]
CpG>TpG	0.78 [0.61, 0.99]*	0.30 [0.11, 0.67]*	1.55 [1.30, 1.85]*	0.85 [0.64, 1.11]	0.85 [0.71, 1.01]
T>A	1.15 [0.82, 1.58]	1.53 [0.74, 2.86]	0.73 [0.49, 1.06]	1.58 [1.11, 2.22]*	1.67 [1.35, 2.04]*
T>C	0.68 [0.54, 0.86]*	1.24 [0.81, 1.87]	0.79 [0.65, 0.96]*	0.88 [0.69, 1.11]	0.79 [0.67, 0.91]*
T>G	1.97 [1.42, 2.67]*	1.27 [0.46, 2.88]	0.67 [0.46, 0.95]*	1.06 [0.73, 1.53]	1.11 [0.86, 1.40]
Total SNV PZMs	529	102	720	442	1,015

Table S2. Differences in mutation spectra between PZMs and DNMs. Fisher's exact test odds ratios for pyrimidine substitution count differences across PZMs and DNMs identified by Sasani et al., 2019, Jonsson et al., 2021 and the Genomics England cohort. PZM counts reported by Sasani and Jonsson were stratified by the extended family trio strategy used to identify PZMs. The most likely enrichment of parental VAF for each ascertainment group is also shown. Asterisks indicate significant differences (PZM/DNMs proportions) after accounting for multiple testing correction in each PZM group (FDR≤5%).

NC_000017.11:g.8161693C>G (*VAMP2*)<br>Pathogenic (class 5, posterior probability: 0.994)	
PM2 applied (moderate)	Not present in gnomAD with adequate coverage
PS1 applied (strong)	Applied on the basis of a quality case report (PMID 37901860[42]) with the same amino acid change in a de novo state in the proband, and one Clinvar pathogenic submission from GeneDx.
PP2 applied (supporting)	Missense variants in gene are a common mechanism of disease
PP3 applied (supporting)	REVEL score: 0.628 intermediate (< 0.7); CADD score: 31 deleterious (≥ 30); SpliceAI score: 0 tolerated (≤ 0.2)
PS2 applied (moderate)	Applied at reduced strength, moderate level
NC_000014.9:g.101985930C>T (*DYNC1H1*)<br>Likely pathogenic (class 4, posterior probability: 0.949)	
PM2 applied (moderate)	Not present in gnomAD with adequate coverage
PS1 applied (moderate)	Applied on the basis of a quality case report (PMID 38953796[43]) with the same amino acid change in a de novo state in the proband; Clinvar evidence was deemed insufficient for PS1 for this variant.
PP2 applied (supporting)	Missense variants in gene are a common mechanism of disease
PP3 and BP7 not applied	REVEL score: 0.523 intermediate (< 0.7); CADD score: 26.2 intermediate (< 30); SpliceAI score: 0.01 tolerated (≤ 0.2)
PS2 applied (moderate)	Applied at reduced strength, moderate level
NC_000011.10:g.32389172C>A (*WT1*)<br>VUS (class 3, posterior probability: 0.812)	
PM2 applied (moderate)	Not present in gnomAD with adequate coverage
PP2 applied (supporting)	Missense variants in gene are a common mechanism of disease
PP3 and BP7 not applied	REVEL score: 0.311 intermediate (< 0.7); CADD score: 24.6 intermediate (< 30); SpliceAI score: 0.02 tolerated (≤ 0.2)
PS2 applied (moderate)	Applied at reduced strength, moderate level

Table S3. ACMG classification of parental PZMs with potential diagnostic relevance.

Supplemental materials and methods

Section 1. Identification of candidate parental postzygotic mutations

We accessed a comprehensive set of 14,446,487 unfiltered Mendelian inconsistencies, generated using the Platypus variant caller[1] from blood whole genome sequencing (WGS) data across 13,928 family trios, consisting of both parents and one offspring[2]. Variant calling was performed on a per trio basis, with each inconsistency representing a site where the offspring is heterozygous (HET) for the alternative (ALT) allele while both parents are homozygous for the reference (REF) allele. When multiple offspring were recruited for a given family, variant calling was performed separately for each offspring, meaning a given site could be flagged multiple times across siblings.

From this initial set, we retained only autosomal (chr1-22) single nucleotide variants (SNVs; A, T, G, C alleles) called on GRCh38-mapped genomes (n = 5,739,417). We excluded all trios previously identified to have biological or technical sources of germline hypermutation[3] yielding 5,720,018 SNVs across 11,996 trios, involving 21,644 unique parents.

From these, we excluded 1,473,685 genotypes with zero ALT reads in both parents, reasoning that these are consistent with true *de novo* germline mutations transmitted to the child rather than parental PZMs, keeping 4,246,333 genotypes . For each trio, we excluded genotypes where the ALT allele had any coverage (>0 reads) in both parents (n = 1,628,565), retaining 2,627,735 genotypes that were considered candidate postzygotic mutations (cPZMs) for downstream filtering.

Additionally, because a single family could contribute multiple trios when parents had more than one sequenced offspring, the same candidate parental PZM could be detected repeatedly via transmission to different children. Since our analyses focus on parental mosaicism rather than transmission counts, we de-duplicated these cases by retaining a single record for each unique parent–variant combination. After this step, we retained

2,574,260 cPZM genotypes spanning 753,618 unique genomic positions across 21,644 parents (mean = 118.93, Ts/Tv = 1.48).

Section 2. Bioinformatic filtering of candidate parental postzygotic mutations

We applied a rigorous, multi-step filtering pipeline to curate a high confidence set of candidate parental postzygotic mutations (**Figure S1**). Although 21,644 unique parents were originally considered, a total of 123 were removed due to missing individual or trio-level metadata. Hence, mean cPZMs per person were calculated based on a total of 21,521 parents.

1. **Removal of problematic genomic regions:** We excluded variants overlapping genomic regions prone to mapping errors, including interspersed repeats, low complexity regions (e.g. satellite repeats), centromeres, segmental duplications, alternative haplotypes, and genome assembly patches. These regions were identified from the GRCh38 RepeatMasker, Segmental Dups, Simple Repeats, GRC Patches and alternative haplotypes (fixSeqLiftOverPsl; altSeqLiftOverPsl), and Centromeres tracks from the UCSC table browser[4]. Assuming that all the bases per chromosome in the GRCh38 genome assembly (as determined in the UCSC hg38.chrom.sizes file) are callable, the removal of these regions means that our dataset is only powered to nominate PZM candidates in ~43% of the autosomal human genome. This step reduced the dataset to 237,880 cPZM genotypes over 108,468 unique positions, across 21,456 parents (mean = 11.05, Ts/Tv = 0.86).

2. **Filtering common variants:** We removed variants with a global minor allele frequency (MAF) ≥ 1% in the 1000 Genomes Project (Phase 3)[5], resulting in 203,195 cPZM genotypes at 96,030 unique positions across 21,072 parents (mean = 9.44, Ts/Tv = 0.77).

3. **Excess coverage filter:** To remove potential sequencing artefacts, we excluded variants with site depth above an individual-specific upper threshold representing the

top 1% of the expected coverage distribution, calculated using $qpois()$ in R ($lambda$ = mean whole-genome coverage; $lower.tail = FALSE$). We also removed 1,097 cPZMs from 117 parents with missing sequencing coverage metadata. After filtering 197,482 cPZM genotypes at 93,999 sites remained across 20,849 parents (mean = 9.17, Ts/Tv = 0.75).

4. **Minimum read coverage support filter:** Using $bamreadcount$[6], we calculated strand-specific ALT allele coverage for each cPZM and retained only variants with at least one ALT read on both strands. This yielded 77,548 cPZMs at 26,518 positions across 14,898 parents (mean = 3.60; Ts/Tv = 0.69). Notably, 7,120 cPZMs had been assigned ALT = 1 in Platypus due to stringent filtering on windowed read support (see $bad - reads$ parameter[7]). Because the GEL *de novo* filtering pipeline allows candidate DNMs to have a maximum parental ALT allele count of one, these variants were thus misclassified as candidate DNMs and kept in further filtering steps of that pipeline.

5. **Variant allele fraction (VAF) consistency filter:** we used $binom.test()$ in R to assess whether parental VAFs were significantly <0.5 (one-sided test), and offspring VAFs consistent with 0.5 (two-sided test). We excluded variants failing either test (p >0.05 for parental VAFs, and p ≤0.05 for offspring VAFs), and 26 cPZMs from 6 parents with missing offspring read data. This retained 23,651 cPZMs at 12,325 unique positions across 8,848 parents (mean = 1.09, Ts/Tv = 0.77).

6. **Read quality based on edit distance filter:** We calculated the median edit distance (NM tag) of ALT supporting reads using $samtools\ view$ and $samjdk.jar$[8]. Reads with mapping quality <20 or matching the bitflag (--excl-flags) 3844 (i.e., unmapped reads, not primary alignments, reads failing vendor quality checks, optical and PCR duplicates, and supplementary alignments) were excluded (**Figure S2**). We kept variants with median NM ≤4, yielding 4,733 cPZMs at 2,796 sites across 3,955

individuals (mean = 0.21, Ts/Tv = 1.43). Details on the derivation of the NM threshold are described in **Supplemental Methods - Section 3**.

7. **Variant-specific error filter:** To identify and exclude error-prone genomic positions, we estimated the background allelic error rate by pooling reads from 2,500 randomly selected unrelated parents at genomic sites harboring candidate PZMs (**Supplemental Methods - Section 4; Figure S3**). This background alternative allele (ALT) rate reflects the expected probability of observing a specific non-reference allele at a given genomic position under sequencing noise, shared technical artefacts, or population common variation. Importantly, this metric is distinct from a sample-level variant allele fraction and is expected to be close to zero for a real mutation event. For each position, we calculated the proportion of pooled reads supporting each nucleotide, and excluded variants showing a background ALT rate >0.00112, corresponding to the Illumina HiSeq 2500 error rate[9]). Such positions are likely to represent systematic sequencing errors or common variation rather than true low-level mosaicism This step retained 1,501 cPZMs at 1,492 sites across 1,401 parents (mean = 0.069, Ts/Tv = 1.68).

8. **Population frequency-based filtering:** We annotated the remaining cPZMs with their internal GEL cohort and gnomAD v3 population frequencies[10]. Only 9/1,501 variants were shared across multiple unrelated GEL parents. For external AFs, we found that 569/1,501 cPZMs (50.4%) were present in gnomAD. Only 98 of these variants (6.5%) were present at AFs >0.1%. To set an expectation of population allele frequency for *de novo* variation, we compared the gnomAD AFs from cPZMs with those from 778,347 filtered DNMs from the GEL cohort, previously reported by Kaplanis et al., 2022[24]. We observed that ~69% of DNMs were absent from gnomAD and 26% were present at AFs ≤0.1% (**Figure S4**). However, we found significant differences between the proportion of variants absent from gnomAD and those present in gnomAD at AFs ≤0.1% between DNMs and cPZMs (single sample

proportion test, p-value = 2.18×10^{-09}, and 2.18×10^{-05} , respectively), indicating possible contamination of cPZMs with inherited HET variation.

To test for contamination by inherited HETs or sequencing errors, we compared VAFs for cPZMs across allele frequency bins. Variants absent from gnomAD or with AFs ≤0.1% had significantly lower VAFs than common variants (Wilcoxon rank-sum test, p = 2.2×10^{-16} ; **Figure S5A**). cPZMs shared by multiple parents in the dataset also had higher VAFs than cPZMs seen in just one parent (which we refer to as 'singletons') (Wilcoxon rank-sum test, p = 3.23×10^{-07} ; **Figure S5B**). Despite the ultra-rare group showing the lowest parental VAFs, the mean VAF of singleton variants absent from gnomAD was significantly lower than that of variants present at any AF in gnomAD (one way ANOVA and Tukey HSD post-hoc testing adjusted p-value ≤0.05; **Figure S5C**). In contrast, among cPZMs shared across unrelated individuals in the GEL cohort, there was no difference in mean VAF between those absent from gnomAD versus present in any of the tested allele frequency bins in gnomAD (one way ANOVA and Tukey HSD post-hoc testing adjusted p-value > 0.05; **Figure S5C**).

Based on the described observations, and to prevent the inclusion of putative HET variants, we first removed variants having a gnomAD AF >0.1% or shared among unrelated parents, retaining 1,389 cPZMs across 1,302 parents (mean = 0.064, Ts/Tv = 1.60).

9. **Empirical HET VAF threshold-based filter:** To remove putative heterozygous variants, we estimated an individual-specific lower VAF bound for true germline HET sites using parental sequencing data. These sites were used as internal controls to characterise the distribution of VAF expected for *bona fide* heterozygous variants under each individual's sequencing depth and technical noise.

To do this, we started by obtaining germline genotypes for parents with at least one singleton cPZM (n = 1,302) from aggregated germline variant calls (AggV2) across 78,195 GEL participants in plink .pgen format, processed as described by Kousathanas et al. (2022)[40]. Genotypes were available for 1,293 individuals, as not all GEL participants were included in the aggregated dataset[41]. Using plink2, we generated an A-transposed genotype matrix (.traw) for SNPs with allele frequency between 0.2 and 0.8, identified sites called heterozygous (0/1) in at least one individual, and counted their occurrences among the 1,293 individuals.

Because no single SNP is heterozygous in all individuals, we prioritised variants that were most commonly observed in the heterozygous state across the subcohort, in order to maximise overlap while retaining sufficient SNPs per individual. For each individual, we selected two SNPs per autosome (44 SNPs total), prioritising those most common in the subcohort. This resulted in 74 unique SNPs overall, of which at least 44 were shared by ≥90% of individuals (**Figure S6A**).

We calculated VAF for each SNP using $bamreadcount$[6] (minimum base and mapping quality 20) and defined the lower bound for HET SNPs as 2.5 times the standard deviation of VAF across all SNPs in that individual (**Figure S6B**). For nine individuals lacking SNP data, we used the mean threshold from the others (mean = 0.27).

We then applied this threshold to the 1,389 remaining cPZMs, testing whether their VAFs fell significantly below the individual-specific lower bound using a one-sided $binom.test()$ (**Figure S7A**). Variants with FDR-adjusted p-value >0.05 were excluded, leaving 1,015 high-confidence cPZMs (**Figure S7B**) across 953 parents (mean VAF = 0.046, Ts/Tv = 1.57). No significant difference in parental VAFs was observed between candidates absent from versus present in gnomAD (Wilcoxon rank-sum test, p = 0.98).

To ensure that the final PZM candidate set was not likely dominated by variants called from the same DNA fragment we applied the sanity check described in **Section 5 (Figure S8)**. A summary of the number of cPZMs per individual found across the whole cohort throughout the main filtering steps in this pipeline is shown in **Figure S9**. Our final PZM catalogue had a Ts/Tv ratio of 1.57, which is lower than that expected from WGS variant calling (2-3[11]). This ratio is similar to that estimated for early PZMs reported by Sassani et al., 2019 (Ts/Tv = 1.6)[12], which is consistent with reports showing that somatic mutation catalogues are expected to have a lower Ts/Tv ratios due to different cell selection processes operating in the relevant tissues[13]. Importantly, active and passive selection processes are known to occur prior to the differentiation of the epiblast[14], where PGCs originate[15].

Section 3. Estimating an empirical maximum NM value for single nucleotide variants in GEL data

The NM metric, which is produced during the alignment step of sequencing and can be extracted from the CIGAR string, represents the number of mismatches per read[16]. To calibrate a hard threshold for median NMs in cPZMs, we extracted these values from a set of high quality common polymorphic sites. Specifically, we extracted median NMs for a set of common SNPs overlapping with high-quality mapping regions of the human genome (GRCh38 reference) from parents carrying cPZMs. To do this, from the gnomAD v3.1.1 variant set[10] and for each autosomal chromosome (chr1-22), we extracted a random selection of 1000 bi-allelic SNPs (i.e. min and max alleles = 2), which we then intersected with high-confidence mapping coordinates produced by the Illumina's Platinum Genomes Project[17,18]. These genomic regions are known to produce consistent variant calls, as these have been validated through consistent inheritance patterns across a three-generation pedigree[17,18]. To retain only common SNPs likely to be shared by most individuals regardless of ancestry, we set a filter on gnomAD global allele frequency ≥ 0.8.

As the purpose of calculating the median NM per variant is to approximate an expected number of mismatches in reads carrying the ALT allele in short read sequencing data, high quality SNPs obtained from previous steps were first compared against genotypes of the *de novo* cohort trio parents (n parents = 15,125). We checked that the alternate allele in the selected SNPs was found in heterozygous or homozygous form in at least 80% of the cPZM carriers. This was done using readily available aggregate VCF files containing filtered germline variant calls from 78,195 participants of the 100,000 genomes project[19], which includes all of the family trio parents studied in this section. Most of the selected SNPs were at least heterozygous in 80% of the family trio parents (n ~ 900 SNPs per chromosome). To reduce computing times, we only kept the 200 most common SNPs (i.e. most frequent in the cPZM carrier parents) per chromosome, and a random selection of 800 individuals for further testing. Between 4,397 and 4,600 (median = 4,600) genome-wide SNPs were tested per individual. In the same way to cPZM genotypes, we extracted reads mapping to the ALT allele in each of the selected SNPs and calculated the median NM per site and individual. We found that >98% of high-quality SNPs had a median NM ≤4 (**Figure S2**), so we used this as a hard threshold to filter cPZMs genotypes.

Section 4. Generating variant-specific error rates for genomic sites harbouring cPZMs

The variant-specific error rate is an empirical measure of the expected probability of observing a read with a given allele at a specific genomic position under random noise. In this context, it reflects either sequencing errors or common variation in the GEL cohort, with low ‘error rate’ variants being a product of the first instance, and variants with high ‘error rate’ being a product of the second.

To obtain the variant-specific error for a given genomic position, we pooled mapped reads across 2,500 randomly selected unrelated trio parents, and calculated the proportion of reads mapping to each single nucleotide allele (A, T, C, G, -). A list of unrelated individuals was readily available in the Genomics England data resources[20], and was produced by

pairwise pruning of up to third-degree related individuals (e.g., cousins and great-grandparents) based on genetic relatedness scores obtained using the KING-robust algorithm. Random individuals were selected using the R $dplyr::sample\_n()$ function setting $replace = FALSE$. Reads were pooled using the R $deepSNV::loadAllData()$ function[21]. Base and mapping quality were set to a minimum of 20, and reads with the bitflag 3844 (i.e., unmapped reads, not primary alignments, reads failing vendor quality checks, optical and PCR duplicates, and supplementary alignments) were excluded. The variant-specific error rate distributions obtained using this method for the 4,733 cPZMs remaining after step 6 of the filtering are shown in **Figure S3**.

Section 5. Ensuring independent read support for final cPZM

Of the 1,015 final PZM candidates, 604 are supported by exactly two ALT-supporting reads, one on each strand, as per our filtering in **Section 2.4**. In these cases, if both reads derive from the same read pair (e.g., due to short insert sizes causing forward and reverse reads to overlap at the variant position), they would represent a single DNA fragment rather than two independent observations. To assess this, we extracted read names for all ALT-supporting reads across all 1,015 final PZM candidates, applying the same read-filtering criteria described in **Section 2.4**. For each variant, we compared the number of unique read names to the total ALT read count: if both values are equal, each ALT-supporting read derives from an independent fragment; if the unique count is lower, at least two reads share a fragment. Only 2/1,015 PZMs showed a discrepancy between unique and total ALT read counts, with only one of these having an ALT read count of exactly two (**Figure S8**). We therefore conclude that the vast majority of our final PZM candidates are supported by at least two independent DNA fragments.

Section 6. Estimation of VAF-ascertainment bias for parental PZMs selected from Mendelian inconsistencies nominated by Platypus

Our PZM ascertainment model is conditional on diploid genotype calls from the Platypus variant caller, which is not mosaic-aware and for which putative parental PZMs present at intermediate or high VAFs are more likely to be called as heterozygous and therefore excluded at the Mendelian inconsistency selection stage, resulting in an ascertainment bias toward low-VAF parental mosaics in our final PZM catalogue.

To better understand the relationship between VAF and genotype call by Platypus, we obtained the empirical probability of obtaining a heterozygous call (0/1) for a given VAF bin. For this, from a subset of Mendelian inconsistencies matching our initial PZM candidate selection (i.e., GRCh38 autosomal single nucleotide variants, not mapping to simple repeats, segmental duplications, genome gaps, and having a non-zero parental ALT coverage), we randomly selected 6,000 variants. For the selected variants, we calculated offspring and parental VAFs from the ALT coverage and the total read depth reported by Platypus. Then, we binned the resulting VAFs into 50 bins of equal size (N = 240 genotypes per bin) and obtained the proportion of variants called heterozygous in each bin (**Figure S10**). According to this analysis, variants with a VAF ≥0.14 have at least a 5% probability of being called heterozygous by Platypus, rising to 50% at VAFs ≥0.17, implying that our pipeline has an increasing likelihood of excluding genuine mosaic variants above this VAF threshold. Furthermore, this calling behaviour has direct implications for the VAF distribution observed in our final PZM catalogue. At the average sequencing depth of this cohort (~30X), variants with a VAF below 0.14 correspond to those supported by approximately 4 or fewer alternative reads. Since these are the variants most likely to be called homozygous reference by Platypus and therefore retained as Mendelian inconsistencies, our ascertainment strategy is inherently enriched for PZMs supported by a small number of alternative reads, which directly explains the monomodal VAF distribution centred around 0.05 observed in our catalogue. This ascertainment bias is therefore a predictable consequence of applying a standard germline variant caller at standard sequencing depth,

rather than an artefact of our filtering approach.an increasing likelihood of excluding genuine mosaic variants above this VAF threshold.

Section 7. Characterisation of PZMs

A. Comparing average number of early PZMs per individual across studies

For Rahbari et al. (2016), we consulted the "Table 1" in their main text, and obtained the number of autosomal (chr1:22) SNV PZMs assigned to each parent in the three families included in this study (i.e., 6 parents). For each parent, we counted mutations marked as found in parental blood (SM or SP), or in the parental blood alone (M or P). In total, this study reports 24 PZMs meeting the above criteria across six individuals, corresponding to an average of 4 PZMs per parent.

For Sassani et al. (2019), we consulted their online supplementary material (https://github.com/quinlan-lab/ceph-dnm-manuscript), specifically the "gonosomal.dnms.summary.csv" table. This included all third generation (i.e., trio offspring) candidate DNMs where second generation individuals (i.e., in trio parents), showed incomplete linkage with heterozygous SNPs from first generation individuals (i.e., trio offspring grandparents) across 69 trio parents. To obtain the mean per individual, we summed the "snv_autosomal_dnms", which totalled 442 autosomal single nucleotide PZMs and corresponded to an average of 6.40 PZMs per individual.

For Jonsson et al. (2021) we accessed the "Supplementary Data 2", available in the online version from this publication. This table includes all candidate DNMs identified using their described quadruplets approach (i.e., candidate DNMs in the proband's offspring; absent from proband's twin and spouse/partner). From this table, we kept pre-PGC PZMs following the rule described in the "*Determination of somatic presence and dating of mutations*" section from this publication's supplementary note, after which we removed variants showing

an alternative allele read support of less than 3 reads in the proband (i.e., one of the trio parents), keeping 581 PZMs. From these, and to make a fair comparison with our data, we removed INDELs and variants mapping to the chrX, keeping a total of 523 autosomal SNV PZMs. To obtain the mean per parent, we considered all of the probands included in their quadruplet approach (n = 451).

For our study, we obtained the global average across all individuals that were not intentionally excluded (n = 123), totalling 21,521 individuals out of an initial selection of 12,644 parents. Individuals were excluded due to: a) presenting a form of true or false positive hypermutation, as reported by Kaplanis et al., (2022)[3], or b) participant ID inconsistencies in GEL data that prevented indexing metadata. Altogether, we identified an average PZM mutation rate of 0.046 PZMs per parent.

B. Association between parental ages at conception and trio DNMs and PZMs

DNMs detected in a trio are known to be associated with parental ages at conception, since this type of mutation accumulates with age in single gametes and gamete progenitor cells in the case of males[22]. Since parental PZMs are expected to have originated during the parental embryonic development, a correlation between the total number of parental PZMs detected in an offspring and the parental age at conception is not expected. To corroborate this in our data, for each trio we obtained PZMs per trio, by summing the PZMs ascertained from the mother and father. We accounted for the de-duplication of PZMs performed earlier in our filtering pipeline, and we assigned sibling-shared PZMs to the corresponding offspring (n = 20). For all offspring carrying at least one PZMs (n = 955), we obtained the unphased DNMs counts. These corresponded to a set of pre-filtered and bioinformatically ascertained per trio DNMs, previously reported by Kaplanis et al. (2022). We then used a generalised linear regression model from the “Poisson” family to model mutation counts (either total DNMs or PZMs) as a function of parental ages at conception while controlling technical covariates that could have affected the mutation calling process, as follows:

$$mutation\ counts = \beta_0 + maternal\ age\ at\ conception \bullet \beta_1 + $$
$$+ paternal\ age\ at\ conception \bullet \beta_2 + $$
$$mean\ sequencing\ depth_{mother} \bullet \beta_3 + mean\ sequencing\ depth_{father} \bullet \beta_4 + $$
$$mean\ sequencing\ depth_{offspring} \bullet \beta_6 + $$
$$percent\ aligned\ reads_{mother} \bullet \beta_5 + percent\ aligned\ reads_{father} \bullet \beta_6 + $$
$$percent\ aligned\ reads_{offspring} \bullet \beta_7 + \varepsilon$$

We found no significant association between PZM counts per trio and maternal or paternal ages at conception (p-value = 0.54, p-value = 0.420, respectively), while these were significantly associated with DNM counts (maternal effect = 7.2×10^{-3}, p-value = 6.37×10^{-14}; paternal age effect 1.8×10^{-2}, p-value < 2×10^{-16}).

C. Mutation spectra annotation of PZMs

We annotated each SNV using according to the pyrimidine change caused by the ALT allele (i.e., C>A, C>T, C>G, T>A, T>C, T>G). In addition, we differentiate between C>T sites occurring in CpG sites (CpG>TpG) and elsewhere in the genome, to account for the differential number of transitions of these genomic contexts[30,31]. Using the seven pyrimidine substitution classifications outlined above, we calculated differences across sexes and DNM events. For each group comparison (i.e., paternal vs maternal PZMs; DNMs vs PZMs), we estimated if proportions of mutations assigned to each mutation category were significantly different using the Fisher's exact test to obtain per pyrimidine substitution Odds Ratios (OR), 95% confidence intervals, and p-value for ORs. P-values for each experiment were adjusted to account for multiple comparisons using the FDR method. Since our PZM filtering explicitly removes variants at gnomAD AFs > 0.1%, we performed the same operation for GEL DNMs since "common" DNMs could be enriched on CpG>TpG transitions, which could bias our detected PZM/DNM differences.

To test if PZMs and DNMs have a different mutational origin, as defined by their mutation spectra, we performed mutational signature extraction using SigProfiler[32]. For this, we annotated the pyrimidine substitutions obtained above with their 5' and 3' contexts and generated counts matrices for the resulting combinations (n = 96). We used this as input for

SigProfiler extractor to estimate *de novo* mutational signatures. We deconvoluted the resulting signatures into reference signatures reported in the COSMIC[33] database using the relevant method in the SigProfiler software.

D. PZM/DNM mutation spectra comparison with Sasani et al. (2019) and Jonsson et al. (2021)

Sasani et al. (2019) and Jonsson et al., (2021) ascertained PZMs from candidate DNMs at whole genome scale, which can be used to contrast the mutation spectra of PZMs in our catalogue, although with some caveats. Both of the mentioned studies featured extended family-trio study designs, including combinations of parents, parental monozygotic twins, offspring siblings and/or grandparents, which allowed them to nominate PZM candidates across broader developmental windows, including those occurring around the first cell division, and having a VAF close to 0.5 in parental blood, or close and even after the differentiation of the progenitor germ cells, thus having a very low (or even null) VAF in the parental blood. Further, the use of specific extended family trio study designs (e.g., offspring, parents, and grandparents - i.e., three-generation) biases the detection of PZMs occurring at specific developmental windows, which is reflected on the VAF distribution of PZMs discovered under each strategy (**Figure S12**).

In summary, Sasani et al., (2019) detected PZMs applying WGS (~30X) in a three generation cohort including offspring, parents and grandparents (third, second, and first generation, respectively). They first detected DNM candidates present in second generation individuals at incomplete linkage with heterozygous SNPs from first generation individuals, and that were constitutively inherited by third generation individuals. This effectively ascertains PZMs likely occurring before the differentiation of PGCs in second generation individuals, and that are present as mosaics in both somatic and germline tissues. Secondly, they detected DNM candidates in third generation individuals that were shared by two or more siblings in the same family and that present no evidence of ALT read support in second

generation individuals. This effectively ascertains PZMs that occurred after or around the differentiation of PGCs, and are likely to be confined to the germline tissue in second generation individuals. Using their three generation approach, they detected 442 PZMs (median parental VAF = 0.241; parental VAF range: 0.10-0.67) and 720 DNMs using their sibling shared DNM approach. Since the latter represent events mostly confined to the germline tissue, the VAF of these events in parental somatic tissues is either null or undetectable using standard variant filtering criteria.

Jonsson et al. (2021) detected PZMs applying WGS (~30X) in a cohort of families of monozygotic twins. First, they detected PZMs using quadruplets, which comprise a standard two-generation family-trio, but where one of the parents (i.e., the proband) has a monozygotic. In this setting, they first detected PZMs by identifying DNM candidates in the trio offspring for which there was evidence of ALT read support in the proband but not their twin. Secondly, for a subset of twins whose parents had sequencing data available, they identified PZMs by identifying DNM candidates in the trio offspring for which there was evidence of ALT read support in both twins but not their parents, or the proband's partner. Both of these strategies effectively ascertain PZMs occurring before the differentiation of PGCs, but after or prior twinning, and thus later or earlier during development, respectively. Using their quadruplet and three generation approaches, Jonsson et al. (2021) detected 529 PZMs (median parental VAF = 0.173; parental VAF range: 0.01, 0.63), and 102 PZMs (median parental VAF = 0.176; parental VAF range: 0.02, 0.53).

In contrast to these extended designs, in our study PZM ascertaining relies on WGS (~30X) of only two-generation family trios, genotypes designated as REF homozygous in parents (mostly supported by ~2 reads), and include several steps aiming to reduce the chances of including inherited parental constitutive variants. This resulted in ascertainment bias towards PZMs occurring before the differentiation of PGCs, but likely after the first cell divisions, displaying a VAF range of 0.02-0.34 (median = 0.049) in parental blood.

Taking all of the above into account, we compared the pyrimidine substitution spectra between DNMs and PZMs in the GEL, Sasani et al. (2019), and Jonsson et al. (2021) datasets as described in the “Mutation spectra annotation of PZMs” section from these supplementary methods. Since the PZM ascertainment strategy influences the power to identify PZMs occurring at specific developmental timings, we split the PZMs in Sasani and Jonsson according to the type of extended family trio used to identify them. Since each study generated their own DNM catalogues, we compared PZMs in each study to their own DNM candidates to prevent batch effects. The results from each comparison are shown in **Table S2.**

We found that our catalogue recapitulated the C>A enrichment and T>C depletion of PZMs relative to DNMs reported by both Sasani and Jonsson in their PZM subsets enriched for mutations arising at later developmental stages. Notably, our catalogue also recapitulates a T>A depletion in PZMs relative to DNMs, a signal reported by Sasani et al. (2019) specifically in their earlier-development PZM group. Although our ascertainment strategy is underpowered to systematically detect high-VAF PZMs due to stringent parental genotyping filtering, we nonetheless detect a subset of such events, and this minority is sufficient to recapitulate the T>A depletion observed in Sasani et al.'s early development group (n = 442, median VAF = 0.241), which is better powered than the equivalent group in Jonsson et al. (n = 102, median VAF = 0.176). Together, these results suggest that our catalogue is enriched for PZMs arising at later developmental stages, while still retaining genuine spectral signals from earlier developmental events, lending biological credibility to the observed mutation spectra differences and confirming that our catalogue captures true PZM events despite its design constraints.

E. Testing genomic clustering of PZMs and DNMs

To assess whether mutations (PZMs or DNMs) are clustered in specific genomic regions, we performed a permutation test on binned mutation counts. We first divided the genome into

consecutive two-megabase (Mb) windows and counted the number of mutations in each bin. To generate the null distribution, we randomly reassigned the total number of observed mutations across all bins, while accounting for differences in bin size due to mappability and filtering.

Specifically, we adjusted for the callable size of each bin by subtracting the number of bases overlapping regions excluded during filtering (e.g., segmental duplications, centromeres). We then computed a per-bin probability of receiving a mutation as the callable size of the bin divided by the total callable genome size. This weighted random assignment was repeated 10,000 times to generate a null distribution of mutation counts per bin. Finally, we computed one-tailed empirical p-values per bin as the proportion of permutations in which the number of mutations assigned to a bin was equal to or greater than the observed count **(Figure S13)**. We corrected our p-values to account for multiple testing using the "Benjamini-Hochberg" method.

F. Testing the association of genomic features with clustered PZMs and DNMs

Using the bin definition outlined in the previous section, we tested whether mutation-enriched bins had differential genomic features as defined by GC content and replication timing (RT). We annotated GC content per bin by extracting the GRCh38 sequences from each bin using the R libraries GenomicRanges and Biostrings[23,24]. For each bin, we counted the total number of non "N" nucleotides and determined the GC content as the proportion of "G" and "C" nucleotides out of this total. RTs were obtained from Massey et al., (2019)[25]. These were derived from the GM12878 lymphoblastoid cell line from which RT was inferred for the S-phase sorted cells, using G1-sorted cells as baseline[25]. This profile was selected as it correlates with RT across at least 50% of profiled cell types[26,27].

We modelled the likelihood of a bin being enriched for mutations as a function of GC content, RT, and callable size using a generalised linear model with a binomial link function. Coefficients represent the change in odds of bin enrichment per unit increase in each

predictor. Because no PZM bins survived FDR correction (see **Section 6E**), enriched bins for PZMs were defined using a nominal permutation $p \leq 0.05$ threshold (n = 49). For DNMs, where sufficient bins passed multiple testing correction, enriched bins were defined at FDR-adjusted permutation $p \leq 0.05$ (n = 236). Applying this analysis to DNM-enriched bins yielded a positive association with GC content (OR = 1.08, $p = 2.15 \times 10^{-4}$) and a negative association with RT (OR = 0.60, $p = 9.58 \times 10^{-7}$) consistent with prior reports[28,29] and supporting the robustness of our approach for detecting genuine genomic feature associations.

G. Variant functional annotation and identification of potentially clinically relevant PZMs

We used the Ensembl variant effect predictor (VEP, version 112) to generate functional effect predictions for PZMs[34]. We used bcftools +split-vep plugin[35] to keep the transcript for which the worst consequence was found. VAF differences across VEP categories were assessed using a linear regression model of the gamma family to account for the skewed VAF distribution of PZMs. We first modelled VAF as a function of six VEP categories, namely: LoF, missense, splice, synonymous, UTR, and non-coding variants. We also modelled VAF as a function of variants found overlapping coding exons or not (exonic, non-exonic variants). None of the tested categories was significantly associated with PZMs VAF (p-value >0.05).

We also tested enrichment of PZMs across genomic regions predicted to have specific functions. For this, we assigned genomic coordinates (GRCh38) to one of three categories: transcriptional units (i.e., introns, exons, UTR regions), putative regulatory regions (5000 bases upstream transcription start sites or downstream transcription termination sites), and intergenic regions. Coordinates for these elements were inferred from exon, intron, and UTR coordinates from the Refseq MANE select coding transcripts, obtained from the UCSC table browser[36]. Then we used the genomic association tester (GAT) algorithm to test if the

observed number PZMs overlapping each annotation was significantly different from expectation under random chance[37].

To identify PZMs with potential clinical value, we looked at those whose VEP functional impact was classed as either HIGH or MODERATE (n = 21), and extracted the HUGO symbols for the affected genes. Then, we intersected these genes with PanelApp genes (n = 6,461)[38], keeping 12 genes (one per PZM variant) for further inspection. We then kept PZMs in genes found in gene panels in the “green” category (i.e., those with definitive evidence of causality for disease[38]), and that have a dominant effect in disease (i.e., monoallelic inheritance) according to the same resource. This resulted in the identification of five genes *(VAMP2, WT1, DYNC1H1, PHIP,* and *NLRP3*). From these, we followed up genes whose variant carrier was classed as “undiagnosed” in GEL records. We discarded *NLRP3* since the proband was reported to have been diagnosed on the basis of a different gene variant. For the rest, we inspected individual phenotype records, and compared these against the phenotypes reported by PanelApp and in the literature. We discarded *PHIP* from further description since the individuals’s phenotypes were not a good match with Chung-Jansen Syndrome, associated with *PHIP* mutations[39].

The remaining three variants, all missense, in *VAMP2*, *WT1*, and *DYNC1H1*, were assessed based on the pathogenicity evidence given under the ACMG standards and guidelines[40] according to the January 2018 ClinGen SVI Bayesian classification framework[41]. For all of these cases, the PS2 criteria was applied at reduced strength (moderate) since probands are heterozygous and parents are low level mosaics and clinically unaffected. The ACMG criteria applied to each variant are detailed in **Table S3**.

Supplemental references

1. Rimmer, A., Phan, H., Mathieson, I., Iqbal, Z., Twigg, S.R.F., WGS500 Consortium, Wilkie, A.O.M., McVean, G., and Lunter, G. (2014). Integrating mapping-, assembly- and haplotype-based approaches for calling variants in clinical sequencing applications. Nat. Genet. *46*, 912–918. https://doi.org/10.1038/ng.3036.

2. Caulfield, M., Davies, J., Dennys, M., Elbahy, L., Fowler, T., Hill, S., Hubbard, T., Jostins, L., Maltby, N., Mahon-Pearson, J., et al. (2020). National Genomic Research Library. Preprint at figshare.

3. Kaplanis, J., Ide, B., Sanghvi, R., Neville, M., Danecek, P., Coorens, T., Prigmore, E., Short, P., Gallone, G., McRae, J., et al. (2022). Genetic and chemotherapeutic influences on germline hypermutation. Nature *605*, 503–508. https://doi.org/10.1038/s41586-022-04712-2.

4. Karolchik, D., Hinrichs, A.S., Furey, T.S., Roskin, K.M., Sugnet, C.W., Haussler, D., and Kent, W.J. (2004). The UCSC Table Browser data retrieval tool. Nucleic Acids Res. *32*, D493–D496. https://doi.org/10.1093/nar/gkh103.

5. 1000 Genomes Project Consortium, Auton, A., Brooks, L.D., Durbin, R.M., Garrison, E.P., Kang, H.M., Korbel, J.O., Marchini, J.L., McCarthy, S., McVean, G.A., et al. (2015). A global reference for human genetic variation. Nature *526*, 68–74. https://doi.org/10.1038/nature15393.

6. Khanna, A., Larson, D.E., Srivatsan, S.N., Mosior, M., Abbott, T.E., Kiwala, S., Ley, T.J., Duncavage, E.J., Walter, M.J., Walker, J.R., et al. (2021). Bam-readcount - rapid generation of basepair-resolution sequence metrics. ArXiv *7*, 3722. https://doi.org/10.21105/joss.03722.

7. 1. Introduction — Platypus 0.1.5 documentation https://rahmanteamdevelopment.github.io/Platypus/documentation.html.

8. Lindenbaum, P. (2015). JVarkit: java-based utilities for Bioinformatics. Preprint at figshare, https://doi.org/10.6084/M9.FIGSHARE.1425030 https://doi.org/10.6084/M9.FIGSHARE.1425030.

9. Stoler, N., and Nekrutenko, A. (2021). Sequencing error profiles of Illumina sequencing instruments. NAR Genom. Bioinform. *3*, lqab019. https://doi.org/10.1093/nargab/lqab019.

10. Chen, S., Francioli, L.C., Goodrich, J.K., Collins, R.L., Kanai, M., Wang, Q., Alföldi, J., Watts, N.A., Vittal, C., Gauthier, L.D., et al. (2024). A genomic mutational constraint map using variation in 76,156 human genomes. Nature *625*, 92–100. https://doi.org/10.1038/s41586-023-06045-0.

11. Wang, J., Raskin, L., Samuels, D.C., Shyr, Y., and Guo, Y. (2015). Genome measures used for quality control are dependent on gene function and ancestry. Bioinformatics *31*, 318–323. https://doi.org/10.1093/bioinformatics/btu668.

12. Sasani, T.A., Pedersen, B.S., Gao, Z., Baird, L., Przeworski, M., Jorde, L.B., and Quinlan, A.R. (2019). Large, three-generation human families reveal post-zygotic mosaicism and variability in germline mutation accumulation. Elife *8*, e46922. https://doi.org/10.7554/eLife.46922.

13. Xia, J., Wang, Q., Jia, P., Wang, B., Pao, W., and Zhao, Z. (2012). NGS catalog: A

database of next generation sequencing studies in humans. Hum. Mutat. *33*, E2341–E2355. https://doi.org/10.1002/humu.22096.

14. van Neerven, S.M., and Vermeulen, L. (2023). Cell competition in development, homeostasis and cancer. Nat. Rev. Mol. Cell Biol. *24*, 221–236. https://doi.org/10.1038/s41580-022-00538-y.

15. Castillo-Venzor, A., Penfold, C.A., Morgan, M.D., Tang, W.W., Kobayashi, T., Wong, F.C., Bergmann, S., Slatery, E., Boroviak, T.E., Marioni, J.C., et al. (2023). Origin and segregation of the human germline. Life Sci. Alliance *6*. https://doi.org/10.26508/lsa.202201706.

16. The SAM/BAM Format Specification Working Group Sequence alignment/map format specification. https://samtools.github.io/hts-specs/SAMv1.pdf.

17. Eberle, M.A., Fritzilas, E., Krusche, P., Källberg, M., Moore, B.L., Bekritsky, M.A., Iqbal, Z., Chuang, H.-Y., Humphray, S.J., Halpern, A.L., et al. (2017). A reference data set of 5.4 million phased human variants validated by genetic inheritance from sequencing a three-generation 17-member pedigree. Genome Res. *27*, 157–164. https://doi.org/10.1101/gr.210500.116.

18. Zook, J. (2012). Genome in a Bottle | NIST.

19. Aggregated Variant Calls (AggV2) - Genomics England Research Environment User Guide https://re-docs.genomicsengland.co.uk/aggv2/.

20. AggV2 Principal Components and genetically inferred relatedness - Genomics England Research Environment User Guide https://re-docs.genomicsengland.co.uk/principal_components/.

21. Gerstung, M., Beisel, C., Rechsteiner, M., Wild, P., Schraml, P., Moch, H., and Beerenwinkel, N. (2012). Reliable detection of subclonal single-nucleotide variants in tumour cell populations. Nat. Commun. *3*, 811. https://doi.org/10.1038/ncomms1814.

22. Moore, L., Cagan, A., Coorens, T.H.H., Neville, M.D.C., Sanghvi, R., Sanders, M.A., Oliver, T.R.W., Leongamornlert, D., Ellis, P., Noorani, A., et al. (2021). The mutational landscape of human somatic and germline cells. Nature *597*, 381–386. https://doi.org/10.1038/s41586-021-03822-7.

23. Lawrence, M., Huber, W., Pagès, H., Aboyoun, P., Carlson, M., Gentleman, R., Morgan, M.T., and Carey, V.J. (2013). Software for computing and annotating genomic ranges. PLoS Comput. Biol. *9*, e1003118. https://doi.org/10.1371/journal.pcbi.1003118.

24. Biostrings Bioconductor. https://bioconductor.org/packages/release/bioc/html/Biostrings.html.

25. Massey, D.J., Kim, D., Brooks, K.E., Smolka, M.B., and Koren, A. (2019). Next-generation sequencing enables spatiotemporal resolution of human centromere replication timing. Genes (Basel) *10*, 269. https://doi.org/10.3390/genes10040269.

26. Hansen, R.S., Thomas, S., Sandstrom, R., Canfield, T.K., Thurman, R.E., Weaver, M., Dorschner, M.O., Gartler, S.M., and Stamatoyannopoulos, J.A. (2010). Sequencing newly replicated DNA reveals widespread plasticity in human replication timing. Proc. Natl. Acad. Sci. U. S. A. *107*, 139–144. https://doi.org/10.1073/pnas.0912402107.

27. Ryba, T., Hiratani, I., Lu, J., Itoh, M., Kulik, M., Zhang, J., Schulz, T.C., Robins, A.J.,

Dalton, S., and Gilbert, D.M. (2010). Evolutionarily conserved replication timing profiles predict long-range chromatin interactions and distinguish closely related cell types. Genome Res. *20*, 761–770. https://doi.org/10.1101/gr.099655.109.

28. Besenbacher, S., Sulem, P., Helgason, A., Helgason, H., Kristjansson, H., Jonasdottir, A., Jonasdottir, A., Magnusson, O.T., Thorsteinsdottir, U., Masson, G., et al. (2016). Multi-nucleotide de novo mutations in humans. PLoS Genet. *12*, e1006315. https://doi.org/10.1371/journal.pgen.1006315.

29. Acuna-Hidalgo, R., Veltman, J.A., and Hoischen, A. (2016). New insights into the generation and role of de novo mutations in health and disease. Genome Biol. *17*, 241. https://doi.org/10.1186/s13059-016-1110-1.

30. Rahbari, R., Wuster, A., Lindsay, S.J., Hardwick, R.J., Alexandrov, L.B., Turki, S.A., Dominiczak, A., Morris, A., Porteous, D., Smith, B., et al. (2016). Timing, rates and spectra of human germline mutation. Nat. Genet. *48*, 126–133. https://doi.org/10.1038/ng.3469.

31. Cooper, D.N., and Krawczak, M. (1989). Cytosine methylation and the fate of CpG dinucleotides in vertebrate genomes. Hum. Genet. *83*, 181–188. https://doi.org/10.1007/BF00286715.

32. Islam, S.M.A., Díaz-Gay, M., Wu, Y., Barnes, M., Vangara, R., Bergstrom, E.N., He, Y., Vella, M., Wang, J., Teague, J.W., et al. (2022). Uncovering novel mutational signatures by de novo extraction with SigProfilerExtractor. Cell Genom *2*, None. https://doi.org/10.1016/j.xgen.2022.100179.

33. Sondka, Z., Dhir, N.B., Carvalho-Silva, D., Jupe, S., Madhumita, McLaren, K., Starkey, M., Ward, S., Wilding, J., Ahmed, M., et al. (2024). COSMIC: a curated database of somatic variants and clinical data for cancer. Nucleic Acids Res. *52*, D1210–D1217. https://doi.org/10.1093/nar/gkad986.

34. McLaren, W., Gil, L., Hunt, S.E., Riat, H.S., Ritchie, G.R.S., Thormann, A., Flicek, P., and Cunningham, F. (2016). The Ensembl Variant Effect Predictor. Genome Biol. *17*, 122. https://doi.org/10.1186/s13059-016-0974-4.

35. Danecek, P., Bonfield, J.K., Liddle, J., Marshall, J., Ohan, V., Pollard, M.O., Whitwham, A., Keane, T., McCarthy, S.A., Davies, R.M., et al. (2021). Twelve years of SAMtools and BCFtools. Gigascience *10*. https://doi.org/10.1093/gigascience/giab008.

36. Perez, G., Barber, G.P., Benet-Pages, A., Casper, J., Clawson, H., Diekhans, M., Fischer, C., Gonzalez, J.N., Hinrichs, A.S., Lee, C.M., et al. (2024). The UCSC Genome Browser database: 2025 update. Nucleic Acids Res. https://doi.org/10.1093/nar/gkae974.

37. Heger, A., Webber, C., Goodson, M., Ponting, C.P., and Lunter, G. (2013). GAT: a simulation framework for testing the association of genomic intervals. Bioinformatics *29*, 2046–2048. https://doi.org/10.1093/bioinformatics/btt343.

38. Stark, Z., Foulger, R.E., Williams, E., Thompson, B.A., Patel, C., Lunke, S., Snow, C., Leong, I.U.S., Puzriakova, A., Daugherty, L.C., et al. (2021). Scaling national and international improvement in virtual gene panel curation via a collaborative approach to discordance resolution. Am. J. Hum. Genet. *108*, 1551–1557. https://doi.org/10.1016/j.ajhg.2021.06.020.

39. de Fallois, J., Sieckmann, T., Schönauer, R., Petzold, F., Münch, J., Pauly, M., Vasileiou,

G., Findeisen, C., Kampmeier, A., Kuechler, A., et al. (2024). Pathogenic PHIP variants are variably associated with CAKUT. Kidney Int. Rep. *9*, 2484–2497. https://doi.org/10.1016/j.ekir.2024.05.024.

40. Richards, S., Aziz, N., Bale, S., Bick, D., Das, S., Gastier-Foster, J., Grody, W.W., Hegde, M., Lyon, E., Spector, E., et al. (2015). Standards and guidelines for the interpretation of sequence variants: a joint consensus recommendation of the American College of Medical Genetics and Genomics and the Association for Molecular Pathology. Genet. Med. *17*, 405–424. https://doi.org/10.1038/gim.2015.30.

41. Tavtigian, S.V., Greenblatt, M.S., Harrison, S.M., Nussbaum, R.L., Prabhu, S.A., Boucher, K.M., Biesecker, L.G., and ClinGen Sequence Variant Interpretation Working Group (ClinGen SVI) (2018). Modeling the ACMG/AMP variant classification guidelines as a Bayesian classification framework. Genet. Med. *20*, 1054–1060. https://doi.org/10.1038/gim.2017.210.

42. Bogue, D., Ryan, G., Wassmer, E., Research Consortium, G.E., and Naik, S. (2023). VAMP2 gene-related neurodevelopmental disorder: A differential diagnosis for Rett/Angelman-type spectrum of disorders. Mol. Syndromol. *14*, 449–456. https://doi.org/10.1159/000530150.

43. Cuccurullo, C., Cerulli Irelli, E., Ugga, L., Riva, A., D’Amico, A., Cabet, S., Lesca, G., Bilo, L., Zara, F., Iliescu, C., et al. (2024). Clinical features and genotype-phenotype correlations in epilepsy patients with de novo DYNC1H1 variants. Epilepsia *65*, 2728–2750. https://doi.org/10.1111/epi.18054.
